# DYRK1A enhances antitumor immunity in type 1 conventional dendritic cells via mTORC1 activation

**DOI:** 10.1172/JCI199108

**Published:** 2026-04-23

**Authors:** Hongjiao Wang, He Jiang, Songlin He, Songwen Ren, Haiwen Li, Wangnan Liu, Chunyun Zhou, Pan Zhu, Keren Chen, Weijia Cao, Yan Qin, Du, Nengming Xiao, Hongling Huang, Chun-Jung Ko, Yiming Zheng, Bo Wang, Qiang Zou, Jian-Hong Shi, Xun Li, Zuliang Jie

**Affiliations:** 1 State Key Laboratory of Cellular Stress Biology, Department of Oncology, Xiang’an Hospital of Xiamen University, School of Life Sciences, Faculty of Medicine and Life Sciences, Xiamen University, Xiamen, Fujian, China.; 2 Department of Laboratory Medicine, Xiamen Key Laboratory of Genetic Testing, The First Affiliated Hospital of Xiamen University, School of Medicine, Xiamen University, Xiamen, Fujian, China.; 3 Central Laboratory, Hebei Collaborative Innovation Center of Tumor Microecological Metabolism Regulation, Affiliated Hospital of Hebei University, Clinical Medical College, Hebei University, Baoding, Hebei, China.; 4 School of Public Health and; 5 Department of Stomatology, Cancer Research Center, School of Medicine, Xiamen University, Xiamen, Fujian, China.; 6 Graduate Institute of Immunology, College of Medicine, National Taiwan University, Taipei, Taiwan.; 7 Shanghai Institute of Immunology, Department of Immunology and Microbiology, Shanghai Jiao Tong University School of Medicine, Shanghai, China.; 8 State Key Laboratory of Vaccines for Infectious Diseases, Xiang An Biomedicine Laboratory, School of Public Health, Xiamen University, Xiamen, Fujian, China.

**Keywords:** Cell biology, Immunology, Dendritic cells

## Abstract

Type 1 conventional dendritic cells (cDC1s) play an integral role in mediating immune responses and maintaining homeostasis, yet the molecular mechanisms underlying their functions remain poorly understood. In this study, we identified dual-specificity tyrosine phosphorylation-regulated kinase 1A (DYRK1A) as a key kinase that responded to TLR and growth factor stimulation and acted as an essential regulator of cDC1 function. Genetic ablation of *Dyrk1a* specifically in cDC1s impaired antitumor immunity and accelerated tumor progression in murine models. Mechanistically, DYRK1A mediated the phosphorylation of the mTOR complex 1 (mTORC1) inhibitor TSC2 at serine 540, triggering the degradation of TSC2 and promoting mTORC1 signaling in cDC1s. Notably, *Tsc2* deletion in *Dyrk1a*-deficient cDC1s remarkably restored their antitumor immune functions. Furthermore, DYRK1A-mediated mTORC1 signaling in cDC1s positively correlated with effector T cell responses across multiple human cancers. Our findings highlight a critical role for the DYRK1A/TSC2/mTORC1 signaling pathway in regulating cDC1 functions in antitumor immunity, offering potential strategies to improve cancer immunotherapy.

## Abstract 2

This study highlights a critical role for the DYRK1A-TSC2-mTORC1 signaling pathway in regulating cDC1 functions in antitumor immunity, offering potential strategies to improve cancer immunotherapy.

## Graphical abstract

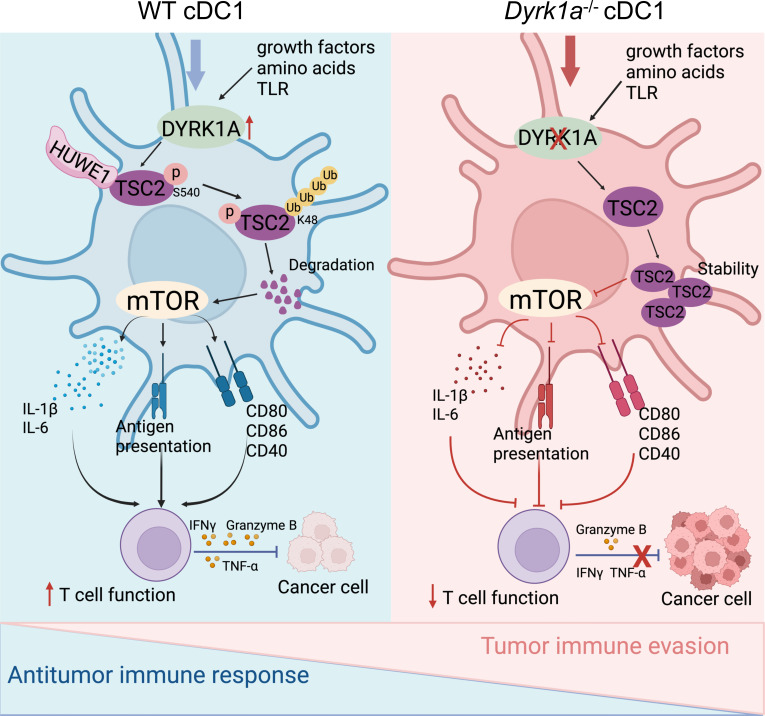

## Introduction

As professional antigen-presenting cells, dendritic cells (DCs) play an essential role in initiating antitumor immune responses (1–3). They primarily sense the microenvironment through pattern recognition receptors, which recognize diverse molecular patterns associated with pathogens and commensal microbes (4–6). DCs take up and present tumor-associated antigens (TAAs) to promote the differentiation and expansion of effector T cells, particularly Th1 and CD8^+^ T cells (7–11). Notably, DCs can promote either immunity or tolerance by providing immunomodulatory signals through cell-cell interactions and cytokine release (12–15). Although DCs can activate potent antitumor T cells through numerous mechanisms, they can also be hijacked by tumor-mediated factors, leading to immune tolerance and tumor progression (16–18). Despite these important findings, the signaling network regulating DC function in the tumor microenvironment (TME) remains poorly understood.

Tumor cells disrupt DC function through multiple mechanisms, resulting in inadequate T cell activation and the induction of immune tolerance toward TAAs (16, 19). Notably, metabolites within the TME profoundly impair DC functionality. For example, lactic acid, a by-product of tumor cell metabolism, suppresses DC antigen presentation to CD8^+^ T cells in lung cancer (20). Beyond acidifying the TME, tumor progression generates hypoxic niches that are substantially enriched in Tregs and cDC2s in hepatocellular carcinoma, where these cDC2s exhibit marked downregulation of HLA-DR expression (21). Consequently, these tumor-derived factors drive DC reprogramming, thereby impairing their function within the TME (8, 17). Among DC subsets, type 1 conventional dendritic cells (cDC1s) have attracted considerable research interest because of their unparalleled capacity to cross-present tumor antigens and prime CD8^+^ T cell responses, thereby serving as pivotal orchestrators of antitumor immunity (3, 22). Given the growing emphasis on therapeutic strategies to augment DC/cDC1-mediated antitumor immunity, elucidating the spatiotemporal dynamics of these cells in tumors and patients is essential. Further investigation into the mechanistic interplay between DCs (particularly cDC1s) and the TME, as well as strategies to recalibrate these interactions for enhanced immune efficacy, represents a critical frontier in cancer immunology.

Dual-specificity tyrosine phosphorylation-regulated kinase 1A (DYRK1A) is a serine/threonine kinase belonging to the evolutionarily conserved CMGC protein kinase superfamily (23, 24). Initially implicated in Down syndrome and neurodegenerative disorders (25, 26), DYRK1A has been extensively studied in neurological pathologies, where it modulates key processes such as cell cycle progression (27–29), transcription (30), RNA splicing (31), apoptosis (32), and mitochondrial biogenesis (33). Recent studies have expanded its functional scope to lymphoid development and immune regulation (28, 34–36). For instance, DYRK1A governs lymphoid development by destabilizing cyclin D3 (28), promotes autoimmunity by modulating B cell–activating factor–induced (BAFF-induced) noncanonical NF-κB activation (35), and contributes to B cell acute lymphoblastic leukemia and class switch recombination (34, 36). Despite remarkable progress in understanding DYRK1A’s functions across diverse cell types, its specific role in DCs, or their subsets, during immune responses remains poorly defined. Moreover, whether DYRK1A-mediated regulatory mechanisms modulate DC subset function in antitumor immunity remains to be determined.

Through an integrated approach combining genetic mouse models, biochemical analyses, and pharmacological interventions, we identify the DYRK1A/TSC2/mTOR complex 1 (mTORC1) axis as a critical pathway governing cDC1 function in tumor immunity. Genetic depletion of DYRK1A substantially compromises DC-mediated immune responses, leading to impaired antitumor immunity. Notably, DYRK1A exhibits subset specificity, preferentially enhancing cDC1 maturation and antigen presentation with minimal effects on cDC2s. Mechanistically, DYRK1A deficiency in cDC1s impairs antigen-specific T cell responses, thereby accelerating tumor progression. At the molecular level, DYRK1A phosphorylates the mTORC1 inhibitor TSC2, triggering destabilization of the TSC complex and subsequent activation of the mTORC1 pathway in cDC1s. Functionally, *Tsc2* ablation fully rescues the immunostimulatory capacity of *Dyrk1a*-deficient cDC1s. Clinically, The Cancer Genome Atlas (TCGA) analyses reveal strong positive correlations between DYRK1A expression/cDC1 signature genes and improved patient survival across multiple cancer types. Collectively, our study elucidates a DYRK1A/TSC2/mTORC1 axis that governs cDC1 activation and function in tumor immunity, positioning DYRK1A as a pivotal regulator of cDC1/T cell crosstalk in the TME.

## Results

### Ablation of DYRK1A in DC perturbs antitumor immunity.

**Figure 1:**
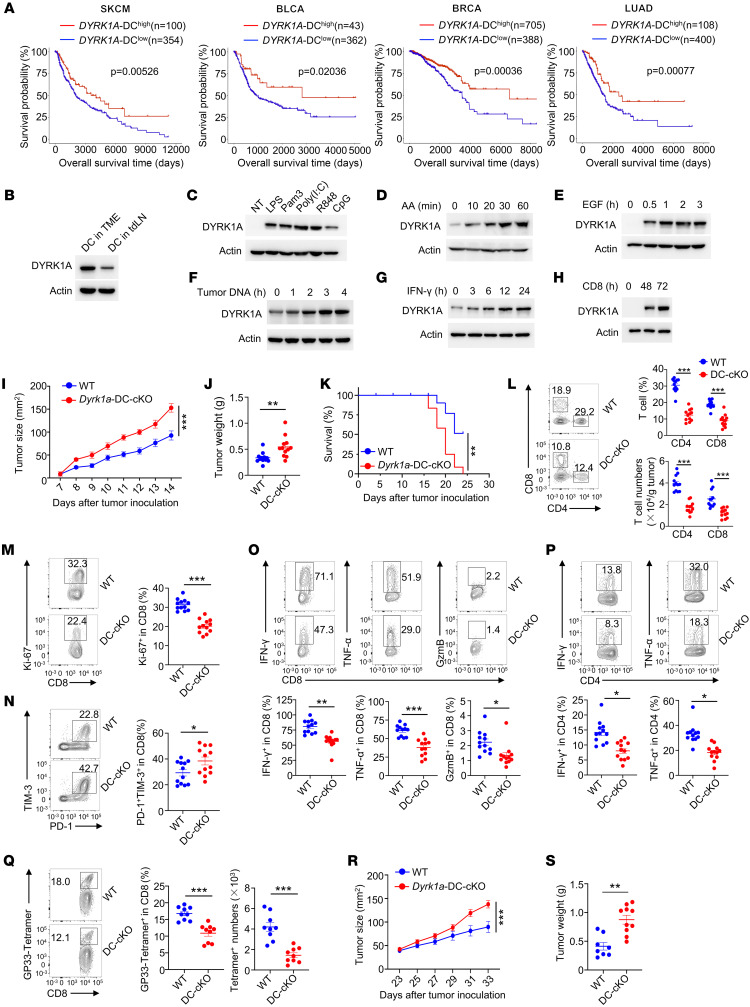
DC-specific deletion of DYRK1A impairs antitumor immunity. (**A**) Overall survival of TCGA skin cutaneous melanoma (SKCM), bladder cancer (BLCA), breast cancer (BRCA), and lung adenocarcinoma (LUAD) cohorts based on the expression levels of DYRK1A and DC signature genes. *P* values (Wald’s χ^2^ test) were determined based on a univariate Cox proportional-hazards model (*DYRK1A*-DC^hi^ versus *DYRK1A*-DC^lo^). (**B**) Immunoblot of DYRK1A in tumor and tumor-draining lymph node (tdLN) DCs. (**C**–**H**) Immunoblot of DYRK1A in BMDCs stimulated with TLR agonists (**C**), amino acids (**D**), EGF (**E**), tumor DNA (**F**), or IFN-γ (**G**) or cocultured with CD8^+^ T cells (**H**) at the indicated time points. (**I**–**K**) Tumor growth curve (**I**), tumor weight (**J**), and survival curve (**K**) of wild-type and *Dyrk1a*-DC-cKO mice subcutaneously injected with B16-F10 melanoma cells (*N* = 12 mice/group). (**L**) Frequency and number of tumor-infiltrating CD4^+^ and CD8^+^ T cells analyzed by flow cytometry. (**M** and **N**) Flow cytometric analysis of Ki-67 (**M**) and TIM-3 and PD-1 (**N**) levels of tumor-infiltrating CD8^+^ T cells. (**O** and **P**) Flow cytometric analysis of the frequency of IFN-γ–, TNF-α–, and granzyme B–producing CD8^+^ T (**O**) and CD4^+^ T cells (**P**) in tumors of wild-type and *Dyrk1a*-DC-cKO mice. (**Q**) Flow cytometric analysis of the frequency and number of GP33 tetramer^+^CD8^+^ T cells in the tumors (*N* = 9 mice/group). (**R** and **S**) Tumor growth curve (**R**) and tumor weight (**S**) of wild-type and *Dyrk1a*-DC-cKO mice subcutaneously injected with MC38 cells. Wild-type mice: *N* = 8; *Dyrk1a*-DC-cKO mice: *N* = 10. Data are representative of 3 independent experiments. Summary data are shown as the mean ± SEM. *P* values were determined using a 2-tailed unpaired Student’s *t* test (**J**, **L**–**Q**, and **S**), 2-sided log-rank Mantel-Cox test (**K**), or 2-way ANOVA (**I** and **R**). *P < 0.05; **P < 0.01; ***P < 0.001.

Analysis of patient data across multiple cancer types revealed that patients with high DC *DYRK1A* expression (*DYRK1A*-DC^hi^) had significantly better overall survival than those with low DC *DYRK1A* expression (*DYRK1A*-DC^lo^) (Figure 1A), highlighting the potential clinical relevance of DC *DYRK1A* expression. To further support this, we observed that tumor-infiltrating DCs expressed substantially higher levels of DYRK1A than their counterparts in tumor-draining lymph nodes (Figure 1B). To explore the regulation of DYRK1A in DCs, we stimulated bone marrow–derived DCs (BMDCs) with various cues. TLR activation significantly upregulated both mRNA and protein levels of DYRK1A (Figure 1C and Supplemental Figure 1A; supplemental material available online with this article; https://doi.org/10.1172/JCI199108DS1). Beyond typical TLR signaling, growth factors, amino acids, and tumor-derived DNA also markedly increased DYRK1A protein expression (Figure 1, D–F), indicating that the TME may promote DYRK1A expression in tumor-infiltrating DCs. Intriguingly, activated CD8^+^ T cells and IFN-γ were also capable of inducing DYRK1A in DCs (Figure 1, G and H, and Supplemental Figure 1B). Collectively, these findings position DYRK1A as an essential node in DC activation, potentially integrating diverse signals from the TME, including tumor-derived DNA, nutrients, and T cell feedback, to orchestrate DC function.

To investigate the function of DYRK1A in DCs, we specifically deleted the DYRK1A-encoding gene *Dyrk1a* by crossing *Dyrk1a*-floxed mice with *Cd11c*-Cre mice, generating *Dyrk1a* DC-conditional knockout (referred to as *Dyrk1a*-DC-cKO) and wild-type mice (Supplemental Figure 1, C and D). Real-time quantitative reverse-transcription PCR (qRT-PCR) and immunoblot analysis confirmed that the *Dyrk1a* gene was deleted specifically in DCs but not in splenic CD4^+^ T cells (Supplemental Figure 1, E and F). We next examined whether *Dyrk1a* deficiency affects DC development or physiology. The *Dyrk1a*-DC-cKO and their littermates exhibited similar frequencies of CD11c^+^MHCII^+^ DCs and DC subpopulations, including cDC1, cDC2, and plasmacytoid DCs, in various lymphoid tissues such as spleen, inguinal lymph nodes, and BM (Supplemental Figure 2, A–D). DYRK1A deficiency did not alter the percentages of migratory or resident DCs in draining lymph nodes (Supplemental Figure 2E) nor the expression of MHC molecules or maturation markers on splenic or lymph node DCs (Supplemental Figure 2F). It also did not appreciably influence the T cell population and homeostasis (Supplemental Figure 2, G and H). These results suggest that the absence of DYRK1A in DCs had no noticeable effect on their development and homeostasis.

To examine the role of DYRK1A in antitumor immunity, age- and sex-matched wild-type and *Dyrk1a*-DC-cKO littermates were challenged with B16-F10 melanoma cells. *Dyrk1a*-DC-cKO mice exhibited increased tumor burden and premature lethality (Figure 1, I–K). These mice also showed reduced frequencies and absolute numbers of CD4^+^ and CD8^+^ tumor-infiltrating lymphocytes (Figure 1L), as well as decreased CD8^+^ T cell proliferation in tumors (Figure 1M). Tumor-infiltrating CD8^+^ T cells from *Dyrk1a*-DC-cKO mice had higher programmed cell death 1 (PD-1) and T cell immunoglobulin mucin receptor 3 (TIM-3) expression (Figure 1N), indicating an exhausted phenotype. Moreover, DYRK1A ablation in DCs impaired effector T cell function, with reduced IFN-γ–, TNF-α–, and granzyme B–producing CD8^+^ T cells and fewer IFN-γ– and TNF-α–producing CD4^+^ T cells (Figure 1, O and P). To investigate whether DYRK1A ablation in DCs affects antigen-specific CD8^+^ T cell responses, we challenged *Dyrk1a*-DC-cKO and their littermates with B16-GP33 tumor cells and examined GP33 tetramer^+^CD8^+^ T cells. Notably, GP33 tetramer^+^CD8^+^ T cells were remarkably diminished in *Dyrk1a*-DC-cKO mice (Figure 1Q). These data illustrate that DYRK1A deficiency in DCs significantly attenuated antigen-specific CD8^+^ T cell responses. Analysis of draining lymph nodes further revealed impaired T cell function, with decreased numbers of CD44^+^ and cytokine-producing CD8^+^ and CD4^+^ T cells (Supplemental Figure 3). These phenotypes were not tumor model specific, as similar results were observed in the MC38 colon cancer model (Figure 1, R and S, and Supplemental Figure 4). Collectively, these data suggest that DYRK1A in DCs facilitates antitumor immunity by regulating the proliferation and function of tumor-infiltrating effector T cells.

### DYRK1A deficiency attenuates the maturation, antigen processing, and presentation of DCs.

**Figure 2:**
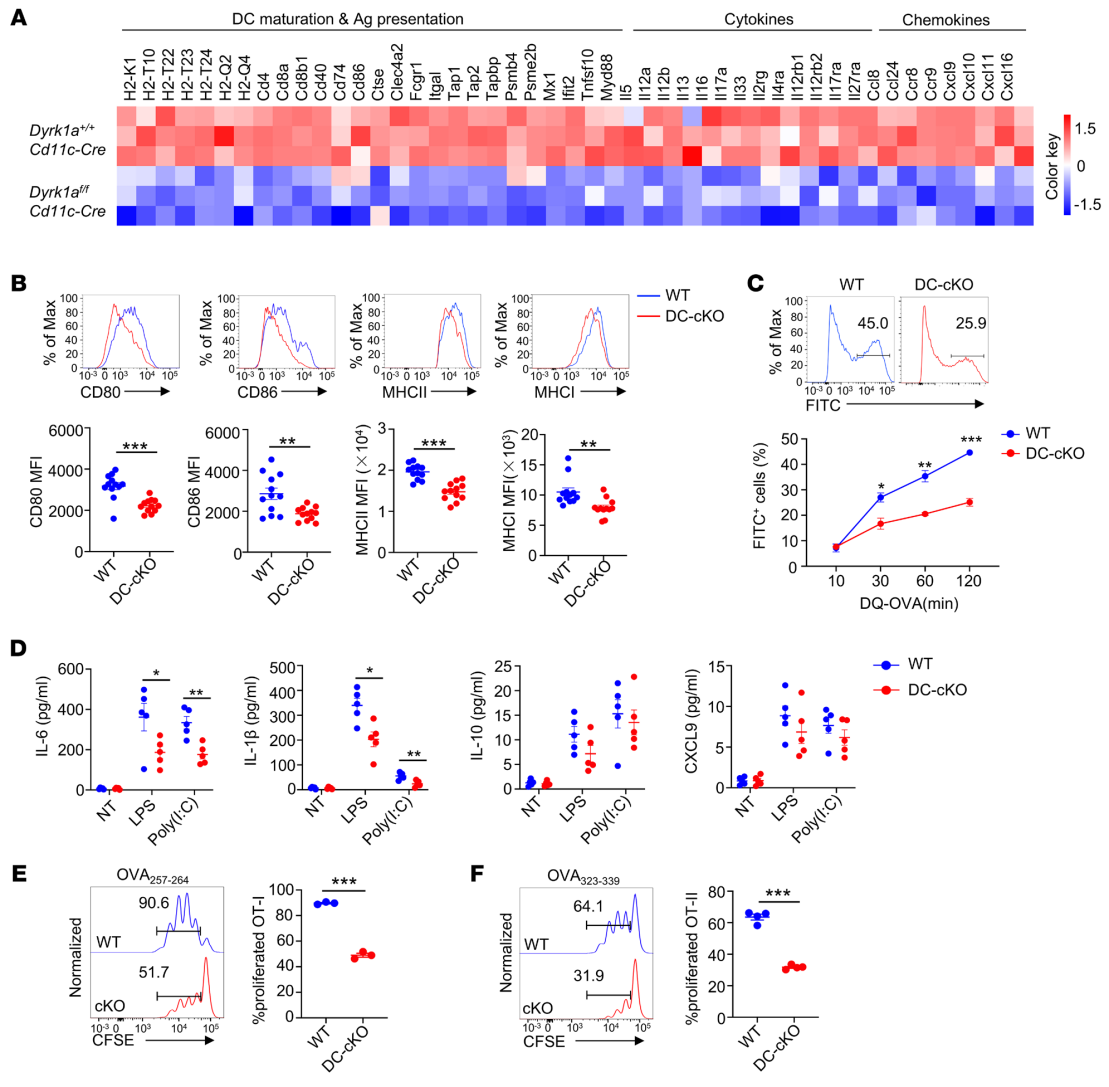
DYRK1A regulates the antigen processing, maturation, and cytokine expression of DCs. (**A**) RNA-sequencing analysis of MHCII^+^CD11c^+^ DCs freshly isolated from tumors of tumor-bearing mice injected s.c. with B16-F10, showing a heatmap of genes related to DC maturation, antigen presentation, cytokines, and chemokines. (**B**) Flow cytometric analysis of CD80, CD86, MHCI, and MHCII levels of tumor-infiltrating DCs (*N* = 12 mice/group). (**C**) WT and *Dyrk1a*-deficient DCs were incubated with DQ-OVA for the indicated time points, and the percentage of FITC^+^ cells was monitored by flow cytometry (*N* = 4 mice/group). (**D**) ELISA analysis of IL-6, IL-1β, IL-10, and CXCL9 cytokine levels in supernatants from wild-type and *Dyrk1a*-deficient BMDCs, either nontreated (NT) or stimulated with LPS or Poly(I:C) for 24 hours (*N* = 5 mice/group). (**E** and **F**) Flow cytometric analysis to measure the proliferation of CFSE-labeled OT-I and OT-II T cells incubated with OVA-pulsed WT and *Dyrk1a*-DC-cKO DCs. Data are representative of 3 independent experiments. Summary data are shown as the mean ± SEM. *P* values were determined using a 2-tailed unpaired Student’s *t* test. *P < 0.05; **P < 0.01; ***P < 0.001.

To dissect the function of DYRK1A in regulating tumor-infiltrating DCs, we analyzed the CD11c^+^MHCII^+^ DC population in tumors. DYRK1A deficiency did not affect the cell population or absolute cell numbers of tumor-infiltrating DCs, cDC1s, and cDC2s (Supplemental Figure 5). To understand how DYRK1A regulates DC antitumor functions, we performed RNA sequencing on freshly isolated tumoral DCs from tumor-bearing control and *Dyrk1a*-DC-cKO mice. Intriguingly, the expression levels of a large subset of genes related to DC maturation, antigen processing, and presentation, which contribute to the generation of antitumor immune responses (3, 15), were downregulated in tumoral DCs from *Dyrk1a*-DC-cKO mice (Figure 2A). Consistent with this, MHC and costimulatory molecule expression was drastically decreased on DCs from tumors and draining lymph nodes of *Dyrk1a*-DC-cKO mice (Figure 2B and Supplemental Figure 6), which was confirmed in *Dyrk1a*-deficient BMDCs in vitro (Supplemental Figure 7A). Consistent with the RNA-sequencing data, the antigen-processing ability of DCs was substantially abrogated in *Dyrk1a-*deficient cells (Figure 2C), though antigen uptake and migration remained normal (Supplemental Figure 7, B and C). Our RNA-sequencing data also suggested that DYRK1A deficiency inhibited the expression of several cytokines and chemokines (Figure 2A). Further ELISA analysis showed that IL-1β and IL-6 protein levels were significantly reduced in *Dyrk1a*-deficient DCs, whereas IL-10 and CXCL9 levels remained comparable between wild-type and *Dyrk1a*-deficient DCs (Figure 2D). We next examined T cell priming using an in vitro model in which OT-I or OT-II T cells were activated by DCs pulsed with specific peptides, chicken OVA_257–264_ or OVA_323–339_. *Dyrk1a*-deficient DCs substantially reduced OT-I and OT-II T cell proliferation as measured by CFSE dilution (Figure 2, E and F). Collectively, these data indicate that DYRK1A promotes DC maturation, antigen processing, and presentation, thereby eliciting effective antitumor immune responses.

### DYRK1A enhances the antitumor immune function of cDC1s by activating the mTORC1 pathway.

**Figure 3:**
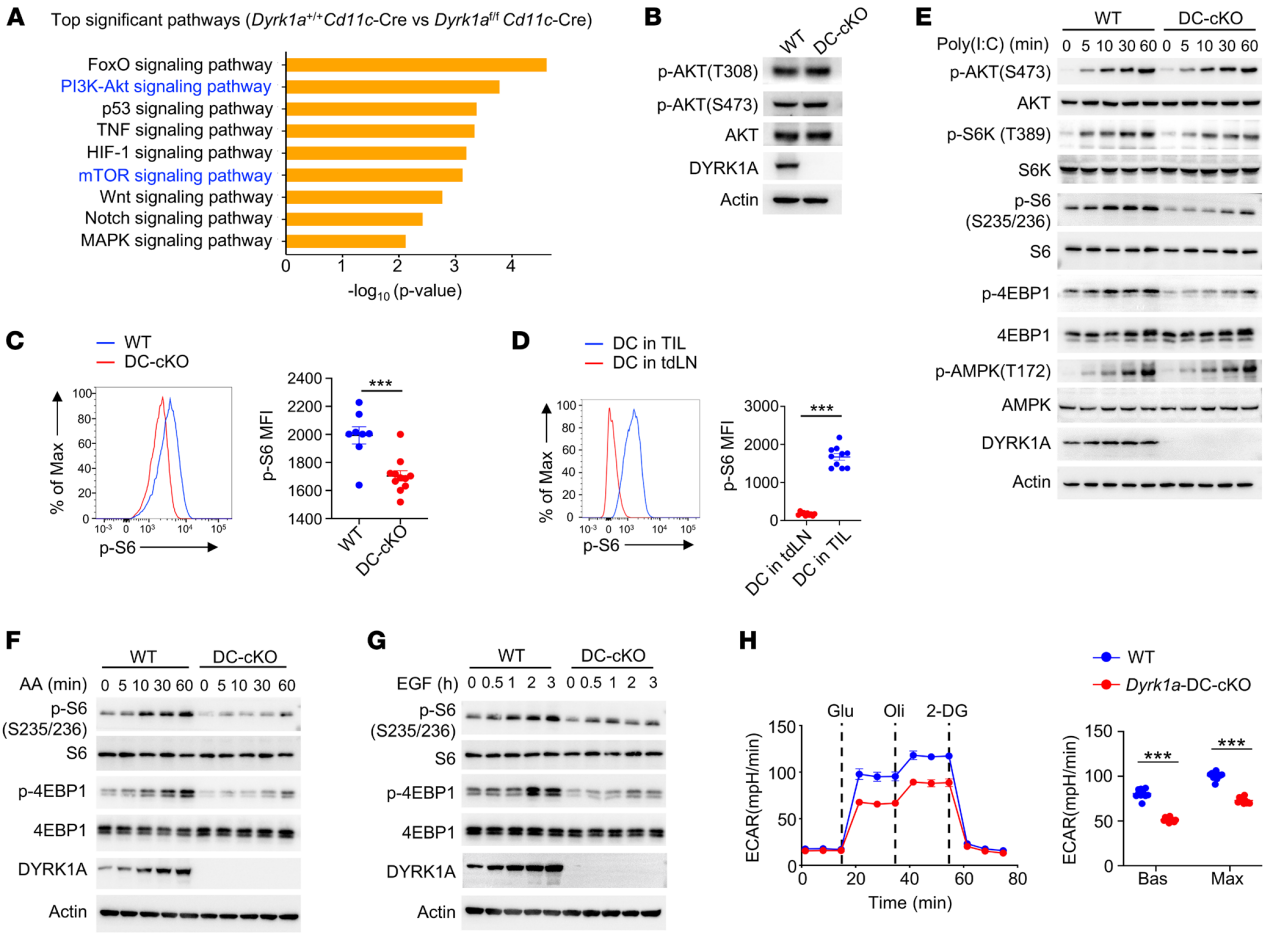
DYRK1A activates the mTORC1 signaling pathway in DCs. (**A**) Functional enrichment analysis of Kyoto Encyclopedia of Genes and Genomes pathways significantly changed in *Dyrk1a*-deficient DCs compared with wild-type DCs. (**B**) Immunoblot analysis of indicated proteins in whole-cell lysates of tumor DCs from wild-type and *Dyrk1a*-DC-cKO mice. (**C**) Flow cytometric analysis of p-S6 in tumor-infiltrating DCs freshly isolated from tumor-bearing wild-type and *Dyrk1a*-DC-cKO mice. Wild-type mice: *N* = 8; *Dyrk1a*-DC-cKO mice: *N* = 11. (**D**) Flow cytometric analysis of p-S6 in DCs isolated from tumor or tumor-draining lymph nodes (*N* = 10 mice/group). 4EBP1, eukaryotic translation initiation factor 4E-binding protein 1. (**E**) Immunoblot analysis of indicated proteins in whole-cell lysates of BMDCs stimulated with Poly(I:C) at the indicated time points. (**F** and **G**) Immunoblot analysis of the indicated proteins and phosphorylated (p-) proteins in whole-cell lysates of BMDCs stimulated with amino acids (**F**) and EGF (**G**). (**H**) Extracellular acidification rate (ECAR) of DCs stimulated with Poly(I:C) for 4 hours under basal conditions (Bas) or at maximum (Max) with the addition of glucose (Glu), oligomycin (Oli), and 2-deoxy-d-glucose (2-DG). Data are representative of 3 independent experiments. Summary data are shown as the mean ± SEM. *P* values were determined using a 2-tailed unpaired Student’s *t* test. ***P < 0.001.

Since DYRK1A promotes DC maturation and antigen presentation, we next sought to dissect the molecular mechanisms by which DYRK1A governs DC function. To this end, we performed functional pathway enrichment analysis of RNA-sequencing data and found that DYRK1A significantly affected the mTORC1 and PI3K-Akt signaling pathways in tumoral DCs (Figure 3A). Interestingly, immunoblotting analysis revealed that DYRK1A ablation did not affect the PI3K-Akt pathway (Figure 3B) but remarkably diminished the phosphorylation of ribosomal protein S6 (p-S6) in tumor-infiltrating DCs (Figure 3C). This p-S6 was notably elevated in tumor-infiltrating DCs compared with those from draining lymph nodes, mirroring the expression pattern of DYRK1A (Figure 3D). Moreover, stimulation of wild-type and *Dyrk1a*-deficient BMDCs with Poly(I:C) confirmed that DYRK1A deficiency does not affect the PI3K-Akt signaling but remarkably abrogates the mTORC1 pathway, as evidenced by decreased phosphorylation of ribosomal protein S6K1, ribosomal protein S6, and 4EBP1 in *Dyrk1a*-deficient DCs (Figure 3E). AMPK signaling negatively regulates the mTORC1 pathway, and a central step in this signaling axis is the phosphorylation of AMPK at T172, which triggers its catalytic activation (37). Here, we observed that DYRK1A deficiency did not influence AMPK activation in DCs (Figure 3E). Since growth factors and amino acids are well-characterized stimuli of mTORC1 signaling (38), we next examined the effect of DYRK1A deficiency on growth factor– or amino acid–mediated mTORC1 activation in DCs. Consistently, *Dyrk1a*-deficient DCs were hyporesponsive to both amino acid– and EGF-stimulated mTORC1 activation, as shown by the decreased phosphorylation levels of S6 and 4EBP1 (Figure 3, F and G). Furthermore, DYRK1A is required for mTORC1’s activity in DCs when stimulated by T cells or IFN-γ (Supplemental Figure 7, D and E). A previous study showed that DYRK1A mediated BAFF-induced noncanonical NF-κB activation in B cells (35). Nevertheless, DYRK1A was dispensable for canonical or noncanonical NF-κB activation in DCs (Supplemental Figure 7F), suggesting a cell type–specific function of DYRK1A.

Given that the mTORC1 signaling axis is crucial for metabolic activation (39), we examined the role of DYRK1A in regulating aerobic glycolysis by Seahorse extracellular flux analyses. *Dyrk1a*-deficient DCs displayed a drastic decrease in both baseline and maximum glycolytic rates, indicating a crucial role for DYRK1A in regulating DC glycolysis (Figure 3H). On the other hand, DYRK1A deficiency moderately decreased the baseline oxygen consumption rate (OCR) but did not affect stressed OCR (maximum respiratory capacity) (Supplemental Figure 7G). Together, these data suggest that DYRK1A promotes mTORC1 activation and glycolysis in DCs stimulated by TLR agonists, growth factors, and amino acids.

Given that mTORC1 signaling regulates DC activation in a subset-specific manner (40–42), we investigated the role of DYRK1A in modulating distinct DC subpopulations, including cDC1, cDC2, and Langerhans cells (LCs). It has been shown that mTORC1 signaling enhances MHC molecule expression, CD86 levels, and cross-presentation capacity in cDC1s (40). Nevertheless, studies have shown that *Raptor*-deficient DCs exhibited augmented antigen-specific CD8^+^ T cell responses during skin infection, correlating with heightened activation of LCs and a subset of EpCAM^+^ cDC1s (40). To explore this further, we analyzed LCs and EpCAM^+^ cDC1s in tumors and draining lymph nodes (LNs). Notably, *Dyrk1a*-deficient DC mice displayed a reduction in CD103^–^CD207^+^ LCs in both tumor and LN compartments (Supplemental Figure 8, A and C), aligning with prior reports that mTORC1 signaling sustains LC homeostasis (41). Intriguingly, DYRK1A deficiency did not alter IL-12 production by LCs (Supplemental Figure 8, B and D). Moreover, EpCAM^+^ cDC1s were nearly undetectable in tumors and draining LNs (Supplemental Figure 8, E and F), suggesting their presence may be context dependent, prominent in bacterial infection models but minimal in tumor settings.

**Figure 4:**
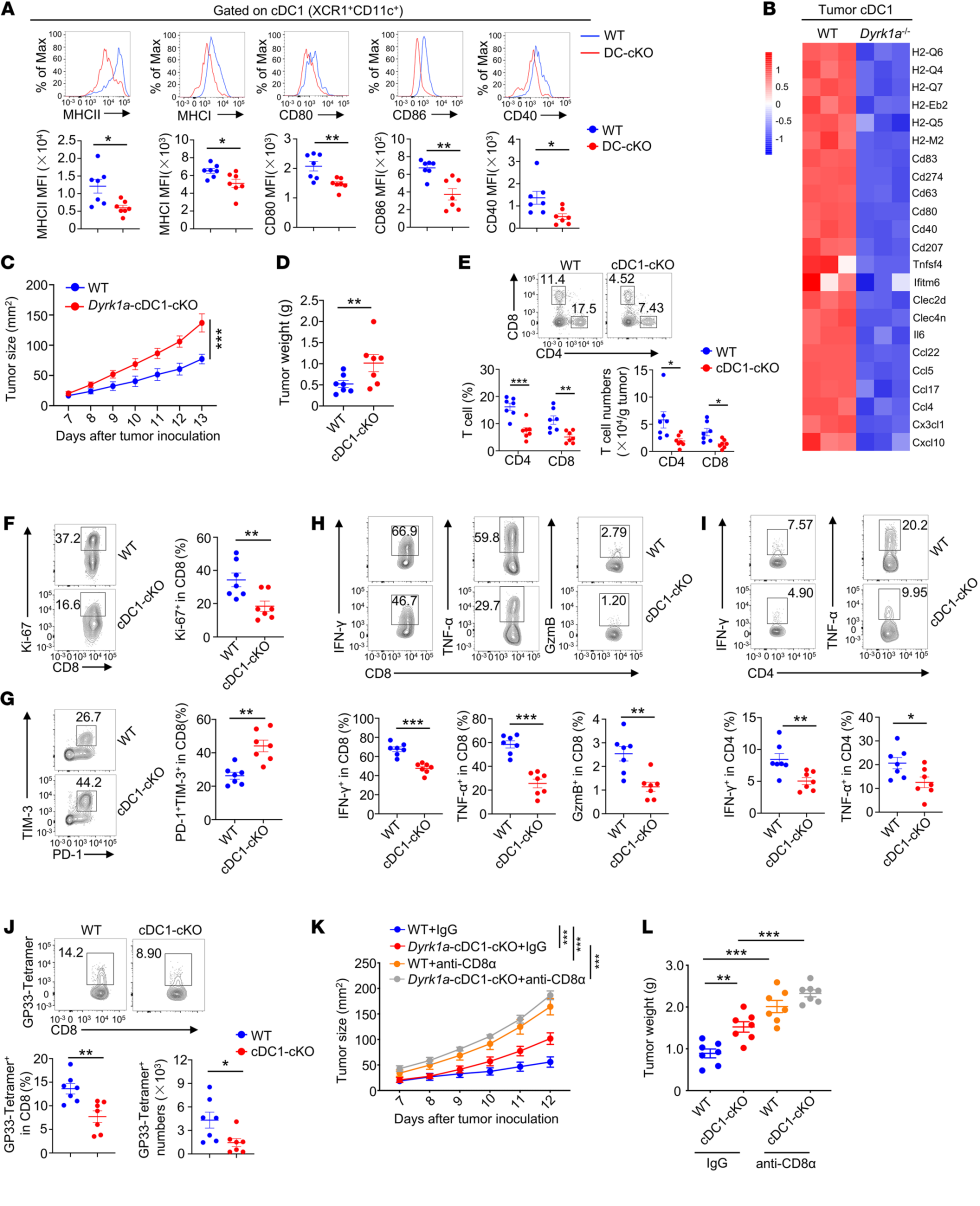
DYRK1A ablation in cDC1s dampens antitumor immunity. (**A**) Flow cytometric analysis of MHCI, MHCII, CD80, CD86, and CD40 levels of tumor-infiltrating cDC1s (XCR1^+^CD11c^+^). (**B**) RNA-sequencing analysis of tumor cDC1s purified from tumor-bearing mice injected s.c. with B16-F10, showing a heatmap of genes associated with cDC1 maturation, antigen presentation, cytokines, and chemokines. (**C** and **D**) Tumor growth curves (**C**) and tumor weight (**D**) of wild-type and *Dyrk1a*-cDC1-cKO mice s.c. injected with B16-GP33 melanoma cells (*N* = 7 mice/group). (**E**) Flow cytometric analysis of the frequency and cell number of tumor-infiltrating CD4^+^ and CD8^+^ T cells (*N* = 7 mice/group). (**F** and **G**) Flow cytometric analysis of Ki-67 (**F**) and TIM-3 and PD-1 (**G**) levels of tumor-infiltrating CD8^+^ T cells. *N* = 7 mice per group. (**H** and **I**) Flow cytometric analysis of the frequency of IFN-γ–, TNF-α–, and granzyme B-producing CD8^+^ T (**H**) and CD4^+^ T cells (**I**) in the tumor of wild-type and *Dyrk1a*-cDC1-cKO mice (*N* = 7 mice/group). (**J**) Flow cytometric analysis of the frequency and cell number of GP33-tetramer^+^ CD8^+^ T cells in the tumors (*N* = 7 mice/group). (**K** and **L**) Wild-type and *Dyrk1a*-cDC1-cKO mice with B16-F10 melanoma were treated with anti-CD8α or IgG (100 μg/mouse) on days 0, 3, 6, and 10 after tumor inoculation. Tumor growth curves (**K**) and tumor weight (**L**) are shown (*N* = 7 mice/group). Data are representative of 3 independent experiments. Summary data are shown as the mean ± SEM. *P* values were determined using a 2-tailed unpaired Student’s *t* test (**A** and **D**–**J**), 1-way ANOVA (**L**), and 2-way ANOVA (**C** and **K**). *P < 0.05; **P < 0.01; ***P < 0.001.

To delineate DYRK1A’s subset-specific functions, we assessed MHC and costimulatory molecule expression on tumor-infiltrating cDC1s and cDC2s. Strikingly, DYRK1A ablation markedly diminished MHCI, MHCII, CD86, CD80, and CD40 levels in cDC1s (Figure 4A) while leaving cDC2s unaffected (Supplemental Figure 9A). RNA sequencing of tumor-derived cDC1s from wild-type and *Dyrk1a*-DC-cKO mice further revealed downregulation of genes linked to cDC1 maturation, antigen presentation, and chemokine signaling in the absence of DYRK1A (Figure 4B). These data underscore a selective role for the DYRK1A/mTORC1 axis in orchestrating cDC1 maturation and activation, with minimal impact on cDC2s.

To investigate the role of DYRK1A in cDC1s, we generated cDC1-specific *Dyrk1a*-knockout mice (referred to as *Dyrk1a*-cDC1-cKO) by crossing *Dyrk1a*-floxed mice with *Xcr1*-Cre mice (Supplemental Figure 1, C and G). Successful deletion of *Dyrk1a* in cDC1s, but not in splenic CD4^+^ T cells, was confirmed by qRT-PCR and immunoblotting (Supplemental Figure 1, H and I). Upon B16-GP33 melanoma challenge, *Dyrk1a*-cDC1-cKO mice exhibited significantly increased tumor burden compared with wild-type littermates (Figure 4, C and D). Notably, both the frequencies and absolute numbers of tumor-infiltrating CD4^+^ and CD8^+^ T cells were reduced in *Dyrk1a*-cDC1-cKO mice (Figure 4E). CD8^+^ T cells from *Dyrk1a*-cDC1-cKO mice showed impaired proliferation (Figure 4F), elevated PD-1 and TIM-3 expression (Figure 4G), and functional defects, including decreased IFN-γ^+^, TNF-α^+^, and granzyme B^+^ CD8^+^ T cells, as well as reduced IFN-γ^+^ and TNF-α^+^ CD4^+^ T cells (Figure 4, H and I). Moreover, GP33-tetramer^+^CD8^+^ T cells were drastically diminished in *Dyrk1a*-cDC1-cKO mice (Figure 4J). To determine whether the antitumor immunity mediated by DYRK1A in cDC1s depends on CD8^+^ T cells, we administered anti-CD8α antibody to deplete this population. T cell depletion markedly accelerated tumor growth in both genotypes and abrogated the differences in tumor progression between wild-type and knockout mice (Figure 4, K and L), indicating that DYRK1A-enhanced antitumor immunity is CD8^+^ T cell dependent. The critical role of DYRK1A in cDC1s for antitumor immunity was further validated across multiple tumor models. The pro-tumorigenic effect of *Dyrk1a* ablation was consistently observed not only in the MB49 bladder cancer and MC38 colon cancer models (Supplemental Figures 10 and 11) but also in an orthotopic tumor setting, where cDC1-specific *Dyrk1a* deletion similarly exacerbated tumor progression and dampened antitumor immunity (Supplemental Figure 12). Collectively, our findings demonstrate that DYRK1A deficiency in cDC1s attenuates antigen-specific CD8^+^ T cell function, thereby compromising the antitumor immune response.

**Figure 5:**
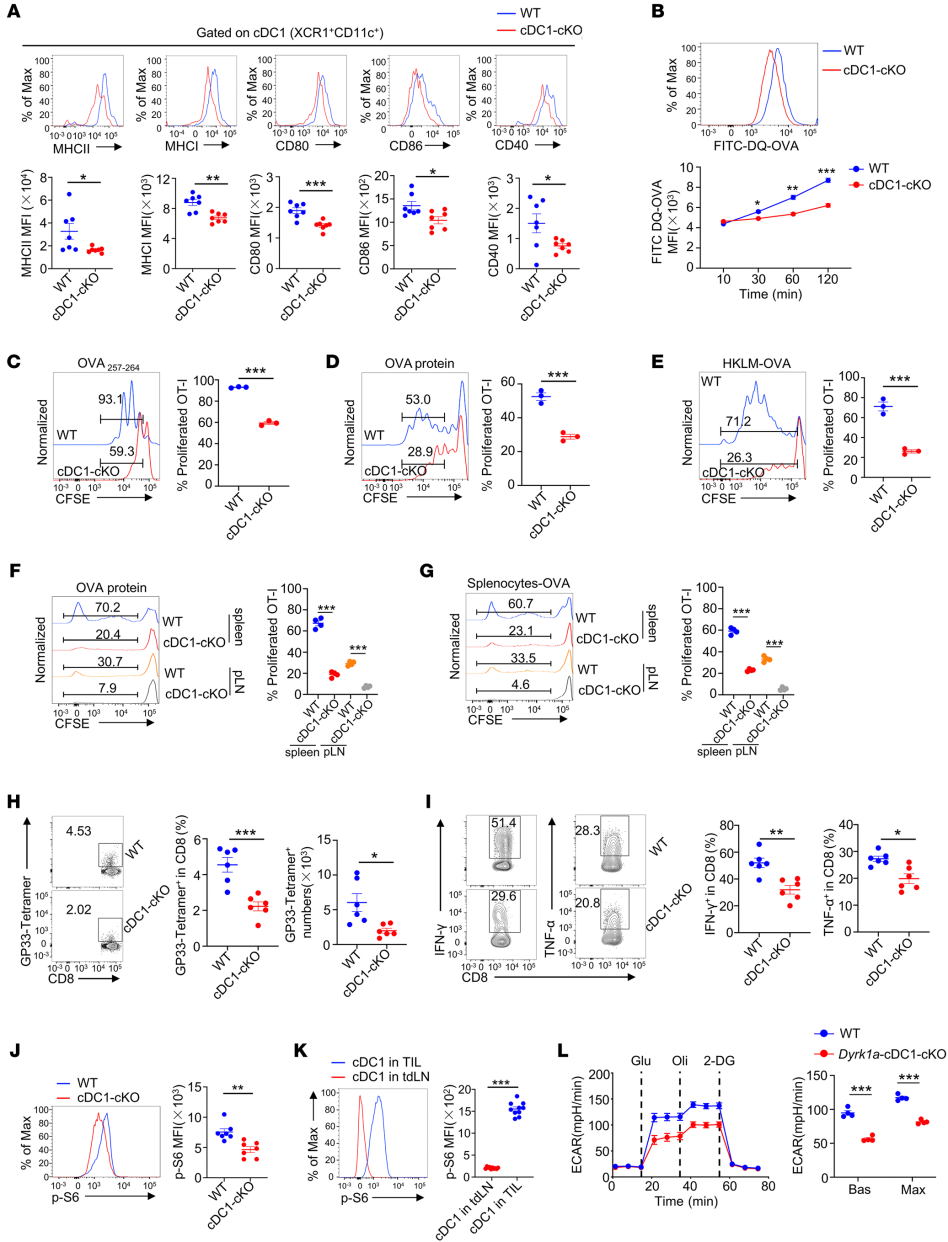
DYRK1A promotes activation and antigen presentation of cDC1s. (**A**) Flow cytometric analysis of MHCI, MHCII, CD80, CD86, and CD40 on tumor-infiltrating cDC1s from wild-type and *Dyrk1a*-cDC1-cKO mice (*N* = 7 mice/group). (**B**) Wild-type and *Dyrk1a*-deficient cDC1s were incubated with dye-quenched OVA (DQ-OVA), and the mean fluorescence intensity (MFI) of FITC DQ-OVA was examined at the indicated time points (*N* = 4/group). (**C**–**E**) Proliferation of CFSE-labeled OT-I T cells incubated with WT or *Dyrk1a*-DC-cKO cDC1s pulsed with OVA_257–264_ peptide (**C**), OVA protein (**D**), or heat-killed *Listeria* *monocytogenes* overexpressing OVA (HKLM-OVA) (**E**), measured by flow cytometry (*N* = 3/group). (**F** and **G**) CFSE-labeled OT-I T cells were adoptively transferred into WT or *Dyrk1a*-cDC1-cKO mice. After 24 hours, mice were immunized i.v. with OVA protein (**F**) or irradiated OVA-loaded *β2m*^–/–^ splenocytes (**G**). OT-I proliferation in spleen and lymph nodes was shown (*N* = 4 mice/group). (**H** and **I**) WT or *Dyrk1a*-deficient cDC1s were pulsed with B16-GP33 tumor lysates and Poly(I:C) for 12 hours, then transferred into naive mouse footpads. Seven days later, popliteal lymph nodes were harvested, and the frequency and number of GP33 tetramer^+^CD8^+^ T cells were assessed by flow cytometry (**H**). Lymphocytes were stimulated with GP33 peptide (8 hours) for intracellular staining of IFN-γ and TNF-α in CD8^+^ T cells (*N* = 6 mice/group) (**I**). (**J**) Flow cytometric analysis of p-S6 in tumor-infiltrating cDC1s from WT and *Dyrk1a*-cDC1-cKO mice (*N* = 7 mice/group). (**K**) Flow cytometric analysis of p-S6 in cDC1s from tumor or tumor-draining lymph nodes (*N* = 10 mice/group). (**L**) ECAR of cDC1s stimulated with Poly(I:C) for 4 hours under basal conditions (Bas) or at maximum (Max) by adding glucose (Glu), oligomycin (Oli), and 2-deoxy-d-glucose (2-DG) (*N* = 4/group). Data are representative of 3 independent experiments. Summary data are shown as the mean ± SEM. *P* values were determined using a 2-tailed unpaired Student’s *t* test. *P < 0.05; **P < 0.01; ***P < 0.001.

To investigate the role of DYRK1A in modulating tumor-infiltrating cDC1s, we assessed the CD11c^+^XCR1^+^ cDC1 population in tumors. DYRK1A deficiency did not alter cDC1 abundance (Supplemental Figure 13A) but markedly reduced their MHC and costimulatory molecule expression (Figure 5A). Notably, EpCAM^+^ cDC1s were scarcely detectable among tumor-infiltrating lymphocytes, with no differences between wild-type and *Dyrk1a*-cDC1-cKO groups (Supplemental Figure 13B). We then cultured bone marrow–derived cDC1s (BM-cDC1s) in vitro, achieving high purity as confirmed by flow cytometry (Supplemental Figure 13C). Strikingly, DYRK1A deficiency severely impaired antigen-processing capacity (Figure 5B) and reduced IL-1β and IL-6 production in cDC1s (Supplemental Figure 13D). We further investigated whether DYRK1A was involved in regulating the T cell–priming capacity of cDC1s. We employed an in vitro system in which OT-I T cells were activated by cDC1s pulsed with the OVA_257–264_ peptide, full-length OVA protein, or heat-killed *Listeria monocytogenes* overexpressing OVA. *Dyrk1a-*deficient cDC1s induced significantly reduced OT-I T cell proliferation compared with wild-type controls (Figure 5, C–E). In contrast, DYRK1A deficiency did not impair cDC2-mediated T cell priming (Supplemental Figure 9B). We next investigated whether this defect extends to antigen presentation in vivo. To this end, we administered OVA protein or OVA-loaded *β2m*^–/–^ splenocytes intravenously into wild-type and *Dyrk1a*-cDC1-cKO mice, followed by adoptive transfer of OT-1 T cells. Consistent with in vitro findings, *Dyrk1a*-deficient cDC1s elicited substantially weaker T cell proliferation in vivo than their wild-type counterparts (Figure 5, F and G). Furthermore, in an in vivo priming assay using B16-GP33 lysate-pulsed cDC1s transferred into naive mice, DYRK1A deficiency markedly reduced the frequency and number of GP33 tetramer^+^CD8^+^ T cells in draining LNs (Figure 5H) and compromised effector cytokine production upon GP33 restimulation (Figure 5I). These results demonstrate that DYRK1A is essential for cDC1-mediated antigen presentation and T cell priming, thereby driving effective antitumor immunity.

cDC1s present exogenous antigens on MHCI molecules and prime CD8^+^ T cells, a process that requires precise regulation to prevent excessive lysosomal degradation of antigenic peptides (43, 44). Partial antigen degradation allows ingested antigens to escape phagocytic pathways and enter the cytosol for proteasomal processing, thereby supporting cross-presentation (45). To investigate whether DYRK1A regulates proteasome-mediated antigen degradation, we electroporated DQ-OVA and Alexa Fluor 647–OVA into the cytosol of wild-type and *Dyrk1a*-deficient cDC1s. DYRK1A ablation impaired proteasomal degradation of exogenous antigens (Supplemental Figure 14, A and B), indicating a compromised capacity for antigen presentation. Using the lysosomal inhibitor Bafilomycin A1 (BafA1) to isolate proteasome-mediated degradation, we confirmed impaired antigen degradation in *Dyrk1a*-deficient cDC1s (Supplemental Figure 14, C and D). We next asked whether DYRK1A also influences lysosome-mediated antigen degradation. Notably, *Dyrk1a* deficiency enhanced antigen degradation in the phagolysosome (Supplemental Figure 14E), further attenuating the antigen-presenting capacity and immunogenicity of cDC1s. Consistently, lysosome-mediated antigen degradation was markedly increased in *Dyrk1a*-deficient cDC1s relative to wild-type controls in the presence of the proteasome inhibitor MG-132 (Supplemental Figure 14F). Given that mTORC1 inhibition triggers MiT/TFE-dependent lysosome biogenesis (46, 47), we assessed whether DYRK1A affects lysosomal generation or acidification. Indeed, *Dyrk1a*-deficient cDC1s exhibited enhanced lysosome biogenesis (Supplemental Figure 14G). To evaluate phagosomal acidification, we incubated cDC1s with OVA-coated latex beads conjugated to either FITC (pH-sensitive) or Alexa Fluor 647 (pH-insensitive) and analyzed them by flow cytometry over time. The phagosomes in *Dyrk1a*-deficient cDC1s were more acidic than those in wild-type cells (Supplemental Figure 14H). Consistent with impaired cross-presentation, bulk RNA sequencing revealed downregulation of the gene set related to “antigen processing and presentation of peptide antigen via MHC class I” in *Dyrk1a*-deficient tumor-infiltrating cDC1s (Supplemental Figure 14I). In summary, *Dyrk1a* deficiency promotes lysosomal antigen degradation by enhancing lysosomal biogenesis and phagosomal acidification, while reducing proteasomal degradation in cDC1s, thereby collectively compromising antigen presentation and the immunogenicity of cDC1s.

Our previous data showed notable upregulation of DYRK1A in DCs following stimulation with TLR ligands, growth factors, and amino acids (Figure 1, C–E), corroborated by elevated DYRK1A protein levels in stimulated cDC1s (Supplemental Figure 15, A–C). Consistent with previous results, DYRK1A deficiency substantially impaired mTORC1 signaling, as shown by reduced p-S6K1, p-S6, and p-4EBP1 in *Dyrk1a*-deficient cDC1s (Supplemental Figure 15, D and E). Flow cytometric analysis confirmed reduced p-S6 in tumor-infiltrating cDC1s from *Dyrk1a*-cDC1-cKO mice (Figure 5J). Notably, tumor-infiltrating cDC1s exhibited higher p-S6 levels than their counterparts in draining LNs (Figure 5K). Moreover, DYRK1A-deficient cDC1s exhibited impaired glycolytic capacity, with dramatically reduced baseline and maximum glycolytic rates (Figure 5L), indicating a crucial role for DYRK1A in regulating cDC1 glycolysis.

Next, we examined whether the mTORC1 pathway modulates cDC1 maturation, antigen processing, and antigen presentation. To this end, we used rapamycin to inhibit the mTORC1 pathway in cDC1s and observed that it markedly reduced levels of p-S6K, p-S6, and p-4EBP1 (Supplemental Figure 16A). Compared with control, rapamycin-treated cDC1s significantly downregulated the expression of MHC and costimulatory molecules, as well as antigen-processing capabilities (Supplemental Figure 16, B and C). In line with this, rapamycin-treated cDC1s markedly impaired the proliferative capacity of OT-I T cells compared with the control (Supplemental Figure 16D). These data demonstrate that the mTORC1 pathway positively regulates cDC1 maturation, antigen processing, and antigen presentation. Importantly, pharmacological mTORC1 inhibition phenocopies the functional defects observed in DYRK1A-deficient cDC1s, supporting a model in which DYRK1A potentiates cDC1 activity through mTORC1 activation.

In addition to modulating antigen presentation, DYRK1A regulates costimulatory molecule expression and cytokine production in cDC1s. Given the well-established role of interferon regulatory factor (IRF) transcription factors in DC activation and maturation (48), we examined *Irf* transcript levels in tumor-infiltrating wild-type versus *Dyrk1a*-deficient cDC1s. RNA sequencing revealed decreased expression of *Irf4*, *Irf1*, *Irf5*, *Irf8*, and *Irf3* in *Dyrk1a*-deficient cDC1s (Supplemental Figure 17A). This downregulation of *Irf1* and *Irf5* mRNA was consistently validated by qPCR (Supplemental Figure 17B). Immunoblot analysis confirmed reduced IRF1 and IRF5 protein levels in Poly(I:C)-stimulated *Dyrk1a*-deficient cDC1s (Supplemental Figure 17C). Notably, pharmacological inhibition of mTORC1 with rapamycin in wild-type cDC1s recapitulated this phenotype, lowering IRF1 and IRF5 protein levels, indicating that their maintenance depends on the DYRK1A/mTORC1 axis (Supplemental Figure 17D). Consistently, downstream targets of IRF1 and IRF5, including *Il6*, *Il12b*, *Tnf*, and *Isg15*, were also significantly reduced in *Dyrk1a*-deficient cDC1s (Supplemental Figure 17E). To determine whether DYRK1A and mTORC1 support cDC1 function through IRF1, we performed a rescue experiment by overexpressing IRF1 in *Dyrk1a*-deficient cDC1s and then evaluated costimulatory molecule expression and cytokine production after stimulation (Supplemental Figure 17, F–H). IRF1 overexpression restored both costimulatory molecule levels and cytokine secretion in *Dyrk1a*-deficient cDC1s to near-wild-type levels (Supplemental Figure 17, G and H). Together, these results establish a causal link by which the DYRK1A/mTORC1 axis promotes cDC1 effector function, primarily via IRF1.

### DYRK1A interacts with TSC1/2 and destabilizes the TSC complex.

**Figure 6:**
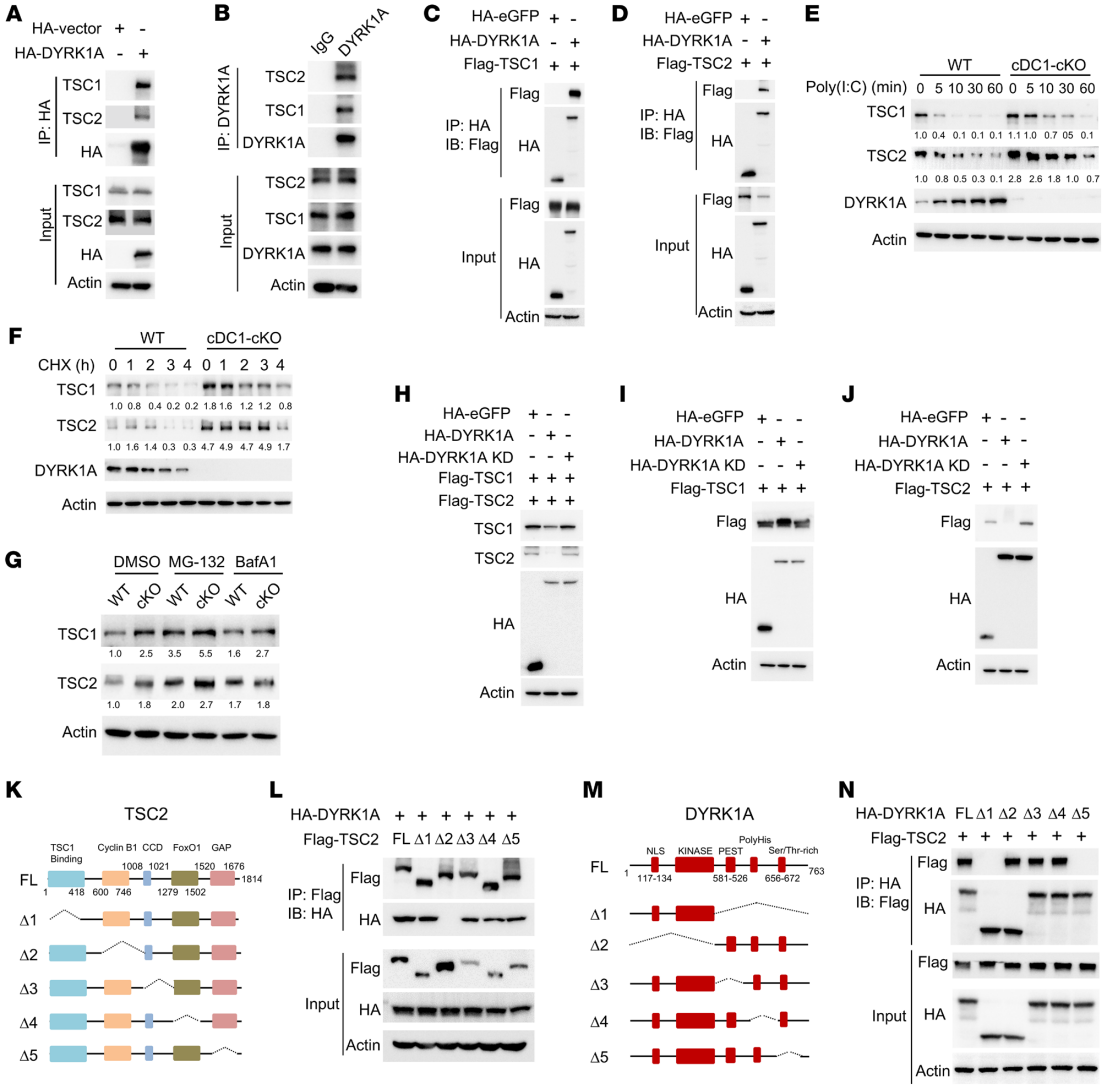
DYRK1A interacts with TSC1/2 and regulates its stability. (**A**) Co-IP analysis of DYRK1A interaction with TSC1 or TSC2 using whole-cell lysates of HEK293 cells transfected with the indicated expression vectors. (**B**) Co-IP assays to analyze the interaction of endogenous DYRK1A with TSC1 or TSC2 using whole-cell lysates of DC2.4 cells. (**C** and **D**) Co-IP analysis of DYRK1A interaction with TSC1 (**C**) or TSC2 (**D**) using whole-cell lysates of HEK293 cells transfected with the indicated expression vectors. (**E**) Immunoblot analysis of indicated proteins in whole-cell lysates of wild-type and *Dyrk1a*-deficient BM-cDC1s stimulated with Poly(I:C) at the indicated time points. (**F**) Immunoblot analysis of the indicated proteins in whole-cell lysates of wild-type and *Dyrk1a*-deficient BM-cDC1s that were treated with cycloheximide (CHX) for the indicated time points. (**G**) Immunoblot analysis of the indicated proteins in wild-type and *Dyrk1a*-deficient BM-cDC1s stimulated for 1 hour with Poly(I:C), followed by incubation with the indicated agents for 2 hours. (**H**–**J**) Immunoblot analysis of indicated proteins in whole-cell lysates of HEK293 cells transfected with the indicated vectors. (**K**) Schematic summary of TSC2 and its truncation mutants. (**L**) Co-IP analysis of DYRK1A interaction with TSC2 mutants using whole-cell lysates of HEK293 cells transfected with the indicated expression vectors. (**M**) Schematic summary of DYRK1A and its truncation mutants. (**N**) Co-IP analysis of TSC2 interaction with DYRK1A mutants using whole-cell lysates of HEK293 cells transfected with the indicated expression vectors. Data are representative of 3 independent experiments. KD, kinase dead; FL, full-length; CCD, coiled-coil domain; GAP, GTPase-activating protein domain; NLS, nuclear localization signal; PEST, PEST sequence.

To identify the mechanism by which DYRK1A affects mTORC1 signaling, we performed a co-immunoprecipitation assay with exogenous HA-tagged DYRK1A, followed by mass spectrometry. This identified several known DYRK1A binding proteins (35, 49), including TRAF3, TRAF2, DCAF7, CREBBP, and SIPA1L1 (Supplemental Figure 18, A and B). Notably, DYRK1A also interacted with TSC1 and TSC2, which inhibit mTORC1 activation by inactivating Ras homolog enriched in brain (50). These results prompted us to investigate the potential role of TSC1 and TSC2 in DYRK1A-mediated mTORC1 activation. We validated the interaction of DYRK1A with the TSC1 and TSC2 proteins using a targeted co-immunoprecipitation assay (Figure 6A). The interactions between TSC1, TSC2, and DYRK1A were also readily detected under endogenous conditions in DCs (Figure 6B). Since TSC1 and TSC2 form a complex, we examined whether DYRK1A binds to TSC1 or TSC2 individually. Indeed, the co-immunoprecipitation assay revealed that DYRK1A can bind to TSC1 or TSC2 separately (Figure 6, C and D, and Supplemental Figure 18, C and D).

While we were surveying the consequences of interrogating DYRK1A on mTORC1 activity, we noticed substantially increased levels of TSC1 and TSC2 in *Dyrk1a*-deficient cDC1s under both untreated and Poly(I:C)-stimulated conditions (Figure 6E). Cycloheximide chase assays indicated that TSC1 and TSC2 were more stable in the *Dyrk1a*-deficient cDC1s compared with control cells (Figure 6F). Additionally, incubating cDC1s with a proteasome inhibitor (MG-132) or a lysosomal inhibitor (BafA1) partially blocked the degradation of TSC1 or TSC2 (Figure 6G). These data suggest that DYRK1A mediates proteasomal and lysosomal degradation of TSC proteins.

To investigate whether the stability of TSC1 or TSC2 is dependent on the kinase activity of DYRK1A, we transfected both TSC1 and TSC2 with wild-type or kinase-dead DYRK1A (K188R) (35). Wild-type DYRK1A accelerated the degradation of both TSC1 and TSC2 in a kinase activity–dependent manner (Figure 6H). However, when TSC1 or TSC2 was transfected alone, DYRK1A specifically promoted TSC2 degradation but not TSC1 (Figure 6, I and J). Given that TSC1 and TSC2 form a complex, DYRK1A-mediated TSC2 degradation may destabilize the complex, leading to TSC1 degradation in vivo. Nonetheless, the mechanism by which TSC2 phosphorylation regulates the stability of the TSC complex remains to be investigated. To pinpoint the structural domain for DYRK1A/TSC2 interaction, we generated various truncated forms of DYRK1A and TSC2 for co-immunoprecipitation assays, revealing that the cyclin B1 binding domain of TSC2 and the Ser/Thr-rich domain of DYRK1A mediate their interaction (Figure 6, K–N). Collectively, these results indicate that DYRK1A binding and phosphorylation of TSC2 regulate the TSC complex stability and subsequent mTORC1 activity.

The lysosomal surface is the primary site for mTORC1 activation, where Rag GTPases (including RagA/B and RagC/D) recruit mTORC1 by binding Raptor and transducing amino acid signals (51, 52). To determine whether DYRK1A regulates the Rag GTPase–dependent activation pathway, we performed co-immunoprecipitation assays in control and *Dyrk1a*-knockdown HEK293 cells transfected with Flag-Raptor. *Dyrk1a* knockdown did not affect Raptor-RagA or Raptor-RagC interactions (Supplemental Figure 19A), indicating that DYRK1A is not essential for Rag GTPase–mediated mTORC1 activation. We also assessed whether DYRK1A influences the lysosomal localization of mTOR. In *Dyrk1a*-deficient cDC1s and *Dyrk1a*-knockdown HEK293 cells, the subcellular distribution of mTOR to lysosomes remained unchanged compared to their respective controls (Supplemental Figure 19, B and C). These results illustrate that DYRK1A deficiency does not impair the lysosomal localization of mTOR, suggesting that DYRK1A regulates mTORC1 activity independently of the Rag GTPase pathway.

### DYRK1A phosphorylates TSC2 at serine 540 and modulates its stability.

**Figure 7:**
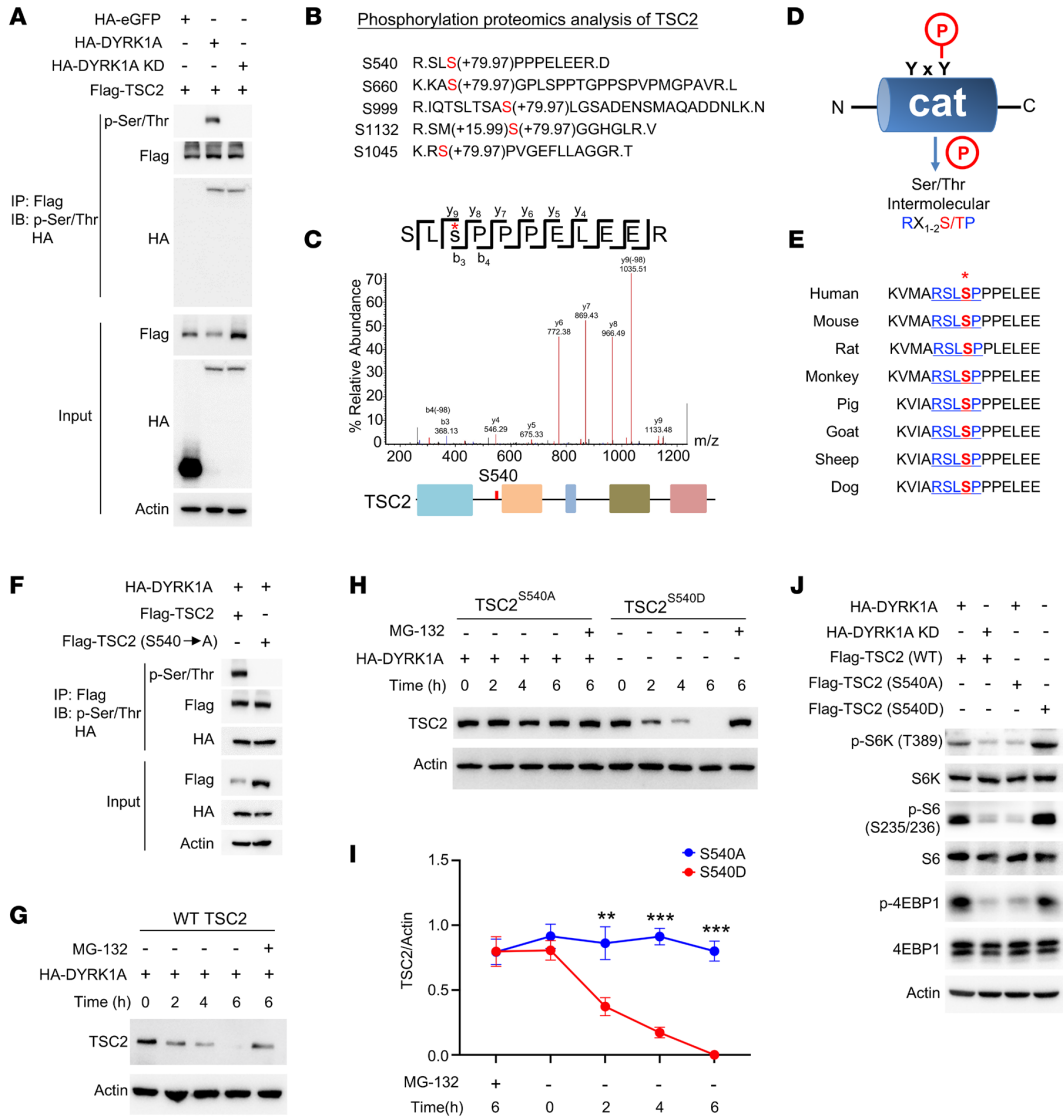
DYRK1A facilitates TSC2 degradation through the phosphorylation of TSC2 at S540. (**A**) Co-IP analysis of TSC2 followed by immunoblot analysis of p-Ser/Thr and indicated proteins using whole-cell lysates of HEK293 cells transfected with the indicated expression vectors. (**B**) Mass spectrometric analysis of potential phosphorylation sites of TSC2 by DYRK1A. (**C**) The panel depicts the phosphorylation site S540 in TSC2. Asterisk at the top, serine 540 residue that was found phosphorylated, determined by mass spectrometric analysis. (**D**) Schematic summary of conserved phosphorylation domains of DYRK1A. (**E**) Amino acid sequences around the serine 540 residue in TSC2 across different species. Asterisk at the top, serine residue that is conserved across species. (**F**) HEK293 cells were transfected with HA-DYRK1A and Flag-WT TSC2 or S540A mutant. Proteins precipitated by anti-Flag were blotted with anti–p-Ser/Thr followed by anti-TSC2. (**G**–**I**) Immunoblot analysis of TSC2 using whole-cell lysates of HEK293 cells transfected with WT TSC2 (**G**), S540A (**H**), S540D (**H**), and DYRK1A at the indicated time points after CHX treatment with or without MG-132. (**I**) Summary graph of quantified TSC2 protein bands of **H** (*N* = 4/group). Note that we transfected an excessive amount of cDNA for the S540D mutant, such that its initial expression level is close to that of the S540A mutant, to ease a direct comparison. (**J**) Effect of WT and mutant TSC2 on DYRK1A-induced S6K/S6/4EBP1 phosphorylation. Immunoblot analysis was performed on whole-cell lysates of HEK293 cells 48 hours after transfection with the indicated expression vectors to detect the specified proteins. Data are representative of 3 independent experiments. *P* values were determined using a 2-tailed unpaired Student’s *t* test (**I**). **P < 0.01; ***P < 0.001.

We next asked whether DYRK1A phosphorylates TSC2 and, if so, what the associated physiological function is. Ectopic expression of wild-type, but not kinase-dead, DYRK1A increased the p-Ser/Thr level of TSC2 (Figure 7A). To identify specific DYRK1A phosphorylation sites on TSC2, we performed phosphoproteomic analysis in the presence or absence of exogenous DYRK1A and detected 5 modification sites: S540, S660, S999, S1132, and S1045 (Figure 7B). Among these, S540 resides within a motif that closely matches the optimal DYRK1A substrate consensus (Figure 7, C and D) and is conserved across vertebrates (Figure 7E). To further investigate these sites, we introduced serine-to-alanine (nonphosphorylatable) point mutations. Only the S540A mutation substantially abrogated the p-Ser/Thr signal, indicating that DYRK1A specifically phosphorylates TSC2 at S540 (Figure 7F). We next examined whether S540 phosphorylation affects TSC2 stability. Exogenous DYRK1A promoted TSC2 degradation, which was partially blocked by MG-132 (Figure 7G). On the other hand, DYRK1A failed to degrade the S540A mutant (Figure 7, H and I). Conversely, a phosphomimetic S540D mutant underwent spontaneous degradation, which was largely rescued by MG-132 (Figure 7, H and I). Functionally, ectopic DYRK1A enhanced mTORC1 signaling, whereas TSC2 S540A substantially diminished it; in contrast, TSC2 S540D markedly promoted mTORC1 activation (Figure 7J). Previous studies reported that DYRK1A phosphorylates TSC2 at T1462 to modulate mTORC1 signaling (49). Notably, in cDC1s, DYRK1A did not alter TSC2 phosphorylation at T1462, as evidenced by comparable p-TSC2 (T1462)/total TSC2 ratios (Supplemental Figure 20, A and B). These findings suggest that DYRK1A-mediated TSC2 phosphorylation may exhibit cell type specificity.

To investigate the functional impact of S540 phosphorylation in cDC1-mediated antitumor immunity, we reconstituted *Tsc2*^–/–^ cDC1s with wild-type, phosphomimetic (S540D), or phosphorylation-deficient (S540A) TSC2 plasmids (Supplemental Figure 21A). The S540D mutant exhibited a more pronounced reduction in protein levels compared with WT TSC2, whereas the S540A mutant showed increased stability (Supplemental Figure 21A). Upon stimulation, S540D-expressing cDC1s exhibited markedly elevated levels of MHC and costimulatory molecules compared with their WT counterparts, whereas S540A-expressing cells showed substantial reductions (Supplemental Figure 21B). Functionally, S540D cDC1s were remarkably more potent than WT cDC1s in priming OT-1 CD8^+^ T cells, whereas S540A cDC1s exhibited impaired priming capacity (Supplemental Figure 21C). We further evaluated the therapeutic impact of S540 phosphorylation in a cDC1-based therapy model. Strikingly, mice receiving S540D cDC1s exhibited superior tumor control and a more robust infiltration of effector T cells into tumors than those receiving WT cDC1s. By contrast, the S540A group showed attenuated antitumor efficacy and T cell response (Supplemental Figure 21, D and E). Consistent with in vitro findings, S540D cDC1s maintained higher surface expression of MHC and costimulatory molecules in vivo, while S540A cDC1s expressed lower levels compared with WT counterparts (Supplemental Figure 21F). At the functional level, tumor-infiltrating T cells from mice treated with S540D cDC1s produced significantly greater amounts of IFN-γ and TNF-α upon restimulation than those from the WT group (Supplemental Figure 21, G and H). Collectively, our results establish that DYRK1A-mediated phosphorylation of TSC2 at S540 is a critical positive regulator of cDC1 antitumor function.

To investigate the mechanism by which TSC2 phosphorylation regulates its stability, we performed ubiquitination assays. We found that the S540D mutant exhibited substantially increased ubiquitination compared with wild-type TSC2, while the S540A mutant showed dramatically reduced ubiquitination (Supplemental Figure 22A). Through mass spectrometry screening, we identified 6 potential TSC2-interacting E3 ubiquitin ligases (HUWE1, UBR5, RBP2, TPIPC, HECD1, and RBBP6), among which HUWE1 showed phosphorylation-dependent binding affinity to TSC2 in immunoprecipitation assays (Supplemental Figure 22B). Subsequent HUWE1 knockdown experiments demonstrated its essential role in mediating TSC2 ubiquitination (Supplemental Figure 22, C and D). Moreover, HUWE1 knockdown diminished K48-linked, rather than K63-linked, ubiquitination of TSC2 (Supplemental Figure 22, E and F). These data provide compelling evidence that DYRK1A-mediated phosphorylation at S540 enhances HUWE1 binding to TSC2, thereby promoting K48-linked ubiquitination and subsequent degradation of TSC2.

### Deletion of TSC2 in Dyrk1a-deficient cDC1 restores its antitumor immunity.

To confirm the functional relationship between TSC2 and DYRK1A, we genetically deleted *Tsc2* in *Dyrk1a*-deficient cDC1s and systematically assessed costimulatory molecule expression, antigen processing, and T cell–priming capacity. Compared with *Dyrk1a*-deficient cDC1s, *Tsc2* knockout significantly increased the expression of MHC and costimulatory molecules, including MHCI, MHCII, CD80, CD86, and CD40 (Supplemental Figure 23A). Moreover, TSC2 deletion restored the abilities of antigen processing and T cell priming in *Dyrk1a*-deficient cDC1s (Supplemental Figure 23, B and C).

**Figure 8:**
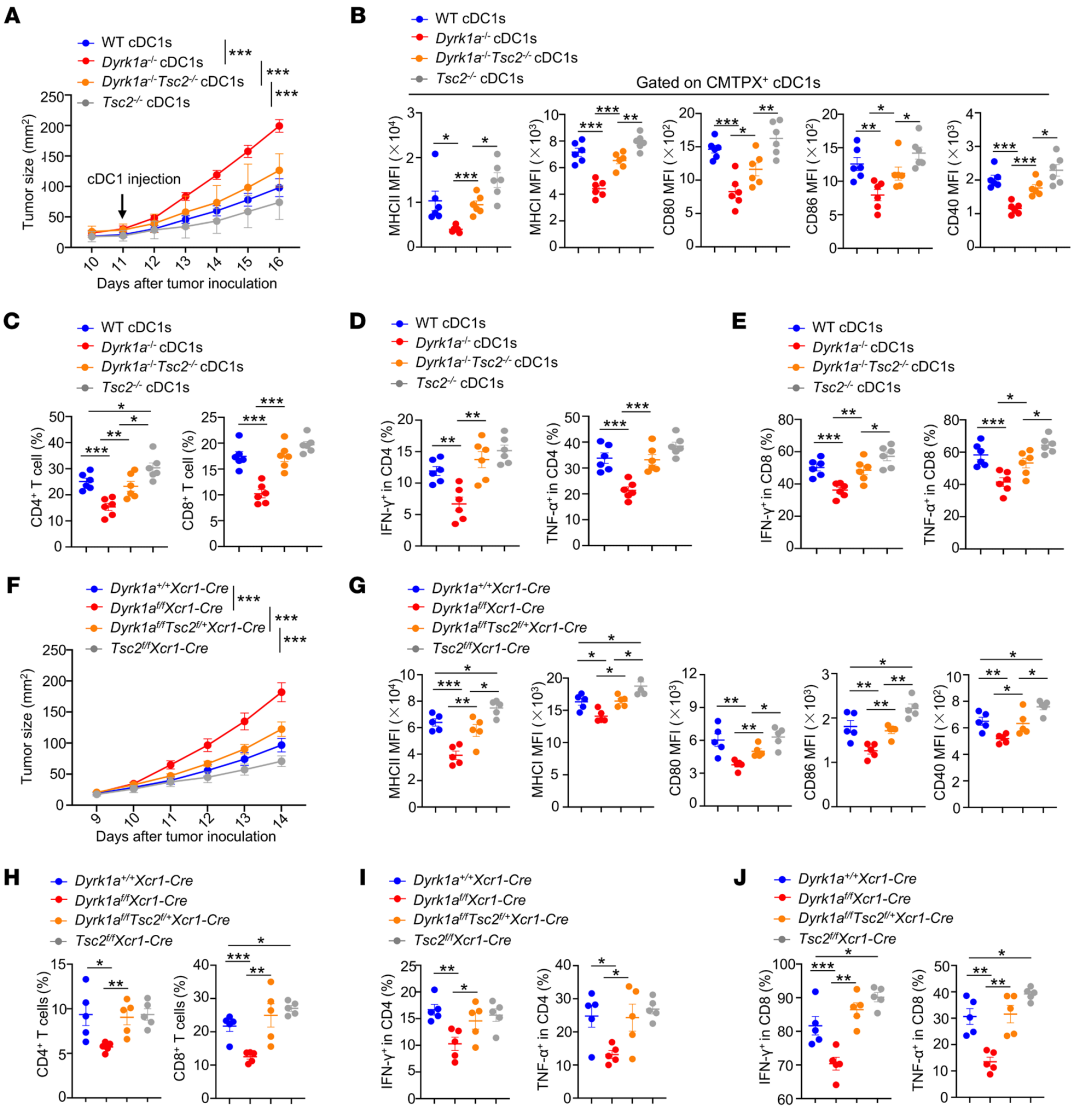
Deletion of TSC2 in *Dyrk1a*-deficient cDC1 restores its antitumor function. (**A**) Tumor growth curves of wild-type mice s.c. injected with B16-F10 melanoma cells and then treated i.t. with WT, *Dyrk1a^–/–^*, *Dyrk1a^–/–^* *Tsc2^–/–^*, and *Tsc2^–/–^* BM-cDC1s. BM-cDC1s were pulsed with tumor cell lysates and matured with Poly(I:C) (*N* = 6 mice/group). (**B**) Flow cytometric analysis of MHCI, MHCII, CD80, CD86, and CD40 on tumor-infiltrating CMTPX-labeled cDC1s at day 16 after tumor inoculation. (**C**) Flow cytometric analysis of the frequencies of tumor-infiltrating CD4^+^ and CD8^+^ T cells. (**D** and **E**) Flow cytometric analysis of the frequencies of IFN-γ–, TNF-α–producing CD4^+^ T cells (**D**) and CD8^+^ T cells (**E**) in the tumors of tumor-bearing mice i.t. injected with indicated cDC1s. (**F**) Tumor growth curves of 6-week-old *Dyrk1a^+/+^* *Xcr1*-Cre, *Dyrk1a^fl/fl^* *Xcr1*-Cre, *Dyrk1a^fl/fl^* *Tsc2^fl/+^* *Xcr1*-Cre, and *Tsc2^fl/fl^* *Xcr1*-Cre mice following s.c. injection with B16-F10 cancer cells (*N* = 5 mice/group). (**G**) Flow cytometric analysis of MHCI, MHCII, CD80, CD86, and CD40 on tumor-infiltrating cDC1s from *Dyrk1a^+/+^* *Xcr1*-Cre, *Dyrk1a^fl/fl^* *Xcr1*-Cre, *Dyrk1a^fl/fl^* *Tsc2^fl/+^Xcr1*-Cre, and *Tsc2^fl/fl^* *Xcr1*-Cre mice at day 14 after tumor inoculation. (**H**) Flow cytometric analysis of the frequencies of tumor-infiltrating CD4^+^ and CD8^+^ T cells. (**I** and **J**) Flow cytometric analysis of the frequencies of IFN-γ–, TNF-α–producing CD4^+^ T cells (**I**) and CD8^+^ T cells (**J**) in the tumors of tumor-bearing mice. Data are representative of 3 independent experiments. Summary data are shown as the mean ± SEM. *P* values were determined using 1-way ANOVA (**B**–**E** and **G**–**J**) or 2-way ANOVA (**A** and **F**). *P < 0.05; **P < 0.01; ***P < 0.001.

DC-based immunotherapy has emerged as a promising approach in cancer treatment (3). To further analyze the functional association of TSC2 and DYRK1A in vivo, we employed an animal model of cDC1-based therapy. After inoculation of B6 mice with B16-F10 cells, we intratumorally injected tumor lysate-pulsed wild-type, *Dyrk1a*^–/–^, *Dyrk1a*^–/–^ *Tsc2*^–/–^, or *Tsc2*^–/–^ cDC1s into the tumor-bearing mice. Compared with *Dyrk1a*-deficient cDC1s, wild-type and *Tsc2*-deficient cDC1s were more effective at suppressing tumor growth and inducing tumor-infiltrating effector T cells (Figure 8, A and C). Importantly, TSC2 ablation in *Dyrk1a*-deficient cDC1s markedly restored T cell infiltration and inhibited tumor growth (Figure 8, A and C). Notably, TSC2 loss in *Dyrk1a*-deficient cDC1s significantly elevated MHC and costimulatory molecule expression on tumor-infiltrating cDC1s (Figure 8B). Consistently, tumor-infiltrating CD8^+^ T cells from mice treated with *Dyrk1a*^–/–^ *Tsc2*^–/–^ cDC1s showed restored proliferation and reduced surface PD-1 and TIM-3 expression compared with those from *Dyrk1a*^–/–^ cDC1–treated mice (Supplemental Figure 23D). Functionally, tumor-infiltrating T cells from the tumor-bearing mice treated with *Dyrk1a*^–/–^ *Tsc2*^–/–^ cDC1s produced remarkably higher levels of IFN-γ and TNF-α than those from the tumor-bearing mice treated with *Dyrk1a*^–/–^ cDC1s (Figure 8, D and E). To validate the functional role of the DYRK1A/TSC2 axis in regulating mTORC1 signaling in cDC1s, we performed comparative analysis of mTORC1 activation in wild-type, *Dyrk1a*^–/–^, and *Dyrk1a*^–/–^ *Tsc2*^–/–^ cDC1s (Supplemental Figure 23E). Consistent with our previous findings, DYRK1A ablation substantially inhibited mTORC1 activity, as demonstrated by significantly decreased phosphorylation levels of S6K1, ribosomal protein S6, and 4EBP1 in *Dyrk1a*-deficient cDC1s (Supplemental Figure 23E). Importantly, TSC2 deletion fully restored mTORC1 activation in *Dyrk1a*-deficient cDC1s, with phosphorylation levels of these targets returning to wild-type levels. These data illustrate that DYRK1A regulates mTORC1 signaling through TSC2 in cDC1s.

To further confirm the role of TSC2 in the functional defects of *Dyrk1a*-deficient cDC1s, we inoculated age- and sex-matched wild-type, *Dyrk1a^fl/fl^* *Xcr1*-Cre, *Dyrk1a^fl/fl^* *Tsc2^fl/+^Xcr1*-Cre, and *Tsc2^fl/fl^* *Xcr1*-Cre mice with B16-F10 tumors. Compared with *Dyrk1a^fl/fl^* *Xcr1*-Cre mice, the *Dyrk1a^fl/fl^* *Tsc2^fl/+^* *Xcr1*-Cre mice exhibited substantially reduced tumor size and increased infiltration of effector T cells (Figure 8, F and H). Tumor-infiltrating cDC1s from *Tsc2^fl/fl^* *Xcr1*-Cre mice expressed significantly higher levels of MHC and costimulatory molecules than those from wild-type controls (Figure 8G). Moreover, TSC2 ablation in *Dyrk1a*-deficient cDC1s remarkably enhanced the expression levels of MHC and costimulatory molecules in tumor-infiltrating cDC1s (Figure 8G). Accordingly, tumor-infiltrating CD8^+^ T cells from the tumor-bearing *Dyrk1a^fl/fl^* *Tsc2^fl/+^* *Xcr1*-Cre mice exhibited restored proliferation levels and decreased surface expression of PD-1 and TIM-3, compared with those from the tumor-bearing *Dyrk1a^fl/fl^* *Xcr1*-Cre mice (Supplemental Figure 23F). In addition, tumor-infiltrating T cells from *Dyrk1a^fl/fl^* *Tsc2^fl/+^* *Xcr1*-Cre mice produced significantly higher levels of IFN-γ and TNF-α than those from *Dyrk1a^fl/fl^* *Xcr1*-Cre mice (Figure 8, I and J). Taken together, these data demonstrate that TSC2 deletion restores the compromised immune function of *Dyrk1a*-deficient cDC1s, thereby promoting the antitumor immune responses.

### DYRK1A/TSC2/mTORC1 axis in cDC1s is essential for antitumor immunity in human cancers.

**Figure 9:**
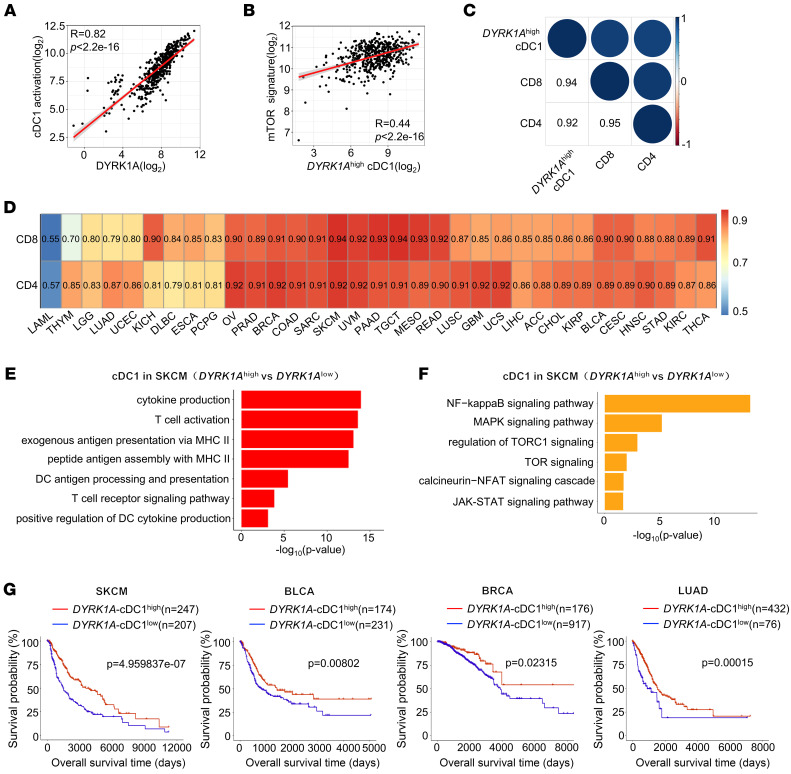
DYRK1A/TSC2/mTORC1 axis in cDC1s is essential for antitumor immunity in human cancers. (**A**) Scatterplot showing the correlation of cDC1 activation and *DYRK1A* expression level based on TCGA SKCM cohort. (**B**) Scatterplot showing the association of mTOR signature and *DYRK1A* expression level in tumoral cDC1s based on the TCGA SKCM cohort. (**C**) Spearman’s correlation between CD4^+^ and CD8^+^ T cell responses and *DYRK1A* expression level in tumoral cDC1s in melanoma TCGA datasets. (**D**) Heatmap showing the correlation between CD4^+^ and CD8^+^ T cell responses and *DYRK1A* expression levels in tumoral cDC1s across various cancer types. (**E** and **F**) Functional enrichment analysis of Gene Ontology (**E**) and Kyoto Encyclopedia of Genes and Genomes pathways (**F**) that were significantly changed in *DYRK1A*^hi^ cDC1s compared with *DYRK1A*^lo^ cDC1s in patients with SKCM. (**G**) Overall survival of TCGA SKCM, BLCA, BRCA, and LUAD cohorts based on the expression levels of *DYRK1A* and cDC1 signature genes. *P* values (Wald’s χ^2^ test) were determined based on a univariate Cox proportional-hazards model (*DYRK1A*-cDC1^hi^ versus *DYRK1A*-cDC1^lo^).

Given the potential importance of the DYRK1A/TSC2 axis in cDC1-mediated antitumor activity, we examined the relationship between DYRK1A and cDC1 function in human SKCM. Notably, DYRK1A expression in tumor-infiltrating cDC1s positively correlated with cDC1 activation and mTORC1 signaling (Figure 9, A and B). Moreover, *DYRK1A*^hi^ tumor-infiltrating cDC1s were strongly associated with effector CD4^+^ and CD8^+^ T cell responses in SKCM (Figure 9C), suggesting a role for cDC1 DYRK1A in promoting effector T cell immunity. Extending this analysis to multiple cancer types in TCGA database, we observed that *DYRK1A*^hi^ cDC1s are positively correlated with effector T cell responses across human cancers (Figure 9D).

To understand how DYRK1A regulates cDC1 function in human cancers, we performed a functional pathway enrichment analysis using single-cell RNA-sequencing data from patients with melanoma (53). We found that DYRK1A in cDC1s positively regulated cytokine production, T cell activation, exogenous antigen presentation, and peptide antigen assembly (Figure 9E). In addition, DYRK1A modulated the mTOR signaling pathway (Figure 9F). Next, we examined whether DYRK1A expression in cDC1s is associated with survival outcomes in patients with cancer using TCGA dataset. Notably, patients in the *DYRK1A*-cDC1^hi^ group showed significantly improved overall survival than those in the *DYRK1A*-cDC1^lo^ group across SKCM, BLCA, BRCA, and LUAD (Figure 9G). We then sought to clarify the regulatory relationship between DYRK1A and TSC2. TCGA analysis revealed that *TSC2* mRNA levels alone did not stratify patient survival in these cancer types (Supplemental Figure 24A) and were comparable between normal and tumor tissues (Supplemental Figure 24B). Consistent with this, *Tsc2* transcript levels in tumor-infiltrating cDC1s remained unchanged upon *Dyrk1a* deletion (Supplemental Figure 24C). These results support our model that DYRK1A regulates antitumor immunity primarily by posttranslationally phosphorylating TSC2, promoting its degradation without affecting its transcription. Collectively, these findings demonstrate that the DYRK1A/TSC2/mTORC1 axis in cDC1s plays an essential role in regulating antitumor immunity and cancer progression.

## Discussion

In this study, we demonstrate that DYRK1A enhances mTORC1 signaling in DCs and promotes antitumor immunity. DYRK1A deficiency in DCs impairs T cell responses, leading to increased tumor burden and mortality. Notably, DYRK1A regulates DC function in a subset-specific manner. Specifically, it enhances the maturation and antigen-presenting capacity of cDC1s, while exerting minimal effects on cDC2. Mechanistically, DYRK1A phosphorylates TSC2 at Ser540, facilitating its interaction with the E3 ubiquitin ligase HUWE1, which drives TSC2 polyubiquitination and degradation. Deletion of TSC2 in *Dyrk1a*-deficient cDC1s restores their antitumor immune functions, whereas pharmacological inhibition of mTORC1 phenocopies the effects of DYRK1A loss. Collectively, our findings highlight a pivotal role of DYRK1A/TSC2/mTORC1 signaling axis in regulating cDC1 activation and function in the context of antitumor immunity.

Within the TME, malignant cells deplete essential nutrients, including amino acids and growth factors, thereby imposing metabolic constraints that drive functional and metabolic reprogramming of tumor-infiltrating immune cells (54, 55). We found that DYRK1A expression was markedly induced upon DC activation, and its genetic deletion substantially impaired DC activation and maturation. These observations suggest that DYRK1A may function as a nutrient-sensing modulator that integrates microenvironmental cues to orchestrate downstream signaling and DC functionality. Functional characterization demonstrated that DYRK1A specifically potentiates the maturation and antigen-presenting capacity of cDC1s, with little effect on cDC2 subsets. We propose that this subset-specific dependence on DYRK1A activity may reflect divergent mechanisms for sensing extracellular nutrients or growth factors between cDC1 and cDC2 populations, potentially echoing their specialized immunological roles. However, the precise molecular mechanisms underlying these observations warrant further investigation.

The mTORC1 signaling network serves as a critical sensor of environmental cues and regulates diverse cellular processes. However, the role of mTORC1 in DC function remains ambiguous (56), likely due to discrepancies in animal models, experimental systems, and/or genetic approaches. Emerging evidence points to subset-specific regulatory mechanisms. For instance, *Raptor* deficiency in *Cd11c*-Cre mice disrupts mTORC1 signaling in APCs and enhances their T cell–priming capacity, correlating with increased activation of LCs and a distinct EpCAM^+^ cDC1 subset (40). Paradoxically, the same study showed impaired cross-presentation capability in *Raptor*-deficient cDC1s, underscoring the cell type–specific modulation of DC function by mTORC1. Additionally, Sinclair et al. demonstrated that pulmonary CD103^+^ cDC1 homeostasis depends on mTOR signaling, and APC-specific mTOR ablation shifts immune responses from type 2 to type 17 following allergen challenge (42). Our study extends these observations by identifying the DYRK1A/mTORC1 axis as a subset-specific regulator of DC function. Using *Xcr1*-Cre mice, which provide superior cDC1-targeting specificity, we demonstrate that this axis selectively enhances cDC1 maturation and antigen-presenting capacity, with negligible effects on cDC2s. Importantly, unlike prior studies using infection or allergy models, our tumor model reveals distinct context-dependent regulation of cDC1 function by mTORC1 signaling. Through an integrated approach that combines genetic mouse models, biochemical analyses, and pharmacological interventions, we establish the DYRK1A/mTORC1 signaling pathway as a critical regulator of cDC1s in tumor immunity. Collectively, our work extended previous findings by employing lineage-specific genetic tools, examining tumor-relevant contexts, and identifying DYRK1A as the upstream regulator of mTORC1 signaling in cDC1s.

Our study identifies Ser540 as the primary phosphorylation site targeted by DYRK1A. Although a previous report suggested that DYRK1A phosphorylates TSC2 at Thr1462 to inhibit TSC complex function and enhance mTORC1 signaling (49), those findings lacked direct evidence linking phosphorylation at Thr1462 to mTORC1 regulation, and the physiological relevance of this modification remained unaddressed. It is worth mentioning that our unbiased proteomic screening in cDC1s reveals that Thr1462 is not the major DYRK1A target. Instead, DYRK1A phosphorylates TSC2 at Ser540, promoting its interaction with the E3 ubiquitin ligase HUWE1 and subsequent proteasomal degradation. This mechanism is functionally important, as *Tsc2* deletion fully restores the immunostimulatory capacity of *Dyrk1a*-deficient cDC1s. These findings reveal an additional layer of mTORC1 regulation in cDC1s.

How does the DYRK1A/mTORC1 axis mechanistically dictate cDC1 function? Our study bridges this gap by linking kinase activity to intracellular antigen processing in cDC1s. DYRK1A deficiency leads to aberrant lysosomal biogenesis and excessive phagosomal acidification, thereby accelerating antigen degradation within lysosomes. Concurrently, the proteasomal processing pathway required for cross-presentation is impaired. By phosphorylating TSC2, DYRK1A sustains mTORC1 activity, thereby tuning the lysosomal and proteasomal machinery to an optimal state for preserving antigenic peptides. These findings align with and extend previous reports that precise regulation of proteolysis is essential for effective cross-presentation (44, 57).

Our study further reveals that DYRK1A potentiates the antitumor immune response of cDC1s by modulating mTORC1 signaling, offering mechanistic insights into cDC1-based cancer immunotherapy, a promising therapeutic approach (3, 10, 58). Genetic mouse models show that TSC2 ablation fully rescues the antitumor function of *Dyrk1a*-deficient cDC1s. Clinically, DYRK1A expression in tumor-infiltrating cDC1s is strongly associated with enhanced effector T cell responses across multiple human cancers, highlighting the translational relevance of this pathway to antitumor immunity and disease progression. Given the current lack of TSC2-specific inhibitors, developing compounds that enhance DYRK1A activity or inhibit TSC2 function in tumor-infiltrating cDC1s represents a strategic future direction to potentiate antitumor immunity. Our findings establish the DYRK1A/TSC2/mTORC1 axis as a crucial regulator of cDC1 function in antitumor immunity, thus revealing a promising therapeutic avenue for cancer immunotherapy.

## Methods

### Sex as a biological variable.

Our study included both male and female animals, and similar findings were observed in both sexes.

### Mice.

*Dyrk1a*-floxed (027801), *Cd11c*-Cre (008068), *Xcr1*-Cre (035435), OT-I (003831), OT-II (004194), and *β2m*^−/−^ (002087) mice were originally from the Jackson Laboratory. *Dyrk1a*-floxed mice were generated using the *loxP* system targeting exons 5 and 6 of the *Dyrk1a* gene. The *Dyrk1a*-floxed mice were crossed with *Cd11c*-Cre transgenic mice to generate *Dyrk1a* DC-conditional knockout (*Dyrk1a^fl/fl^ Cd11c*-Cre, referred to as *Dyrk1a*-DC-cKO) mice and wild-type control mice (*Dyrk1a*^+/+^ *Cd11c*-Cre). To generate cDC1-specific *Dyrk1a*-knockout (referred to as *Dyrk1a*-cDC1-cKO) mice, we crossed *Dyrk1a*-floxed mice with *Xcr1*-Cre transgenic mice, and the heterozygotes of *Xcr1*-Cre mice were used in our experiments. The *Tsc2*-floxed mice (T052244) were generated at GemPharmatech company (Jiangsu, China) and crossed with *Dyrk1a^fl/fl^* *Xcr1*-Cre mice to produce *Dyrk1a^fl/fl^* *Tsc2^fl/+^* *Xcr1*-Cre mice. The mice used in this study were co-caged, sex- and age-matched littermates, unless otherwise stated. Genotyping PCR was performed using the primers listed in Supplemental Table 1. All mice were maintained under specific pathogen–free conditions at the animal facility of Xiamen University.

### Antigen presentation assays.

Antigen presentation was examined using established in vivo and in vitro systems (59, 60). For the in vivo assay, sex- and age-matched wild-type and *Dyrk1a*-cDC1-cKO mice (8–10 weeks old) first received an adoptive transfer of 1 × 10^6^ CFSE-labeled OT-I T cells via the tail vein. After 24 hours, these mice were intravenously administered 1 × 10^6^ irradiated (1,500 rad), OVA-loaded *β2m*^–/–^ splenocytes or OVA protein (20 μg). The OVA-loaded *β2m*^–/–^ splenocytes were prepared by permeabilization and pulsing with 10 mg/mL soluble OVA. The proliferation of OT-I T cells in the spleen and peripheral LNs was analyzed 72 hours later by flow cytometry using CFSE dilution. For the in vitro antigen presentation assay, 0.4 × 10^5^ bone marrow–derived cDC1 or cDC2 cells (sorted from Flt3L-cultured bone marrow cells) were cocultured for 72 hours with 2 × 10^5^ CFSE-labeled OT-I or OT-II T cells. The cultures were stimulated with SIINFEKL peptide, soluble OVA, or HKLM-OVA. T cell proliferation was quantified by flow cytometric analysis based on CFSE dilution. *Listeria monocytogenes* engineered to express OVA (LM-OVA; Nanjing Sungyee Inc.) was propagated in brain-heart infusion broth at 37°C. The bacteria were then washed extensively with PBS, frozen overnight, and subsequently heat-inactivated at 80°C for 1 hour. The final HKLM-OVA preparation was stored at –80°C for future use.

### In vivo T cell priming.

For evaluation of T cell priming, mice were subcutaneously inoculated in the hind footpads with 5 × 10^5^ BM-cDC1s, which had been pretreated with B16-GP33 tumor cell lysate in the presence of Poly(I:C). Seven days later, draining popliteal LNs were harvested and analyzed for antigen-specific CD8^+^ T cell populations by tetramer staining. For tetramer staining, cells were incubated with the H-2D^b^/GP33-41 MHCI tetramer before any additional surface staining. For intracellular cytokine staining, cells were stimulated with the GP33-41 peptide for 8 hours, and intracellular IFN-γ, TNF-α, and granzyme B in CD8^+^ T cells were analyzed by flow cytometry.

### BM-cDC1 transfer for tumor therapy.

For cDC1 transfer experiments, B16-F10 tumor–bearing wild-type mice were treated with intratumoral injections of 1 × 10^6^ BM-cDC1s at the indicated time point, which were pretreated with Poly(I:C) and tumor cell lysates. For in vivo detection of BM-cDC1 function, primed BM-cDC1s were labeled with 5 μM CellTrace Red CMTPX (Thermo Fisher Scientific) for 20 minutes at 37°C before intratumoral injection. Tumor growth was monitored daily following BMDC therapy to assess therapeutic efficacy.

### Bulk RNA-sequencing analysis.

For tumor-infiltrating DC sequencing, the DCs (CD3^–^CD19^–^NK1.1^–^Ly6C^–^Ly6G^–^F4/80^–^CD11c^+^MHCII^+^) or cDC1s (CD3^–^CD19^–^NK1.1^–^Ly6C^–^Ly6G^–^F4/80^–^CD11c^+^XCR1^+^) were sorted from tumor tissues of B16-F10 tumor–bearing *Dyrk1a^+/+^ Cd11c*-cre mice and *Dyrk1a^fl/fl^ Cd11c*-cre mice. Purified DCs were used for total RNA extraction with TRIzol (Invitrogen), followed by RNA sequencing using the Illumina Nextseq 500 platform (75 bp paired-end reads). The raw sequencing reads were aligned to the mouse reference genome (version mm10) using Hisat2 RNASeq alignment software, achieving an average mapping rate of 96% across all samples in the dataset. Gene expression counts were quantified from TopHat2 alignment files using HTSeq. Differential expression analysis was then performed on the count data with the R package DESeq2, and *P* values from multiple tests were adjusted using the Benjamini-Hochberg correction.

### Statistics.

Statistical analysis was conducted using Prism software (GraphPad Software). Significant differences between the 2 groups were analyzed with a 2-tailed unpaired *t* test. Multiple groups were analyzed using 1-way ANOVA, where applicable, to determine whether an overall statistically significant difference existed, followed by 2-tailed unpaired Student’s *t* tests to compare any 2 groups. Tumor growth curves were analyzed by 2-way ANOVA. A log-rank (Mantel-Cox) test was used for comparison of survival curves. The significance level was *P* < 0.05.

### Study approval.

Animal experiments were conducted in accordance with protocols approved by the Institutional Animal Care and Use Committee of Xiamen University.

### Data availability.

All graph data points reported in the manuscript and supporting information are provided in the Supporting Data Values file. Bulk RNA-seq data have been deposited in the GEO public database with accession numbers GSE281370 and GSE327455. The mass spectrometry datasets have been deposited in the iProX repository (accession numbers PXD057773 and PXD057775).

Further information can be found in Supplemental Methods.

## Author contributions

ZJ and HW conceived and designed research studies. HW performed the experiments, prepared the figures, and wrote the manuscript. HJ, SH, SR, HL, WL, CZ, PZ, KC, and WC contributed to the experiments and data analysis. YQ, DD, NX, HH, CJK, YZ, BW, and QZ provided essential reagents, provided scientific advice, and contributed to the experiments. JHS, XL, and ZJ supervised the work, acquired funding, and wrote the manuscript.

## Conflict of interest

The authors have declared that no conflict of interest exists.

## Funding support

- The National Key Research and Development Program of China, 2022YFA1304003 (ZJ).
- The National Natural Science Foundation of China, 32470970 (ZJ), 81871305 (XL), 82273463 (JHS), and 32370731 (YZ).
- The Key Healthcare Projects of Xiamen City, 3502Z20234008 (XL).
- The Scientific Research Foundation of State Key Laboratory of Vaccines for Infectious Diseases, Xiang An Biomedicine Laboratory, 2023XAKJ0101027 (ZJ) and 2025XAKJ0102016 (XL).
- The Fundamental Research Funds for the Central Universities, 20720210113 (ZJ) and 20720220003 (ZJ).

## Supplementary Material

Supplemental data

Unedited blot and gel images

Supporting data values

## Version 1 04/23/2026

In-Press Preview

## Version 2 06/15/2026

Electronic publication
