# Supplementary material for: DYRK1A enhances antitumor immunity in type 1 conventional dendritic cells via mTORC1 activation
Source: J Clin Invest. 2026 Apr 23;136(12):e199108. doi: 10.1172/JCI199108 (PMC13262734; doi:10.1172/JCI199108)
Supplement: Supplemental data [file jci-136-199108-s261.pdf]

DYRK1A enhances antitumor immunity in type 1 conventional dendritic cells via mTORC1 activation

Hongjiao Wang[1,9], He Jiang[1,9], Songlin He[2,9], Songwen Ren[1,9], Haiwen Li[3], Wangnan Liu[1], Chunyun Zhou[4], Pan Zhu[1], Keren Chen[1], Weijia Cao[1], Yan Qin[3], Dan Du[5], Nengming Xiao[1], Hongling Huang[1], Chun-Jung Ko[6], Yiming Zheng[1], Bo Wang[1], Qiang Zou[7], Jian-Hong Shi[3*], Xun Li[2*], Zuliang Jie[1,8*]

[1]State Key Laboratory of Cellular Stress Biology, Department of Oncology, Xiang'an Hospital of Xiamen University, School of Life Sciences, Faculty of Medicine and Life Sciences, Xiamen University, Xiamen, Fujian, China.

[2]Department of Laboratory Medicine, Xiamen Key Laboratory of Genetic Testing, The First Affiliated Hospital of Xiamen University, School of Medicine, Xiamen University, Xiamen, Fujian, China.

[3]Central Laboratory, Hebei Collaborative Innovation Center of Tumor Microecological Metabolism Regulation, Affiliated Hospital of Hebei University, Clinical Medical College, Hebei University, Baoding, Hebei, China.

[4]School of Public Health, Xiamen University, Xiamen, Fujian, China.

[5]Department of Stomatology, Cancer Research Center, School of Medicine, Xiamen University, Xiamen, Fujian, China.

[6]Graduate Institute of Immunology, College of Medicine, National Taiwan University, Taipei, Taiwan.

[7]Shanghai Institute of Immunology, Department of Immunology and Microbiology, Shanghai Jiao Tong University School of Medicine, Shanghai, China.

[8]State Key Laboratory of Vaccines for Infectious Diseases, Xiang An Biomedicine Laboratory, School of Public Health, Xiamen University, Fujian, China.

[9]These authors contributed equally to this work.

***Corresponding authors:**

Zuliang Jie, State Key Laboratory of Cellular Stress Biology, Department of Oncology, Xiang'an Hospital of Xiamen University, School of Life Sciences, Faculty of Medicine and Life Sciences, Xiamen University, Xiamen, Fujian, 361102, China; Tel: 86-13606027923; Email: jiezuliang@xmu.edu.cn.

Xun Li, Department of Laboratory Medicine, Xiamen Key Laboratory of Genetic Testing, The First Affiliated Hospital of Xiamen University, School of Medicine, Xiamen University, Xiamen, Fujian, 361000, China; Tel: 86-592-2139657; Email: xli2001@xmu.edu.cn.

Jian-Hong Shi, Central Laboratory, Hebei Collaborative Innovation Center of Tumor Microecological Metabolism Regulation, Affiliated Hospital of Hebei University, Clinical Medical College, Hebei University, Baoding, Hebei, 071000, China; Tel: 86-312-5981629; Email: shijianhong@hbu.edu.cn.

SUPPLEMENTAL METHODS

Plasmids, antibodies, and reagents

Flag-tagged mouse WT, S540A, and S540D TSC2 were cloned into the pcDNA3.3 vector. HA-tagged mouse WT and K188R DYRK1A were cloned into the pcDNA3.3 vector. The pcDNA expression vectors encoding HA-tagged DYRK1A truncation mutants (DYRK1A Δ1 to Δ5) were created by PCR using a mouse DYRK1A template and subcloned into the pcDNA3.3 vector. The pcDNA expression vectors encoding Flag-tagged TSC2 truncation mutants (TSC2 Δ1 to Δ5) were created by PCR using a mouse TSC2 template and subcloned into the pcDNA3.3 vector.

Antibodies for p52/p100 (4882), p50/p105 (13586), RelB (10544), p-S6K (9205), p-S6K (9204), p-S6 (2211), p-4EBP1 (2855), DYRK1A (8765), TSC1 (6935), TSC2 (4308), p-TSC2 (3617), FoxO1 (2880) and p-FoxO1 (9461) were purchased from Cell Signaling Technology, Inc. Anti-Lamin B (sc-374015) and anti-p65 (sc-136548) were from Santa Cruz Biotechnology, Inc. Anti-Flag (F1804) antibody was from Sigma-Aldrich, Inc. Antibodies for β-Actin (AC026), HSP60 (A0564), HA (AE008) and HUWE1 (A20708) were purchased from ABclonal, Inc. Antibodies for IRF1 (11335-1-AP) were purchased from Proteintech, Inc. Antibodies for IRF5 (ab181553) were purchased from Abcam, Inc. The fluorochrome-conjugated antibodies for CD4 (8C3), CD8 (53-6.7), CD62L (MEL-14), CD326 (G8.8), CD103 (2E7), XCR1 (ZET), H-2Kb (AF6-88.5), PD-1 (J43), CXCR3 (173), XCR1 (1C6), CD11c (N418), CD172a (P84), IL-12p40 (C15.6), I-A/I-E (M5/114.15.2), CD40 (1C10), and CD80 (16-10A1) were purchased from Biolegend, Inc. FITC anti-mouse IFN-γ (XMG1.2), PE anti-mouse TNF-α (MP6-XT22), PE anti-mouse TIM-3 (F38-2E2), APC anti-mouse CD86 (GL-1), APC anti-mouse CD317 (129c), PerCP-Cyanine5.5 anti-mouse CD44 (IM7), Alexa Fluor 488 anti-mouse CD207 (eBioRMUL.2), and APC anti-mouse Granzyme B (NGZB) were purchased from eBioscience, Inc. Phospho-S6 ribosomal protein (Ser235/236; D57.2.2E) XP Rabbit mAb (APC conjugate) was purchased from Cell Signaling Technology, Inc. Rapamycin (9904S) were purchased from Sigma-Aldrich, Inc. MG-132 (HY-13259), Bafilomycin A1 (HY-100558), and

cycloheximide (HY-12320) were purchased from Med Chem Express, Inc. PE conjugated MHC-I tetramers H-2D^b / GP33-41 were purchased from Reagent platform of Cancer Research Center of Xiamen University.

Cell lines and cell culture

Human embryonic kidney HEK293T cells (ATCC), MC38/MC38-OVA murine colon cancer cells (Mensen), B16-F10/B16-GP33 murine melanoma cells (ATCC), MB49 murine bladder cancer cells (ATCC), and murine DC line DC2.4 cells (Sigma-Aldrich) were cultured *in vitro*. HEK293T, MC38, and MB49 cells were cultured in DMEM medium supplemented with 10% FBS and 100 units/mL penicillin-streptomycin. B16-F10 murine melanoma cells and DC2.4 cells were cultured in RPMI 1640 medium supplemented with 10% FBS and 100 units/mL penicillin-streptomycin. All cell lines were cultured at 37°C in a humidified incubator with 5% CO_2. B16-GP33 cells were provided by Nengming Xiao from School of Life Sciences, Xiamen University.

***In vitro* stimulation of DCs**

For *in vitro* stimulation, DCs were treated with different TLR agonists—including LPS (100 ng/mL), Pam3CSK4 (1 μg/mL), Poly (I:C) (20 μg/mL), R848 (1 μg/mL), and CpG (10 μM)—as well as EGF (100 ng/mL), tumor DNA (40 μg/mL), IFN-γ (30 ng/mL) for the indicated time points. For amino acid stimulation, DCs were first cultured in amino acid-free medium supplemented with 10% dialyzed FBS for 2 hours before harvest (1). Subsequently, cells were incubated in complete RPMI-1640 medium containing all 20 essential and non-essential amino acids (including leucine, lysine, cysteine, arginine, L-glutamine, etc.).

Preparation of BMDCs

BMDCs were generated by culturing bone marrow cells from age- and sex-matched wild-type and *Dyrk1a*-DC-cKO mice in a growth medium supplemented with recombinant FLT3L (200 ng/mL). Fresh medium containing FLT3L was added on day 3 of the culture, and the fully

differentiated DCs were harvested on day 9 for subsequent assays. The generated DC population was analyzed for CD11c expression using flow cytometry and further enriched with CD11c microbeads (Miltenyi Biotec).

***In vitro* generation of bone marrow-derived cDC1s (BM-cDC1s)**

For *in vitro* generation of cDC1s from mouse bone marrow, we adapted an established protocol (2). In brief, bone marrow cells were cultured at 1.5 × 10^6 cells/mL in complete RPMI 1640 medium supplemented with 10% heat-inactivated FBS, 1 mM sodium pyruvate, 50 μM β-mercaptoethanol, 1 % penicillin-streptomycin, 100 ng/mL FLT3L, and 2 ng/mL murine GM-CSF. On day 5, fresh basal medium (containing FBS, sodium pyruvate, β-mercaptoethanol, and penicillin-streptomycin) was added to the existing culture. At day 9, non-adherent cells were harvested and reseeded in fresh supplemented medium. On day 17, cDC1s (identified as $CD45^+CD11c^+B220^-XCR1^+CD172^-CD103^+$ population) were purified from non-adherent cells by FACS sorter (FACSAria III or FACSAria Fusion, BD Biosciences).

Tumor models

B16-F10 and B16-GP33 murine melanoma cells were cultured in RPMI 1640 medium supplemented with 10% FBS and 100 U/mL penicillin-streptomycin. MC38 murine colon cancer cells and MB49 murine bladder cancer cells were cultured in DMEM supplemented with 10% FBS and 100U/mL penicillin-streptomycin. For *in vivo* tumor growth experiments, age- and sex-matched wild-type, *Dyrk1a*-DC-cKO mice were injected subcutaneously with B16F10 murine melanoma cells (2 × 10^5 cells), B16-GP33 murine melanoma cells (2 × 10^5 cells), or MC38 colon cancer cells (1 × 10^6 cells), and monitored for tumor growth and survival. Age- and sex-matched wild-type and *Dyrk1a*-cDC1-cKO mice were injected subcutaneously with B16-GP33 murine melanoma cells (2 × 10^5 cells) or MB49 murine bladder cancer cells (1 × 10^6 cells), and monitored for tumor growth. Mice with tumor size reaching 225 mm^2 were considered lethal and euthanized according to protocols approved by the Institutional Animal

Care and Use Committee of Xiamen University. Thus, lethality was defined as tumor size reaching 225 mm^2. At the indicated time point, all mice were euthanized for flow cytometry analysis of immune cells from the draining lymph nodes and tumors. To minimize individual variations, seven to twelve age- and sex-matched wild-type, *Dyrk1a*-DC-cKO, or *Dyrk1a*-cDC1-cKO mice were used per experimental group. In the orthotopic mouse model of colorectal cancer, mice were anesthetized, and the cecum was carefully exposed and flattened. Subsequently, a 31-gauge needle was used to inject 10 μL of the MC38 cell suspension (containing $2×10^5$ cells) into the cecal wall. Following the injection, the cecum was gently returned to the peritoneal cavity, and the peritoneal wall was sutured. Tumors were harvested from the cecum approximately 30 days later for subsequent analysis (3). For *in vivo* $CD8^+$ T cell depletion, anti-CD8α (YTS 169.4; BioXCell BE0117) or isotype control antibody (LTF-2; BioXCell BE0090) was diluted to 1 mg/ml in PBS, and 200 μl was administered per mouse by intraperitoneal injection on days −1, 2, 6, and 10 relative to the day of B16-F10 injection (4).

Flow cytometry and intracellular cytokine staining

The single-cell suspension of immune cells was incubated with anti-CD16/CD32 antibody (clone 93; BioLegend) to block nonspecific binding via Fc receptors and then stained with fluorochrome-labeled antibodies. To determine intracellular cytokine expression, total cells were stimulated with PMA, ionomycin, and monensin for 5 hours. After incubation, cells were stained with a fixable viability dye, blocked with FcγR blocker (CD16/32), and stained for specific surface molecules. After surface staining, cells were fixed, permeabilized, and stained for intracellular cytokines by using a fixation/permeabilization kit (BD Biosciences). Samples were processed on an LSRFortessa (Becton Dickinson). Counting beads (BioLegend) were added to quantify the absolute cell numbers, and data analysis was performed using FlowJo software (TreeStar).

ELISA analysis

For cytokine analysis by ELISA, equal numbers of wild-type and *Dyrk1a*-deficient BMDCs or BM-cDC1s were stimulated with LPS or Poly(I:C) for 12 hours. Supernatants from wild-type and *Dyrk1a*-DC-cKO BMDCs were analyzed for IL-6, IL-1β, IL-10, and CXCL9 production. Supernatants from wild-type and *Dyrk1a*-cDC1-cKO BM-cDC1s were assessed for IL-6 and IL-1β secretion using commercial ELISA kits (Thermo Fisher Scientific) according to the manufacturer’s instructions.

RNA isolation and Real-time qRT-PCR

Total RNA was isolated from BMDCs using TRIzol (Invitrogen). cDNA was synthesized using an All-in-One First Strand cDNA synthesis kit (Accurate Biology). qRT-PCR was performed using the iCycler Sequence Detection System (Bio-Rad) and qPCR SYBR Green Master Mix (Novoprotein). The expression of individual genes was quantified using a standard curve method and normalized to *Actb* expression. The gene-specific primers used in qRT-PCR assays are shown in Supplemental Table 2.

Immunoblotting and immunoprecipitation

Whole-cell and subcellular extracts were prepared, and protein samples were separated by SDS-PAGE, transferred to PVDF membranes (0.45 μm, Millipore), and blocked with 5% BSA, 0.1% Tween-20 in TBS for 1 h. Membranes were incubated overnight with primary antibodies, followed by 1 h incubation with goat anti-rabbit or goat anti-mouse IgG secondary antibodies (1:3,000 dilution). After washing, immunoreactive bands were visualized by chemiluminescence (ECL, Roche Diagnostics) assay according to the manufacturer’s protocol. For the immunoprecipitation assay, cell lysates were cleared by centrifugation, and supernatants were immunoprecipitated with the appropriate antibodies using protein A/G-agarose beads. Samples were then used for immunoblotting analysis with the indicated antibodies.

LC-MS/MS analysis

To identify interacting proteins and TSC2 phosphorylation sites, cells expressing HA-DYRK1A and/or Flag-TSC2 were harvested and lysed in RIPA buffer. Immunoprecipitation was performed at 4 °C for 3 h using either anti-HA or anti-DYKDDDDK (Flag) magnetic beads. Following extensive washing, the bead-bound immune complexes were subjected directly to SDS-PAGE and stained with Coomassie Brilliant Blue. Gel bands corresponding to the proteins of interest were excised, dehydrated in acetonitrile, dried under a vacuum, and subjected to in-gel digestion with trypsin. The digested peptides were then extracted, concentrated, and analyzed by liquid chromatography-tandem mass spectrometry (LC-MS/MS) at the Core Facility for Biomedical Sciences at Xiamen University. The mass spectrometry datasets have been deposited in the iProX repository (Accession numbers: PXD057773 and PXD057775).

Ubiquitination assay

The ubiquitination assay was performed according to an established protocol (5). Briefly, HEK293T cells transfected with the indicated plasmids were lysed in RIPA buffer (50 mM Tris-HCl, pH 7.4, 150 mM NaCl, 1% NP-40, 0.5% sodium deoxycholate, and 1 mM EDTA) containing protease inhibitors and N-ethylmaleimide. After saving some cell extracts for input analysis, the remaining cell extracts were supplemented with SDS to a final concentration of 1% and then boiled at 100°C for 5 min, which dissociated all potential protein complexes under these denaturing conditions. The boiled cell extracts were diluted with RIPA buffer to an SDS concentration of 0.1%, pre-cleared with protein A/G beads, and then incubated with a specific antibody for immunoprecipitation at 4°C overnight. Subsequently, the immunoprecipitated proteins were collected by incubation with protein A/G beads, washed with RIPA buffer, boiled at 100°C for 5 min, and subjected to SDS-PAGE. The immunoprecipitated proteins were analyzed by immunoblotting using anti-ubiquitin or other

indicated antibodies.

Lentivirus infection and transient transfection

For transient expression, transfections were performed with Polyethyleneimine Linear (78PEI25000; BIOHUB) according to the manufacturer's instructions. WT or mutant TSC2 were subcloned into the pcDNA3.3 vector. TSC2 and DYRK1A truncations were generated by assembling corresponding fragments amplified by high-fidelity DNA polymerase 2× Phanta Max Master (P515-01; Vazyme) and then subcloned into pcDNA3.3 vector. The HUWE1 shRNAs (shown in Supplemental Table 3) were constructed into the pLKO.1 retroviral vector. To generate 293T cells stably knocking down HUWE1, lentivirus was produced by co-transfecting 293T cells with psPAX2 (#12260; Addgene), pMD2.G (#12259; Addgene), and pLKO.1 retroviral vectors containing different HUWE1 shRNA sequences. Supernatants were harvested at 48 and 60 h, centrifuged at 6,000 g for 3 min, and then filtered through a 0.22 μm filter. Polybrene (sc-134220; Santa Cruz Technology) was added to facilitate infection. The transduced 293T cells were used for immunoblotting. For lentiviral transduction, WT, S540A, and S540D *Tsc2* mutants were subcloned into a pLV-eGFP vector. Bone marrow cells isolated from *Tsc2*$^{fl/fl}$*Xcr1*-Cre mice were then incubated with the viral supernatant in the presence of 2 μg/mL polybrene. The infection was performed at 1800 rpm and 37°C for 1.5 hours. Following transduction, the medium was replenished with fresh culture medium containing 100 ng/mL Flt3L, and the cells were cultured for 14 days. GFP-positive cells were subsequently sorted as cDC1s based on the gating strategy described above.

DQ-ovalbumin degradation assay

BMDCs were generated from wild-type and *Dyrk1a*$^{fl/fl}$*Cd11c*-Cre mice as described above. BM-cDC1s were generated from wild-type and *Dyrk1a*$^{fl/fl}$*Xcr1*-Cre mice. Generated BMDCs or BM-cDC1s were incubated with 10 μg/mL DQ-OVA for 0, 30, 60, or 120 min. DQ-OVA is a self-quenched OVA conjugate that emits green fluorescence upon hydrolysis by proteases.

Cells were washed with PBS at the indicated time points and analyzed for DQ-OVA release as assessed by positive FITC ($FITC^+$) staining.

Measurement of cytosolic antigen processing

Cytosolic antigen processing by the proteasome was examined according to an established protocol (6). In brief, cDC1 cells were harvested and prepared for electroporation by concentrating 3×10^6 cells in 100 μL of Nucleofector solution supplemented with a mixture of 2 μg DQ-OVA (D12053; Invitrogen) and 2 μg Alexa Fluor 647-OVA (034784; Invitrogen). The cell suspension was then pulsed in a Nucleofector cuvette using a Lonza Nucleofector system (AAB-1001) with program Y-001. Subsequently, the electroporated cells were immediately resuspended in cDC1 culture medium, transferred to a 96-well plate, and cultured at 37°C for the indicated time points. Following the culture period, cells were stained with antibodies targeting cDC1-specific surface markers and analyzed by flow cytometry.

Analysis of phagosomal antigen degradation by flow organellocytometry

To analyze phagosomal antigen degradation, cDC1 cells were washed with PBS and resuspended in medium to a concentration of 1×10^6 cells/ml. We incubated the cells with OVA-coated latex beads (17145; Polysciences) at 16°C for 15 minutes, then quenched the uptake with ice-cold PBS. After two washes, the cells were resuspended in pre-warmed cDC1 medium and incubated at 37°C, 5% CO_2 for the indicated times to allow antigen degradation. The process was halted with ice-cold PBS. For staining, cells were blocked with anti-CD16/32 and sequentially incubated with anti-OVA (C6534; Sigma) and Alexa Fluor 568-conjugated anti-rabbit IgG (A11036; Invitrogen) for 15 minutes each at 4°C. After staining, cells were mechanically lysed with a 22-gauge needle to release organelles. The organelles were fixed/permeabilized with BD Cytofix/Cytoperm buffer, incubated overnight at 4°C with biotin-conjugated LAMP-1 (13-1071-82; Invitrogen) and anti-OVA, and subsequently stained with Alexa Fluor 488-conjugated streptavidin and DyLight 633-conjugated anti-rabbit IgG (35562;

Invitrogen) for 45 minutes. Finally, the organelles were resuspended in PBS for flow cytometry (7).

Measurement of phagosomal pH

Phagosomal pH was measured according to an established protocol (8). Briefly, according to the manufacturer's instructions (Invitrogen), 3 μm latex beads (17145-5; Polysciences) were coated overnight at 4°C with a combination of FITC (pH-sensitive; HY-66019, MCE) and Alexa Fluor 647 (pH-insensitive; A37573, Invitrogen). The following day, the beads were washed and stored in cold PBS. cDC1s were pulsed with conjugated beads for 30 minutes and then extensively washed with cold PBS. The cells were subsequently incubated at 37°C for the indicated time points and immediately analyzed by flow cytometry. The mean fluorescence intensity emission ratio of the two dyes was calculated and compared to a standard curve. This standard curve was generated by resuspending cells that had phagocytosed beads for one hour in solutions mimicking the intralysosomal ionic composition across a pH range (pH 3-8) and containing 0.1% TritonX-100, followed by flow cytometric analysis to determine the emission ratio.

Phagocytosis assay

2×10^5 BMDCs were incubated with FITC-labeled dextran at 37°C for the indicated time points. After incubation, cells were stained with antibodies against DC markers for flow cytometry.

Preparation of tumor cell lysates

B16-F10 and B16-GP33 cells were harvested, washed with PBS, and then resuspended in PBS at 1×10^7 cells/mL. The cells underwent three cycles of freezing and thawing, followed by ultrasonic cell disruption. The effectiveness of cellular disruption was confirmed using trypan blue staining. B16-F10 and B16-GP33 tumor cell lysates were added to cDC1s during

Poly(I:C) treatment at a ratio of two tumor cell equivalents to one cDC1.

LysoTracker staining

For the LysoTracker staining assay, cells were plated on fibronectin-coated coverslips and cultured until they reached 60-80% confluence. The lysosomes were labeled by incubating the cells with 100 nM LysoTracker Red DND-99 (L7528; Invitrogen) in complete culture medium for 1.5 hours under standard culture conditions. Subsequently, the cells were fixed with 4% PFA in PBS for 10 minutes at room temperature, washed, and permeabilized with PBST (PBS containing 0.1% Triton X-100). Nuclei were then counterstained with Hoechst (diluted 1:2000 in PBST) for 10 minutes. Finally, the coverslips were mounted on glass slides using Fluoromount-G mounting medium (00-4958-02; Invitrogen). Images were acquired using a Zeiss LSM 900+ Airyscan 2 confocal microscope with a 63 × oil-immersion objective. LysoTracker signal intensity was quantified using Fiji by analyzing 30 individual cells per genotype in each experiment (9).

Immunofluorescence and confocal microscopy

Cells were plated on fibronectin-coated coverslips and treated as specified for each experiment. Following treatment, the cells were fixed with 4% PFA in PBS for 10 minutes at room temperature. The samples were then washed, permeabilized, and blocked with PBS containing 0.1% Triton X-100 and 3% BSA. Subsequently, the cells were incubated with the indicated primary antibodies (1:200 dilution in PBST), followed by fluorescently conjugated secondary antibodies (Alexa Fluor conjugates at 1:200 in PBST). Nuclei were counterstained with Hoechst, and the coverslips were mounted on glass slides using Fluoromount-G (#00-4958-02, Invitrogen). Images were acquired using a Zeiss LSM 900+ Airyscan 2 confocal microscope with a 63 × oil-immersion objective. The colocalization of mTOR with lysosomes was quantified using ImageJ software, with 30 cells analyzed per group.

Seahorse assays

BMDCs or BM-cDC1s were stimulated with poly(I:C) for 4 hours. Cells were seeded into culture plates at a density of 0.4 × 10^5 cells per well. Extracellular acidification rate and oxygen consumption rate were measured by XF96 Extracellular Flux Analyzer according to the manufacturer's instructions, in the presence of the following compounds: 10 mM glucose, 2 μM oligomycin, 50 mM 2-DG, 4 μM FCCP, and 1 μM rotenone/antimycin A (Agilent Technologies).

TCGA data analysis

For the correlation analysis, gene expression data were obtained from UCSC Xena (http://xena.ucsc.edu/). To analyze the correlation of multiple signatures specific to certain cell types, expression scores for the DYRK1A cDC1, CD4, and CD8 gene signatures were calculated using the Gene Set Variation Analysis (GSVA) algorithm within the R package GSVA (version 1.42.0), based on the signatures provided in Supplemental Table 4. For the survival analysis, TCGA transcriptomic and clinical data for each cancer type were obtained using the R package TCGAbiolinks (version 2.28.3) (10). Survival analysis was then performed using the R package survival. Briefly, gene set enrichment analysis (GSEA) was performed using the ssGSEA algorithm in the R package GSVA to calculate enrichment scores for selected gene signatures (*DYRK1A*, *ITGAX*, *XCR1*, *CLEC9A*) (11). Patients were categorized into *DYRK1A*-cDC1^{high} and *DYRK1A*-cDC1^{low} groups using the surv_cutpoint function in the R package survminer. Kaplan–Meier survival curves were generated with the survfit function. Hazard ratios (HRs) and 95% confidence intervals (CIs) from univariate and multivariate Cox proportional-hazards models were calculated using the coxph function. The log-rank test was applied to assess the statistical significance of differences between groups.

Public single-cell RNAseq data analysis

scRNA-seq data of cDC1s from melanoma were downloaded from GEO using the accession

code GSE221553. The counts matrix was loaded to R (version 4.2.1), and Seurat (version 5.1.0) was used for downstream analyses. Raw UMI counts were normalized and filtered using the Seurat standard pipeline. cDC1 clusters were extracted using the subset function in Seurat. cDC1s were then classified into *DYRK1A*^{high} and *DYRK1A*^{low} groups based on the median expression of DYRK1A. Differentially expressed genes (DEGs) in the DYRK1A^{high} cluster were identified using the FindMarkers function. Gene Ontology (GO) functional enrichment analysis of DEGs with $P < 0.05$ was performed using the R package clusterProfiler (version 4.12.6).

REFERENCES

1. Bar-Peled L, Schweitzer LD, Zoncu R, and Sabatini DM. Ragulator is a GEF for the rag GTPases that signal amino acid levels to mTORC1. *Cell.* 2012;150(6):1196-208.
2. Mayer CT, Ghorbani P, Nandan A, Dudek M, Arnold-Schrauf C, Hesse C, et al. Selective and efficient generation of functional Batf3-dependent CD103+ dendritic cells from mouse bone marrow. *Blood.* 2014;124(20):3081-91.
3. Tseng W, Leong X, and Engleman E. Orthotopic mouse model of colorectal cancer. *J Vis Exp.* 2007(10):484.
4. Tauriello DVF, Palomo-Ponce S, Stork D, Berenguer-Llergo A, Badia-Ramentol J, Iglesias M, et al. TGFbeta drives immune evasion in genetically reconstituted colon cancer metastasis. *Nature.* 2018;554(7693):538-43.
5. Zhu Q, Yu T, Gan S, Wang Y, Pei Y, Zhao Q, et al. TRIM24 facilitates antiviral immunity through mediating K63-linked TRAF3 ubiquitination. *J Exp Med.* 2020;217(7):e20192083.
6. Wang Y, Zhang Q, He T, Wang Y, Lu T, Wang Z, et al. The transcription factor Zeb1 controls homeostasis and function of type 1 conventional dendritic cells. *Nat Commun.* 2023;14(1):6639.
7. Hoffmann E, Pauwels AM, Alloatti A, Kotsias F, and Amigorena S. Analysis of Phagosomal Antigen Degradation by Flow Organellocytometry. *Bio Protoc.* 2016;6(22).
8. Savina A, Vargas P, Guermonprez P, Lennon AM, and Amigorena S. Measuring pH, ROS production, maturation, and degradation in dendritic cell phagosomes using cytofluorometry-based assays. *Methods Mol Biol.* 2010;595:383-402.
9. Gollwitzer P, Grutzmacher N, Wilhelm S, Kummel D, and Demetriades C. A Rag GTPase dimer code defines the regulation of mTORC1 by amino acids. *Nat Cell Biol.* 2022;24(9):1394-406.
10. Colaprico A, Silva TC, Olsen C, Garofano L, Cava C, Garolini D, et al. TCGAbiolinks: an R/Bioconductor package for integrative analysis of TCGA data. *Nucleic Acids Res.* 2016;44(8):e71.
11. Hanzelmann S, Castelo R, and Guinney J. GSVA: gene set variation analysis for microarray and RNA-seq data. *BMC Bioinformatics.* 2013;14:7.

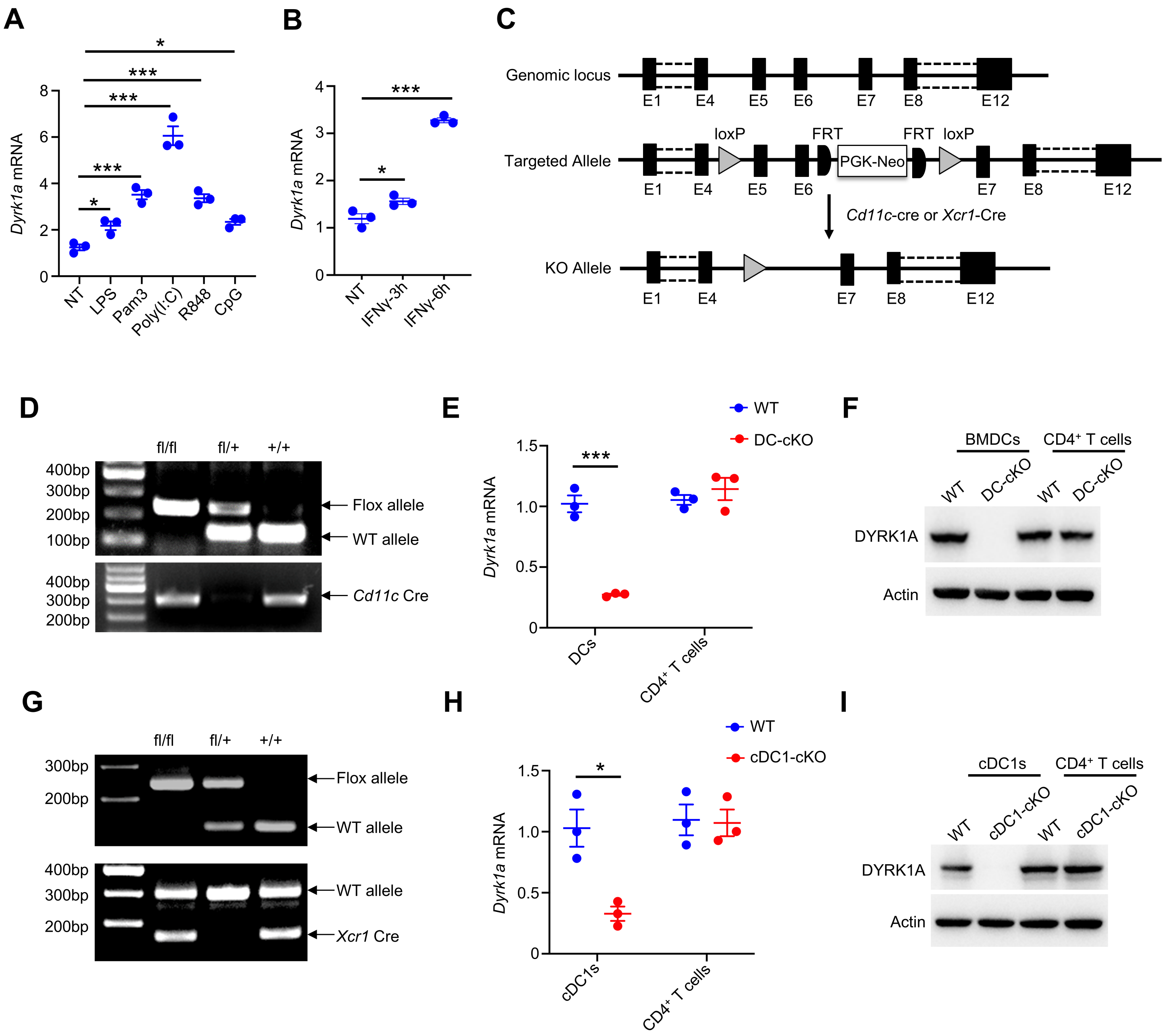

Supplemental Figure 1. Generation of *Dyrk1a* DC-conditional knockout and *Dyrk1a* cDC1-conditional knockout mouse.
(**A-B**) Real-time quantitative reverse-transcription PCR analysis of *Dyrk1a* mRNA levels in bone marrow-derived dendritic cells (BMDCs) stimulated with TLR agonists for 3 hours (**A**) or IFN-γ for the indicated time points (**B**). (**C**) Schematic of DYRK1A gene targeting using an FRT-LoxP vector. Targeted mice were crossed with *Cd11c*-Cre or *Xcr1*-Cre to generate DC-conditional KO (DC-cKO) or cDC1-conditional KO (cDC1-cKO) mice. (**D**) Genomic PCR analysis of *Dyrk1a*$^{+/+}$ (+/+), *Dyrk1a*$^{fl/+}$ (fl/+), and *Dyrk1a*$^{fl/fl}$ (fl/fl) mice crossed with *Cd11c*-Cre mice, showing *Dyrk1a* floxed and wild-type alleles. (**E**) qRT-PCR analysis of *Dyrk1a* mRNA encoded by the deleted exon in *Dyrk1a*-DC-cKO (DC-cKO) mice using sorted DCs and CD4^{+} T cells. (**F**) Immunoblotting analysis of the indicated proteins in whole-cell lysates of wild-type and *Dyrk1a*-DC-cKO BMDCs and sorted CD4^{+} T cells. (**G**) Genomic PCR analysis of *Dyrk1a*$^{+/+}$ (+/+), *Dyrk1a*$^{fl/+}$ (fl/+), and *Dyrk1a*$^{fl/fl}$ (fl/fl) mice crossed with *Xcr1*-Cre mice, showing *Dyrk1a* floxed and wild-type alleles. (**H**) qRT-PCR analysis of *Dyrk1a* mRNA encoded by the deleted exon in *Dyrk1a*-cDC1-cKO (cDC1-cKO) mice using sorted cDC1s and CD4^{+} T cells. (**I**) Immunoblotting analysis of the indicated proteins in whole-cell lysates of wild-type and *Dyrk1a*-cDC1-cKO BM-cDC1s and sorted CD4^{+} T cells. P values were determined using one-way ANOVA (**A**, **B**) and a two-tailed unpaired Student's *t*-test (**E**, **H**). *$P < 0.05$; **$P < 0.01$; ***$P < 0.001$.

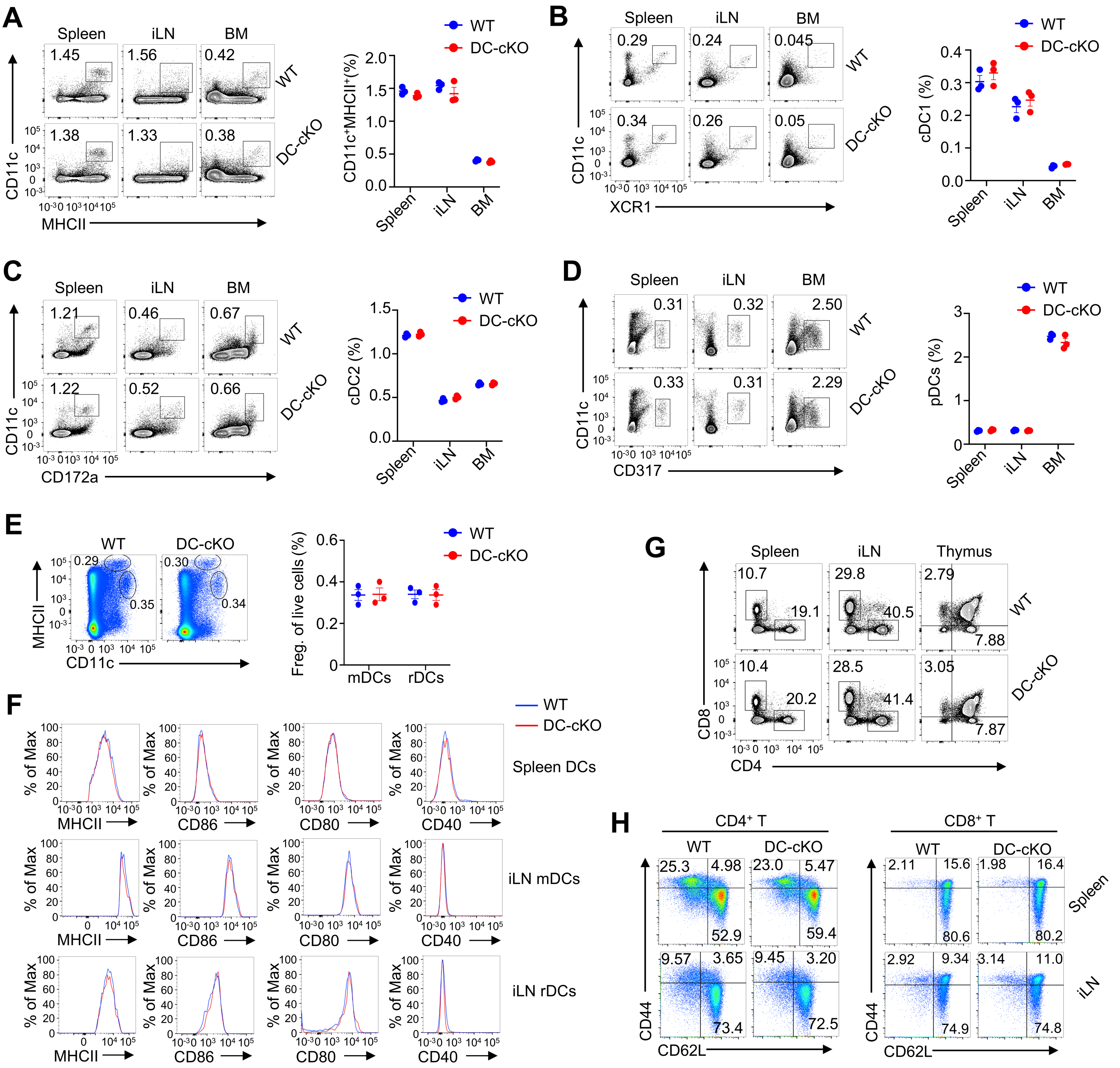

Supplemental Figure 2. DC-specific *Dyrk1a* deficiency does not influence DC and T cell homeostasis.
(**A**-**D**) Flow cytometric analysis of the CD11c$^+$MHCII$^+$ DC population (**A**), CD11c$^+$XCR1$^+$ cDC1 population (**B**), CD11c$^+$CD172a$^+$ cDC2 population (**C**), and CD11c$^+$CD317$^+$ pDC population (**D**) from spleen, inguinal lymph nodes (iLN), and bone marrow (BM) of WT and *Dyrk1a*-DC-cKO mice. N=3 mice per group. (**E**) Flow cytometric analysis of migratory DC (CD11c^{int} MHCII^{high}) and resident DC (CD11c$^+$MHCII$^+$) in the iLN of wild-type and *Dyrk1a*-DC-cKO mice. N=3 mice per group. (**F**) Flow cytometric analysis of MHCII, CD80, CD86, and CD40 in splenic DCs, migratory DCs, and resident DCs from inguinal lymph nodes. (**G**) Flow cytometric analysis of CD4$^+$ and CD8$^+$ T cells from spleen, inguinal lymph nodes (iLN), and thymus of WT and *Dyrk1a*-DC-cKO mice. (**H**) Flow cytometric analysis of the frequency of naive (CD44^{lo}CD62L^{hi}), effector memory (CD44^{hi}CD62L^{lo}), and central memory (CD44^{hi}CD62L^{hi}) CD4$^+$ and CD8$^+$ T cells in the spleen and iLNs from WT and *Dyrk1a*-DC-cKO mice. Data are representative of three independent experiments. Summary data are shown as the mean ± SEM.

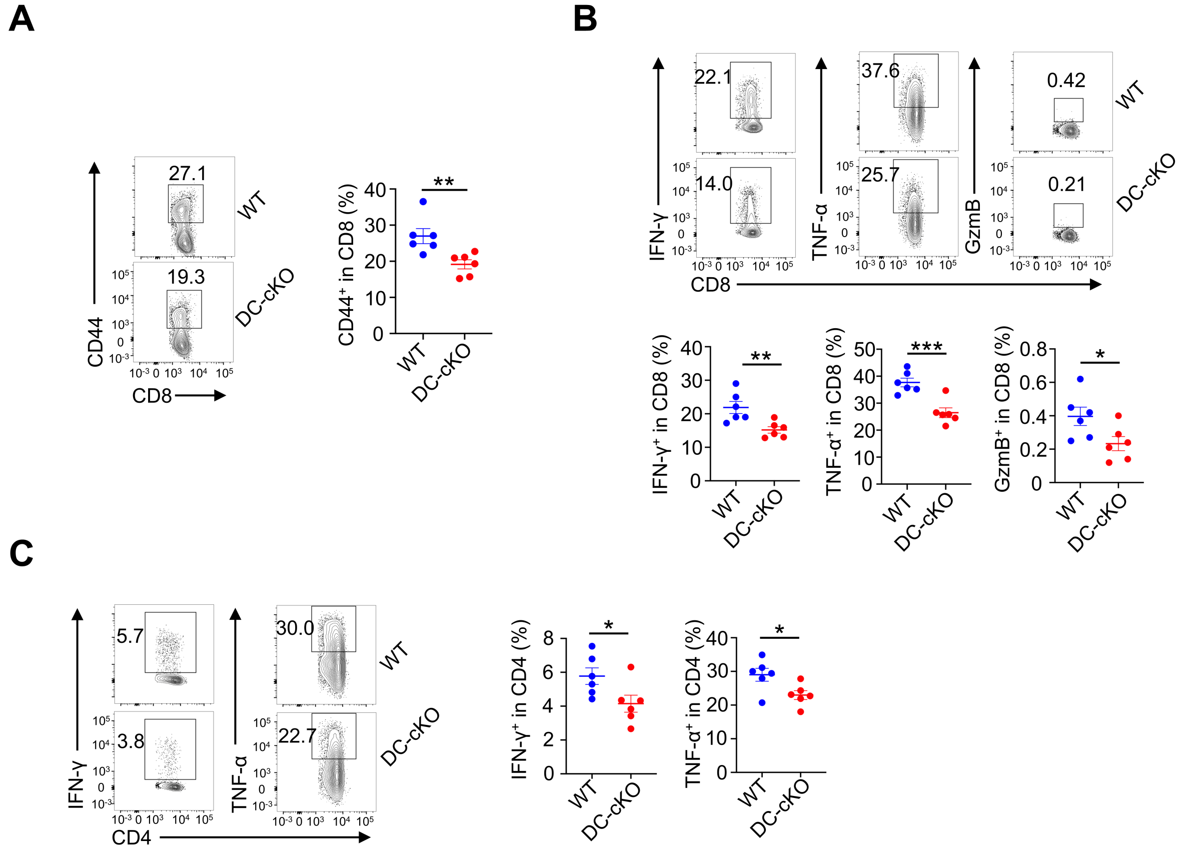

Supplemental Figure 3. DYRK1A deletion in DCs impaired T cell responses in the tumor-draining lymph nodes.
(**A**) CD44 expression on $CD8^+$ T cells from tumor-draining lymph nodes, shown in a representative flow cytometry plot (left panel) and summary graph (right panel). (**B** and **C**) Flow cytometric analysis of the frequency of IFN-γ-, TNF-α-, Granzyme B-producing $CD8^+$ T cells (**B**) and $CD4^+$ T cells (**C**) in the tumor-draining lymph nodes of wild-type and *Dyrk1a*-DC-cKO mice. Data are presented as a representative flow cytometry plot and a summary graph. N=6 per group. Data are representative of three independent experiments. Summary data are shown as the mean ± SEM. P values were determined using a two-tailed unpaired Student's *t*-test. *$P < 0.05$; **$P < 0.01$; ***$P < 0.001$.

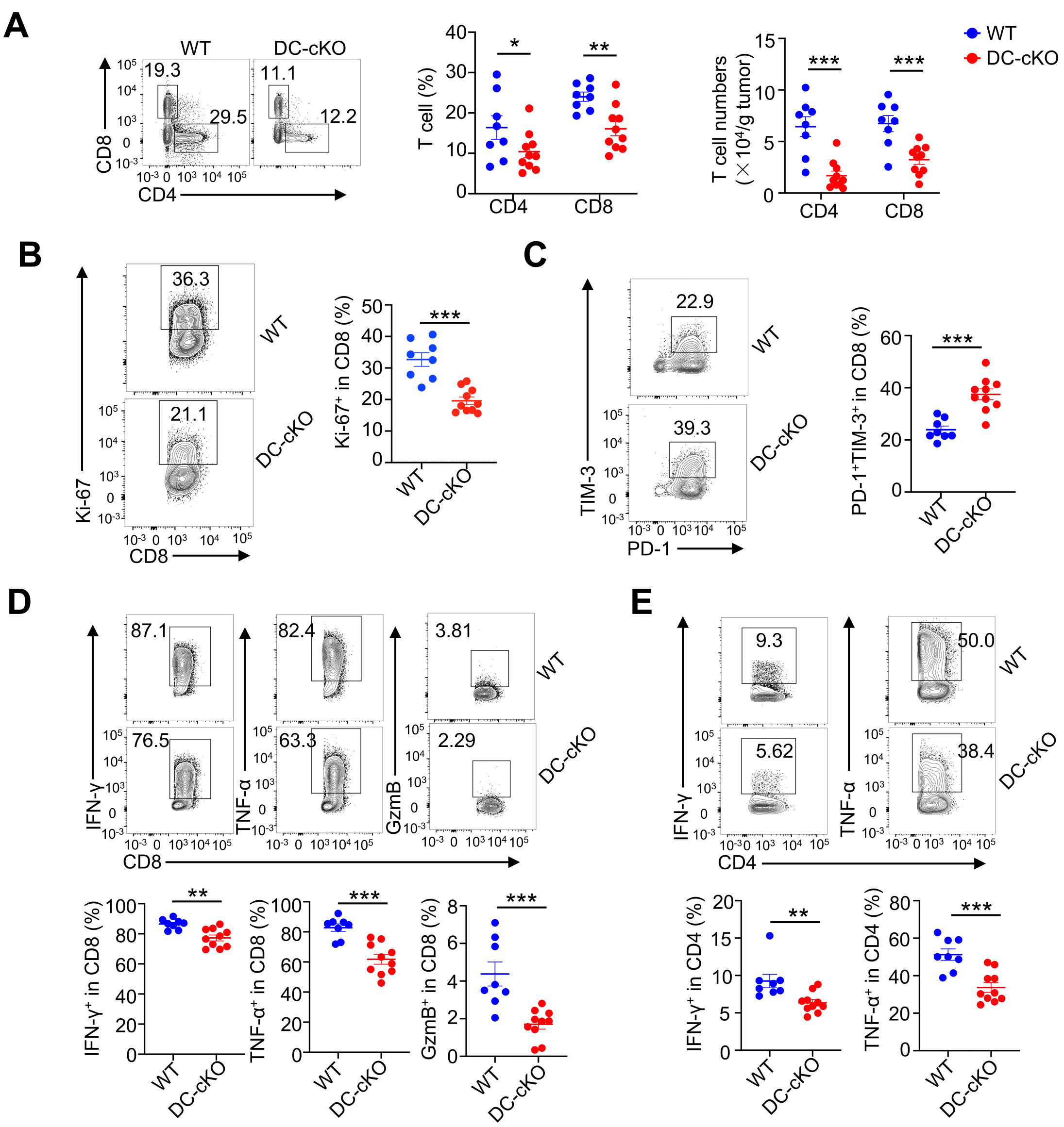

Supplemental Figure 4. Ablation of DYRK1A in DCs impairs antitumor immunity in the MC38 colon cancer model.
(**A**) Flow cytometric analysis of the frequency and absolute cell number of tumor-infiltrating $CD4^+$ and $CD8^+$ T cells from tumor-bearing WT and *Dyrk1a*-DC-cKO mice. Data are presented as a representative flow cytometry plot (left) and a summary graph (right). (**B** and **C**) Flow cytometric analysis on Ki-67 (**B**) and TIM-3, PD-1 (**C**) levels of tumor-infiltrating $CD8^+$ T cells. Data are presented as a representative flow cytometry plot (left panel) and a summary graph (right panel). (**D** and **E**) Flow cytometric analysis of the frequency of IFN-γ-, TNF-α-, Granzyme B-producing $CD8^+$ T cells (**D**) and $CD4^+$ T cells (**E**) in the tumors of wild-type and *Dyrk1a*-DC-cKO mice. Data are presented as a representative flow cytometry plot (upper panel) and a summary graph (lower panel). Wild-type mice: N=8; *Dyrk1a*-DC-cKO mice: N=10. Data are representative of three independent experiments. Summary data are shown as the mean ± SEM. P values were determined using a two-tailed unpaired Student's *t*-test. *$P < 0.05$; **$P < 0.01$; ***$P < 0.001$.

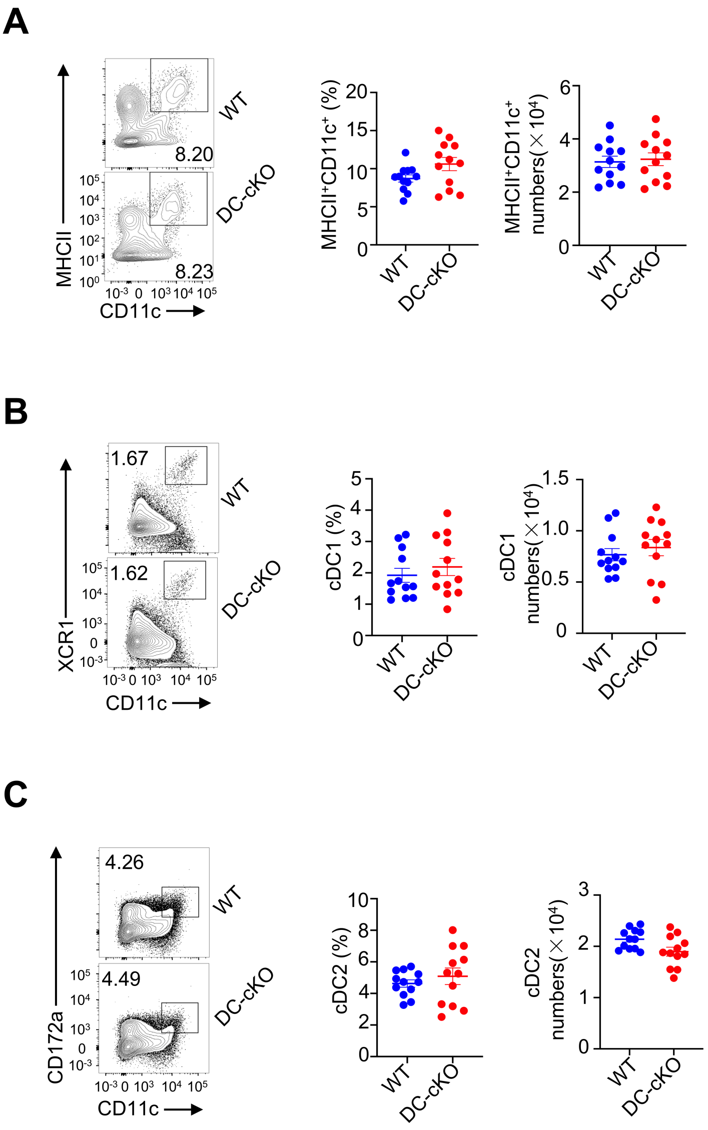

Supplemental Figure 5. DYRK1A deficiency in DCs does not affect tumoral DC subsets.
Flow cytometric analysis of the frequency and absolute cell number of tumor-infiltrating MHCII$^+$CD11c$^+$ DCs (**A**), CD11c$^+$XCR1$^+$ cDC1 (**B**), and CD11c$^+$CD172a$^+$ cDC2 population (**C**). Data are presented as a representative flow cytometry plot (left) and a summary graph (right). N=12 mice per group. Data are representative of three independent experiments. Summary data are shown as the mean ± SEM.

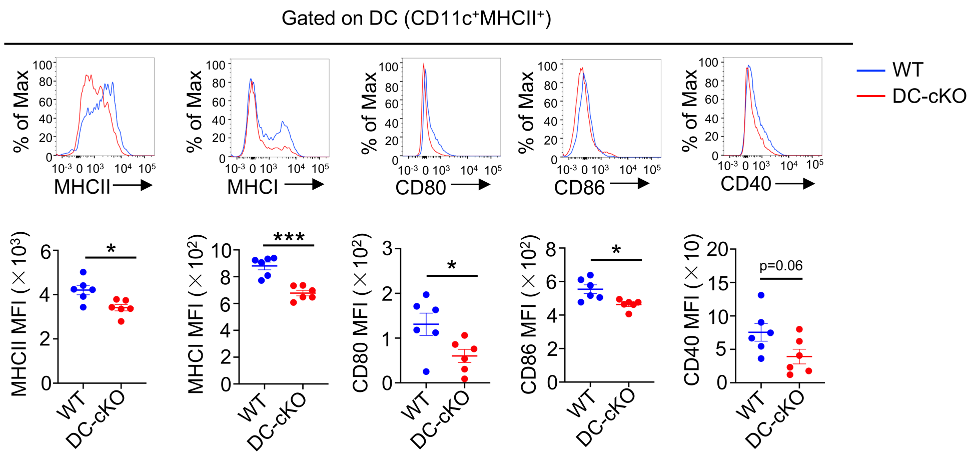

Supplemental Figure 6. DYRK1A deficiency decreased the expression levels of MHC and co-stimulatory molecules of DCs in the tumor-draining lymph nodes.
Flow cytometric analysis of MHCI, MHCII, CD80, CD86, and CD40 levels of MHCII$^+$CD11c$^+$ DCs in the tumor-draining lymph nodes from wild-type and *Dyrk1a*-DC-cKO mice. N=6 per group. Data are representative of three independent experiments. Summary data are shown as the mean ± SEM. P values were determined using a two-tailed unpaired Student's *t*-test. *$P < 0.05$; ***$P < 0.001$.

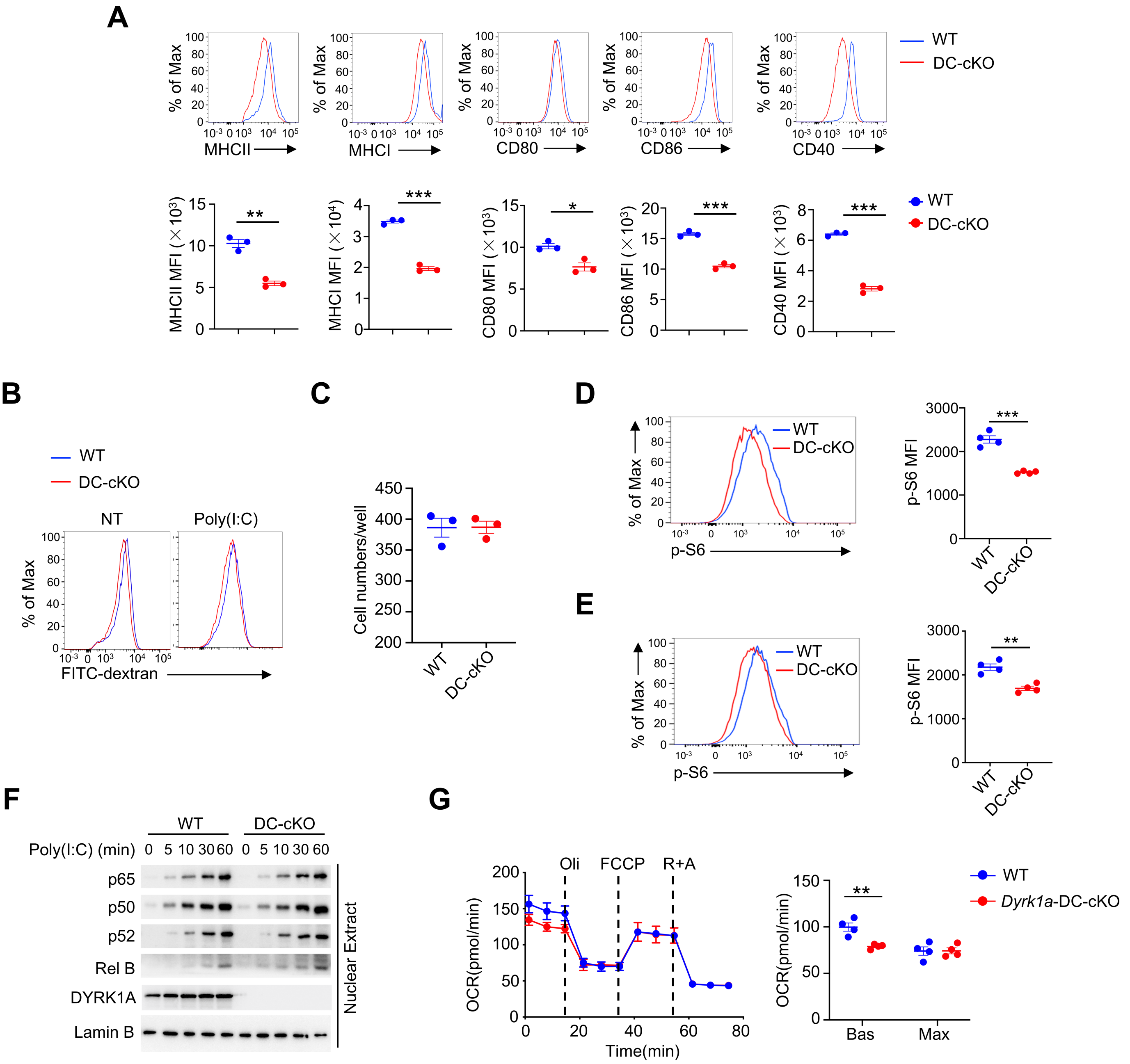

Supplemental Figure 7. DYRK1A regulates the activation and maturation of DCs.

(**A**) Flow cytometric analysis of MHCI, MHCII, CD80, CD86, and CD40 levels of wild-type and DYRK1A-deficient BMDCs stimulated with Poly(I:C) for 12 hours. N=3 per group. Data are shown as a representative flow cytometry plot (upper panel) and a summary graph (lower panel). (**B**) BMDCs were incubated with FITC-dextran for 1 hour, and the phagocytosis ability was measured by flow cytometry. (**C**) Wild-type and *Dyrk1a*-deficient DCs were cultured in the upper chambers of 24-well transwell plates. The lower chambers contained complete medium with rCCL21 (100 ng/ml). After 24 h of culture, the cells in the lower chambers were collected for counting. (**D-E**) Wild-type and *Dyrk1a*-deficient DCs were stimulated with IFN-γ for 24 hours (**D**) or co-cultured with $CD8^+$ T cells for 72 hours (**E**), and the p-S6 levels of DCs were analyzed by flow cytometry. N=4 per group. (**F**) Immunoblot analysis of the indicated proteins in the nuclear extracts of wild-type or *Dyrk1a*-DC-cKO BMDCs stimulated with Poly(I:C). (**G**) Oxygen consumption rates (OCR) of DCs stimulated with Poly(I:C) for 4 hours under basal conditions (Bas) or at maximum (Max) with the addition of oligomycin (Oli), the mitochondrial uncoupler FCCP, and rotenone plus antimycin A (R+A). N=4 per group. Data are representative of three independent experiments. Summary data are shown as the mean ± SEM. P values were determined using a two-tailed unpaired Student's *t*-test. *$P < 0.05$; **$P < 0.01$; ***$P < 0.001$.

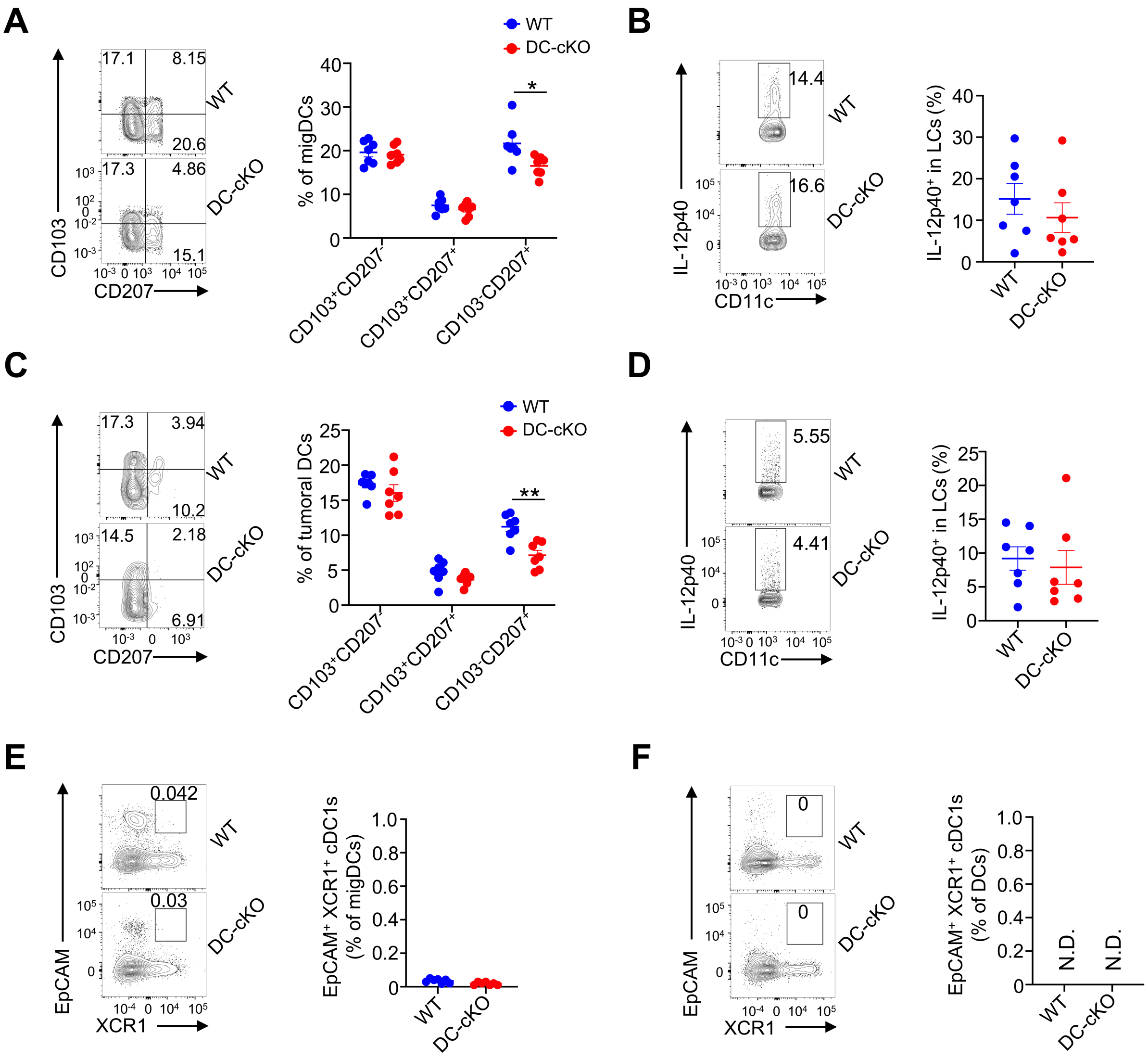

Supplemental Figure 8. The effect of DYRK1A in modulating LCs and EpCAM⁺ cDC1s.

(**A**) Flow cytometric analysis of Langerin (CD207) and CD103 expression on migratory DCs in draining LNs of tumor-bearing WT and *Dyrk1a*-DC-cKO mice. Populations in plots are gated on migratory DCs in draining LNs. (**B**) Flow cytometric analysis of intracellular IL-12 levels of LCs in the draining LNs from tumor-bearing WT and *Dyrk1a*-DC-cKO mice. (**C**) Flow cytometric analysis of Langerin (CD207) and CD103 expression on tumoral DCs of tumor-bearing WT and *Dyrk1a*-DC-cKO mice. (**D**) Flow cytometric analysis of intracellular IL-12 levels of LCs in the tumor from tumor-bearing WT and *Dyrk1a*-DC-cKO mice. (**E**-**F**) Flow cytometric analysis of the frequency of EpCAM$^+$ cDC1 in total DCs from draining LNs (**E**) and tumor (**F**) of WT and *Dyrk1a*-cDC1-cKO mice. N=7 mice per group. Data are representative of three independent experiments. Summary data are shown as the mean ± SEM. P values were determined using a two-tailed unpaired Student's *t*-test. $^*P < 0.05$; $^{**}P < 0.01$.

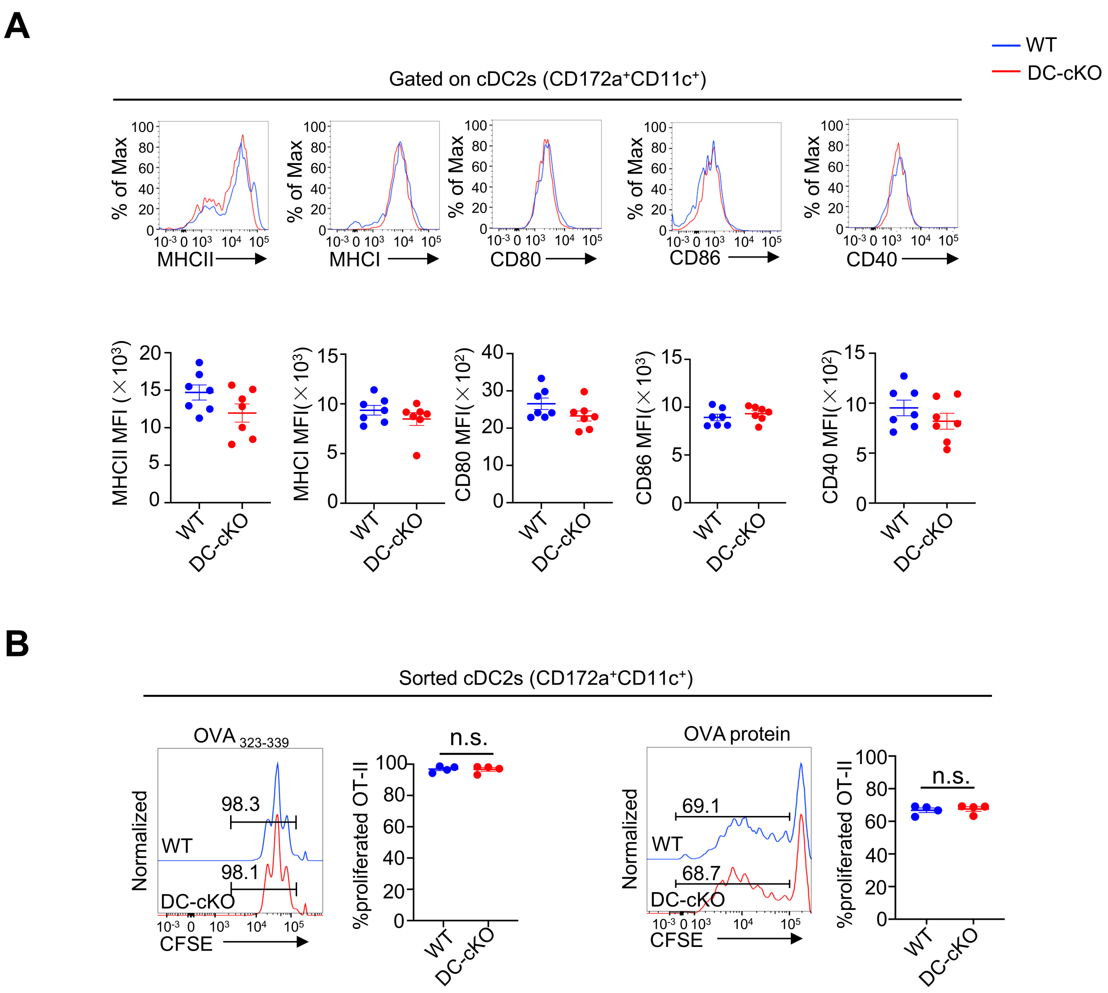

Supplemental Figure 9. DYRK1A ablation does not perturb cDC2 function, with normal expression of MHC and costimulatory molecules and intact T cell priming ability. (**A**) Flow cytometric analysis of MHCI, MHCII, CD80, CD86, and CD40 levels of tumor-infiltrating cDC2s (CD172a⁺CD11c⁺). Data are presented as a representative flow cytometry plot (upper panel) and summary graphs (lower panel). N=7 mice per group. (**B**) Flow cytometric analysis to examine the proliferation of CFSE-labeled OT-II T cells incubated with WT or *Dyrk1a*-deficient cDC2s pulsed with $OVA_{323\text{-}339}$ peptide and full-length OVA protein. Data are presented as a representative flow cytometry plot (left panel) and summary graphs (right panel). N=4 per group. Data are representative of three independent experiments. Summary data are shown as the mean ± SEM.

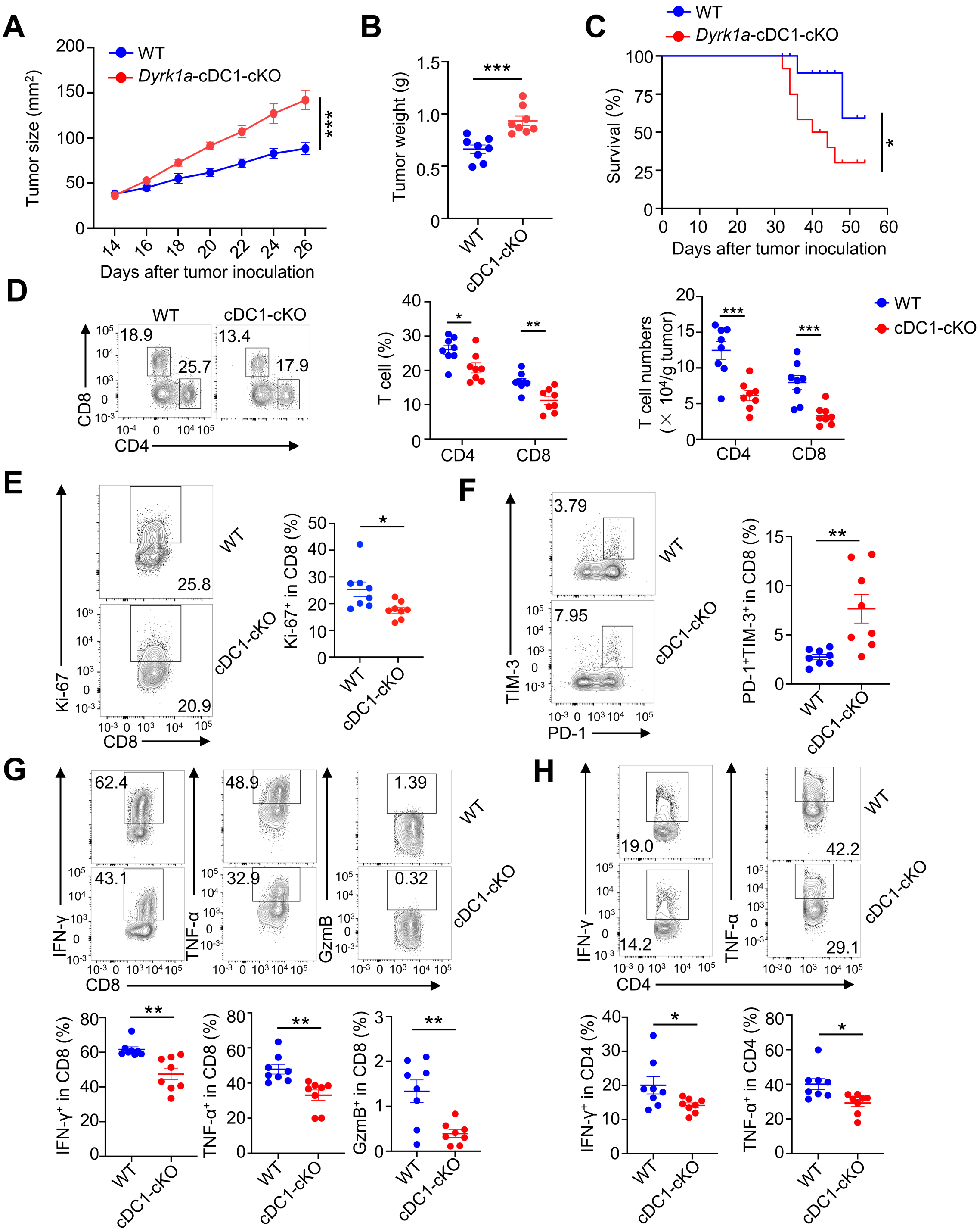

Supplemental Figure 10. DYRK1A deficiency in cDC1s impairs antitumor immunity in the MB49 Bladder cancer model. (**A**-**C**) Tumor growth curve (**A**), tumor weight (**B**), and survival curve (**C**) of wild-type and *Dyrk1a*-cDC1-cKO mice subcutaneously injected with MB49 bladder cancer cells. N=8 mice per group. (**D**) Flow cytometric analysis of the frequency and absolute cell number of tumor-infiltrating $CD4^+$ and $CD8^+$ T cells. Data are presented as a representative flow cytometry plot (left) and a summary graph (right). (**E**-**F**) Flow cytometric analysis of Ki-67 (**E**) and TIM-3, PD-1 (**F**) levels of tumor-infiltrating $CD8^+$ T cells. Data are presented as a representative flow cytometry plot (left panel) and a summary graph (right panel). (**G**-**H**) Flow cytometric analysis of the frequency of IFN-γ-, TNF-α-, Granzyme B-producing $CD8^+$ T cells (**G**) and $CD4^+$ T cells (**H**) in the tumors of wild-type and *Dyrk1a*-cDC1-cKO mice. Data are presented as a representative flow cytometry plot (upper panel) and a summary graph (lower panel). N=8 mice per group. Data are representative of three independent experiments. Summary data are shown as the mean ± SEM. P values were determined using a two-tailed unpaired Student's *t*-test (**B**, **D**-**H**), two-sided log-rank Mantel-Cox test (**C**), or two-way ANOVA with Bonferroni correction (**A**). $^*P < 0.05$; $^{**}P < 0.01$; $^{***}P < 0.001$.

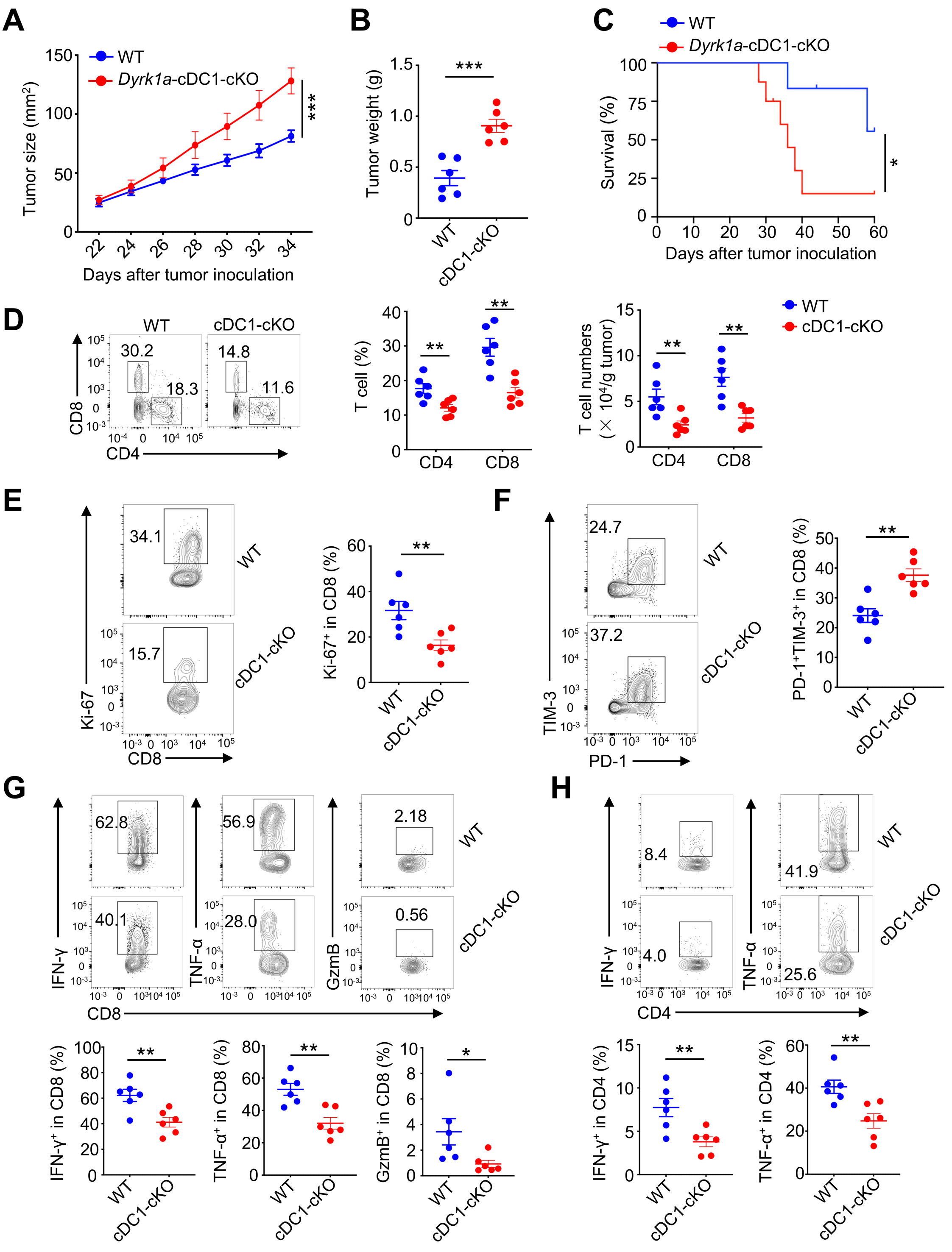

Supplemental Figure 11. DYRK1A deficiency in cDC1s compromised antitumor immunity in the MC38 colon cancer model. (**A**-**C**) Tumor growth curve (**A**), tumor weight (**B**), and survival curve (**C**) of wild-type and *Dyrk1a*-cDC1-cKO mice subcutaneously injected with MC38 colon cancer cells. N=6 mice per group. (**D**) Flow cytometric analysis of the frequency and absolute cell number of tumor-infiltrating CD4⁺ and CD8⁺ T cells. Data are presented as a representative flow cytometry plot (left) and a summary graph (right). (**E**-**F**) Flow cytometric analysis of Ki-67 (**E**) and TIM-3, PD-1 (**F**) levels of tumor-infiltrating CD8⁺ T cells. Data are presented as a representative flow cytometry plot (left panel) and a summary graph (right panel). (**G**-**H**) Flow cytometric analysis of the frequency of IFN-γ-, TNF-α-, Granzyme B-producing CD8⁺ T cells (**G**) and CD4⁺ T cells (**H**) in the tumors of wild-type and *Dyrk1a*-cDC1-cKO mice. Data are presented as a representative flow cytometry plot (upper panel) and a summary graph (lower panel). N=6 mice per group. Data are representative of three independent experiments. Summary data are shown as the mean ± SEM. P values were determined using a two-tailed unpaired Student's *t*-test (**B**, **D**-**H**), two-sided log-rank Mantel-Cox test (**C**), or two-way ANOVA with Bonferroni correction (**A**). $^*P < 0.05$; $^{**}P < 0.01$; $^{***}P < 0.001$.

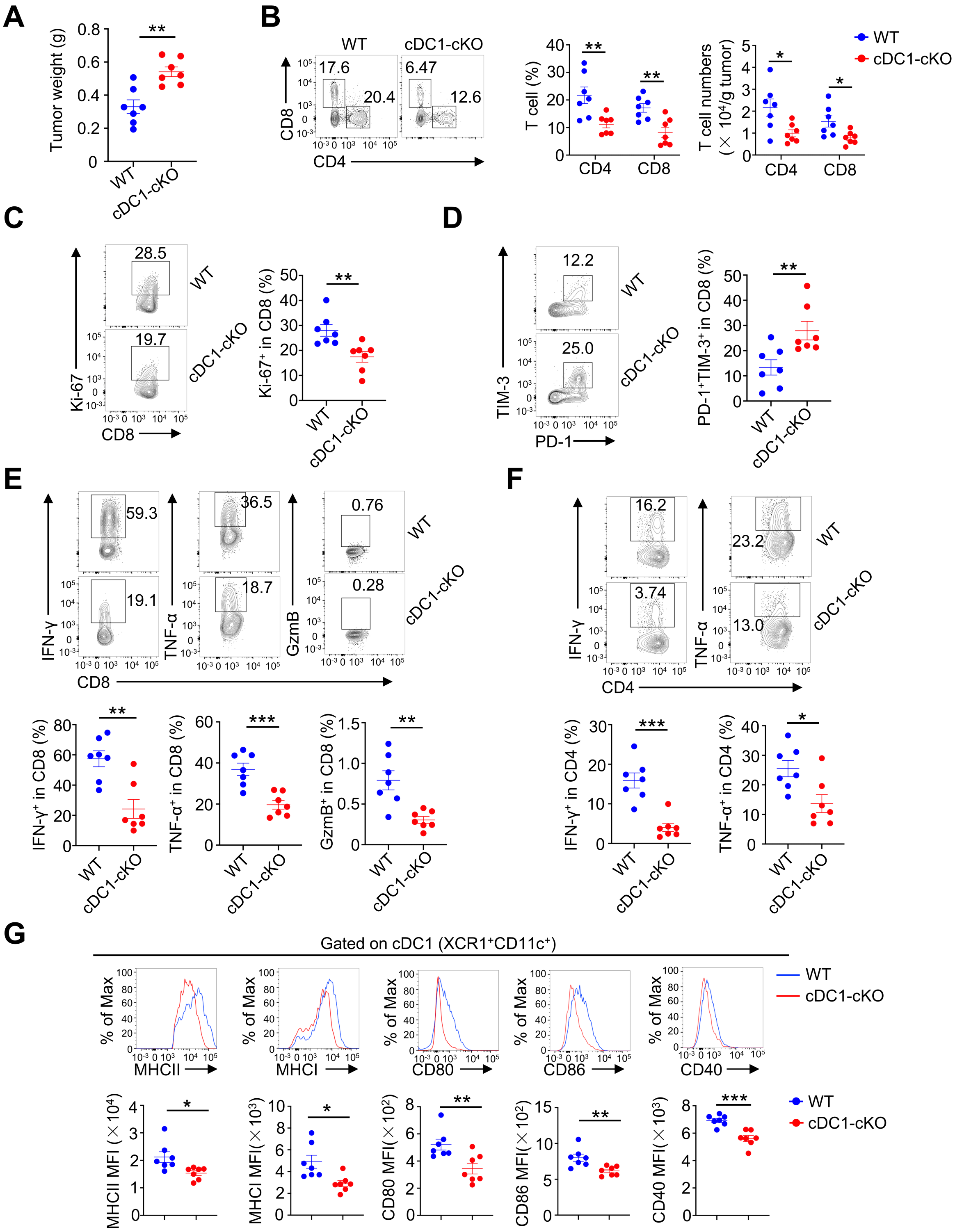

Supplemental Figure 12. Loss of DYRK1A in cDC1 blunted antitumor immunity in an orthotopic tumor model. We established an orthotopic tumor model by inoculating MC-38 colon adenocarcinoma cells into the mouse colon. (**A**) Tumor weight of wild-type and *Dyrk1a*-cDC1-cKO mice at day 30 post MC-38 inoculation. N=7 mice per group. (**B**) Flow cytometric analysis of the frequency and absolute cell number of tumor-infiltrating $CD4^+$ and $CD8^+$ T cells. N=7 mice per group. Data are presented as a representative flow cytometry plot (left) and a summary graph (right). (**C**-**D**) Flow cytometric analysis of Ki-67 (**C**) and TIM-3, PD-1 (**D**) levels of tumor-infiltrating $CD8^+$ T cells. N=7 mice per group. Data are presented as a representative flow cytometry plot (left panel) and a summary graph (right panel). (**E**-**F**) Flow cytometric analysis of the frequency of IFN-γ-, TNF-α-, Granzyme B-producing $CD8^+$ T cells (**E**) and $CD4^+$ T cells (**F**) in the tumors of wild-type and *Dyrk1a*-cDC1-cKO mice. Data are presented as a representative flow cytometry plot (upper panel) and a summary graph (lower panel). N=7 mice per group. (**G**) Flow cytometric analysis of MHCII, MHCI, CD86, CD80, and CD40 levels of tumor-infiltrating cDC1s ($CD11c^+XCR1^+$). N=7 mice per group. Data are representative of three independent experiments. Summary data are shown as the mean ± SEM. P values were determined using a two-tailed unpaired Student's *t*-test. *$P < 0.05$; **$P < 0.01$; ***$P < 0.001$.

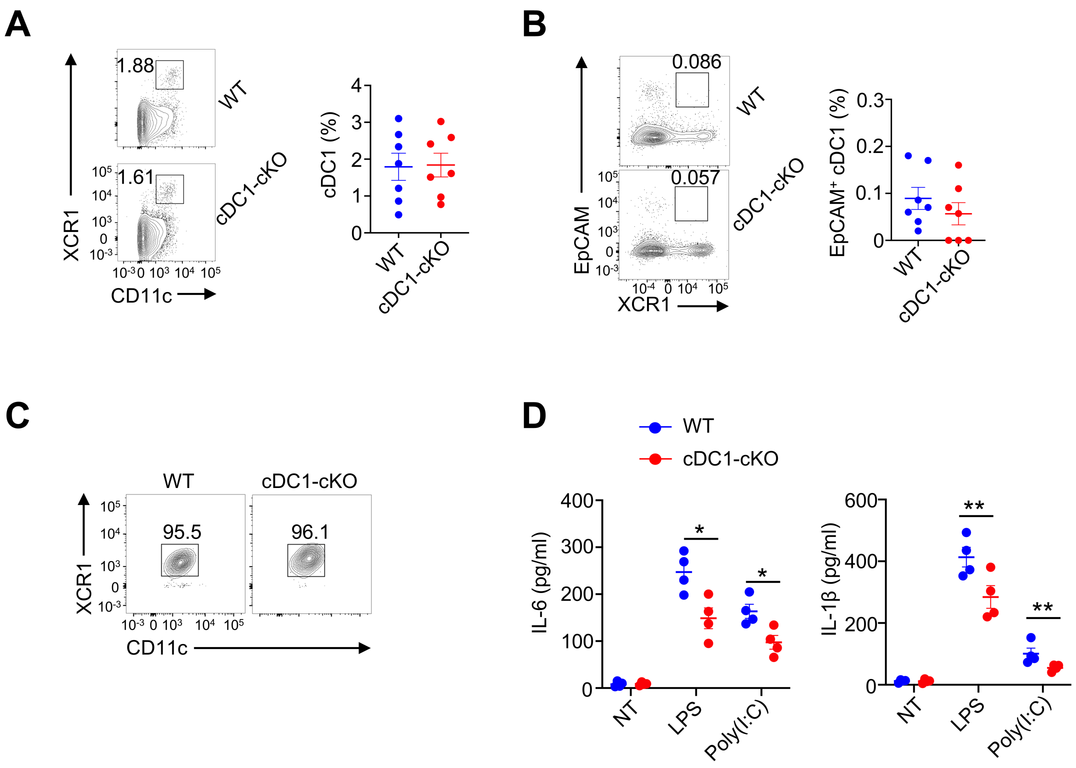

Supplemental Figure 13. DYRK1A deficiency decreases IL-6 and IL-1β levels of cDC1s.
(**A**) Flow cytometric analysis of the frequency of tumor-infiltrating CD11c$^+$XCR1$^+$ cDC1 from tumor-bearing WT and *Dyrk1a*-cDC1-cKO mice. N=7 mice per group. (**B**) Flow cytometric analysis of the frequency of tumor-infiltrating EpCAM$^+$ cDC1 in total DCs from WT and *Dyrk1a*-cDC1-cKO mice. N=7 mice per group. (**C**) Flow cytometric analysis of the purity of *in vitro* cultured BM-cDC1s. (**D**) ELISA analysis of IL-6 and IL-1β levels using WT and *Dyrk1a*-deficient BM-cDC1s, either non-treated (NT) or stimulated with LPS or Poly(I:C) for 24 h. N=4 mice per group. Data are representative of three independent experiments. Summary data are shown as the mean ± SEM. P values were determined using a two-tailed unpaired Student's *t*-test. *$P < 0.05$; **$P < 0.01$.

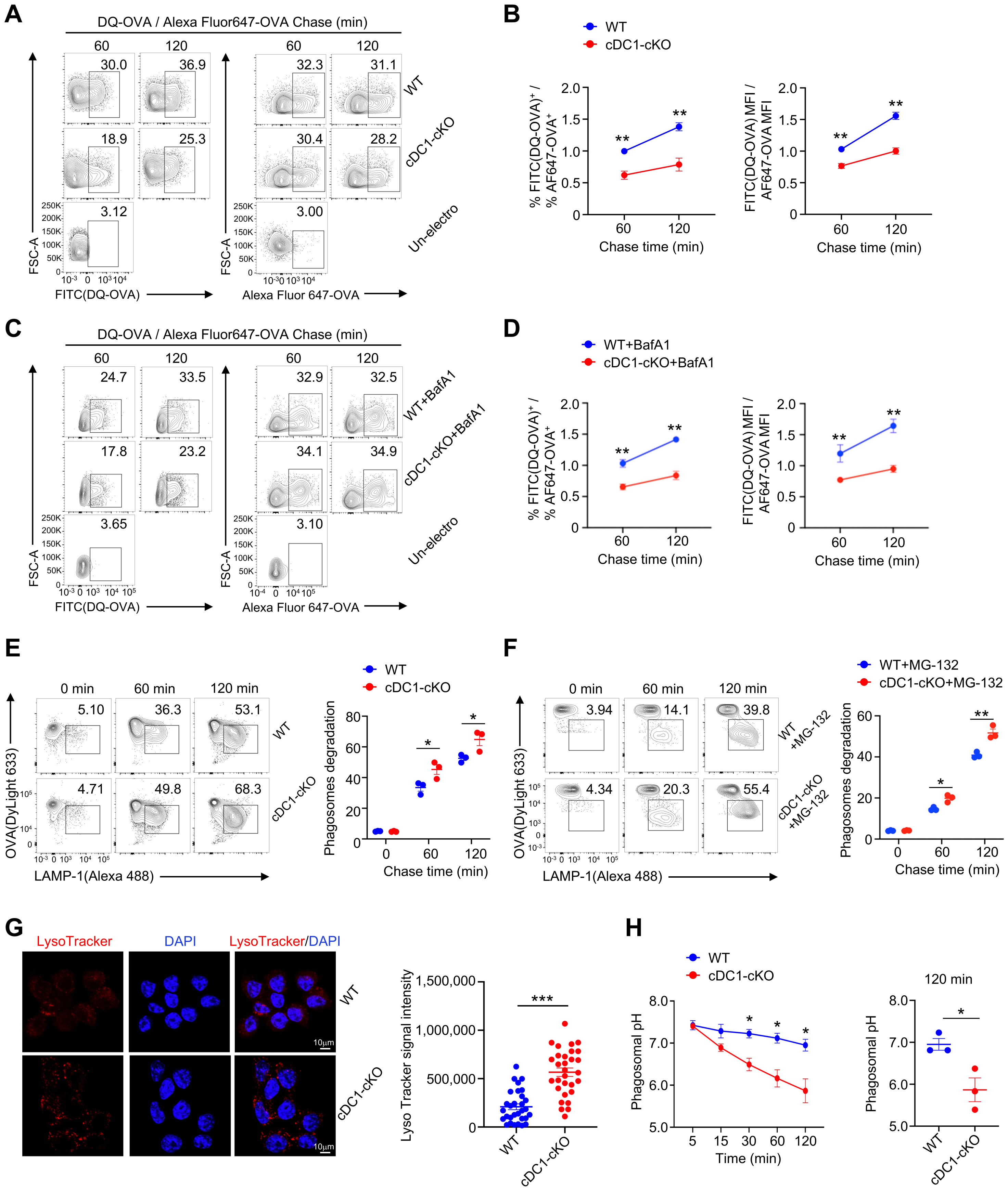
A
DQ-OVA / Alexa Fluor647-OVA Chase (min)
60
120
30.0
36.9
32.3
31.1
WT
18.9
25.3
30.4
28.2
cDC1-cKO
3.12
3.00
Un-electro
FSC-A
FITC(DQ-OVA)
Alexa Fluor 647-OVA
B
WT
cDC1-cKO
% FITC(DQ-OVA)+ / % AF647-OVA+
FITC(DQ-OVA) MFI / AF647-OVA MFI
Chase time (min)
C
DQ-OVA / Alexa Fluor647-OVA Chase (min)
24.7
33.5
32.9
32.5
WT+BafA1
17.8
23.2
34.1
34.9
cDC1-cKO+BafA1
3.65
3.10
Un-electro
D
WT+BafA1
cDC1-cKO+BafA1
E
0 min
60 min
120 min
5.10
36.3
53.1
4.71
49.8
68.3
OVA(DyLight 633)
LAMP-1(Alexa 488)
Phagosomes degradation
Chase time (min)
F
WT+MG-132
cDC1-cKO+MG-132
3.94
14.1
39.8
4.34
20.3
55.4
G
LysoTracker
DAPI
LysoTracker/DAPI
10μm
Lyso Tracker signal intensity
H
Phagosomal pH
Time (min)
120 min

I

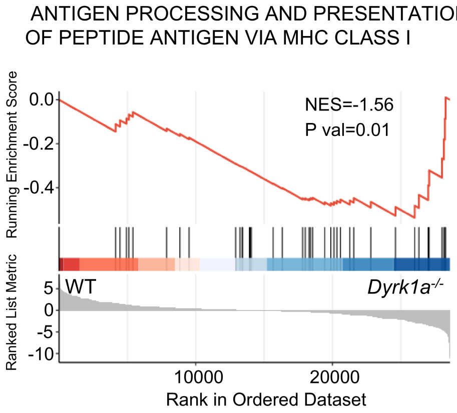

Supplemental Figure 14. DYRK1A deficiency impaired proteasomal degradation capacity, while accelerating lysosomal antigen degradation, in cDC1s. (**A**) To examine the proteasome-mediated antigen degradation, equal amounts of DQ-OVA (2 μg) and Alexa Fluor 647-OVA (2 μg) were introduced into the cytosols of WT and *Dyrk1a*-deficient cDC1 by electroporation. Cytosolic antigen degradation, mainly mediated by the proteasome, was measured by flow cytometry after chasing for the indicated time points. N=3 per group. (**B**) The proteasomal degradation was quantified as the ratio of FITC (DQ-OVA)$^+$ cells to Alexa Fluor 647$^+$ cells (Left panel) or the ratio of MFI of FITC (DQ-OVA) fluorescence to MFI of Alexa Fluor 647 fluorescence (Right panel). N=3 per group. (**C-D**) To exclude potential lysosomal-mediated antigen degradation, we analyzed proteasome-mediated antigen degradation in WT and *Dyrk1a*-deficient cDC1s in the presence of BafA1 (100 μM), followed by electroporation with equal amounts of DQ-OVA and Alexa Fluor 647-OVA. Proteasomal degradation was measured by flow cytometry after chasing for the indicated time points (**C**), and quantified by the ratio of FITC (DQ-OVA)$^+$ cells to Alexa Fluor 647$^+$ cells (Left panel) or the ratio of MFI of FITC (DQ-OVA) fluorescence to MFI of Alexa Fluor 647 fluorescence (Right panel) (**D**). (**E**) Phagosomes were analyzed for degradation of OVA and acquisition of LAMP-1 by gating on the mature OVA (DyLight 633)-negative, and LAMP-1 (Alexa 488)-positive population. N=3 per group. (**F**) To exclude potential proteasomal-mediated antigen degradation, we examined lysosomal-mediated antigen degradation in WT and *Dyrk1a*-deficient cDC1s in the presence of MG-132 (10 μM). Phagosomes were analyzed for degradation of OVA and acquisition of LAMP-1 by gating on the mature OVA (DyLight 633)-negative, and LAMP-1 (Alexa 488)-positive population. N=3 per group. (**G**) LysoTracker staining in wild-type and *Dyrk1a*-deficient cDC1s was analyzed by confocal microscopy (Left panel), and LysoTracker signal intensity was quantified from 30 individual cells. Scale bars, 10 μm. (**H**) To evaluate phagosome acidification, we incubated wild-type and *Dyrk1a*-deficient cDC1s with OVA-coated latex beads conjugated to either FITC (pH-sensitive) or Alexa Fluor 647 (pH-insensitive) and analyzed them by flow cytometry over time. The phagosome pH of cDC1 was calculated as the ratio of the fluorescence intensity of FITC to that of Alexa Fluor 647. N=3 per group. (**I**) Gene set enrichment analysis of the antigen processing and presentation of peptide antigen via MHC class I in the freshly isolated tumoral cDC1s from wild-type and *Dyrk1a*-cDC1-cKO mice. Data are representative of three independent experiments. Summary data are shown as the mean ± SEM. P values were determined using a two-tailed unpaired Student's *t*-test. **P* < 0.05; ***P* < 0.01; ****P* < 0.001.

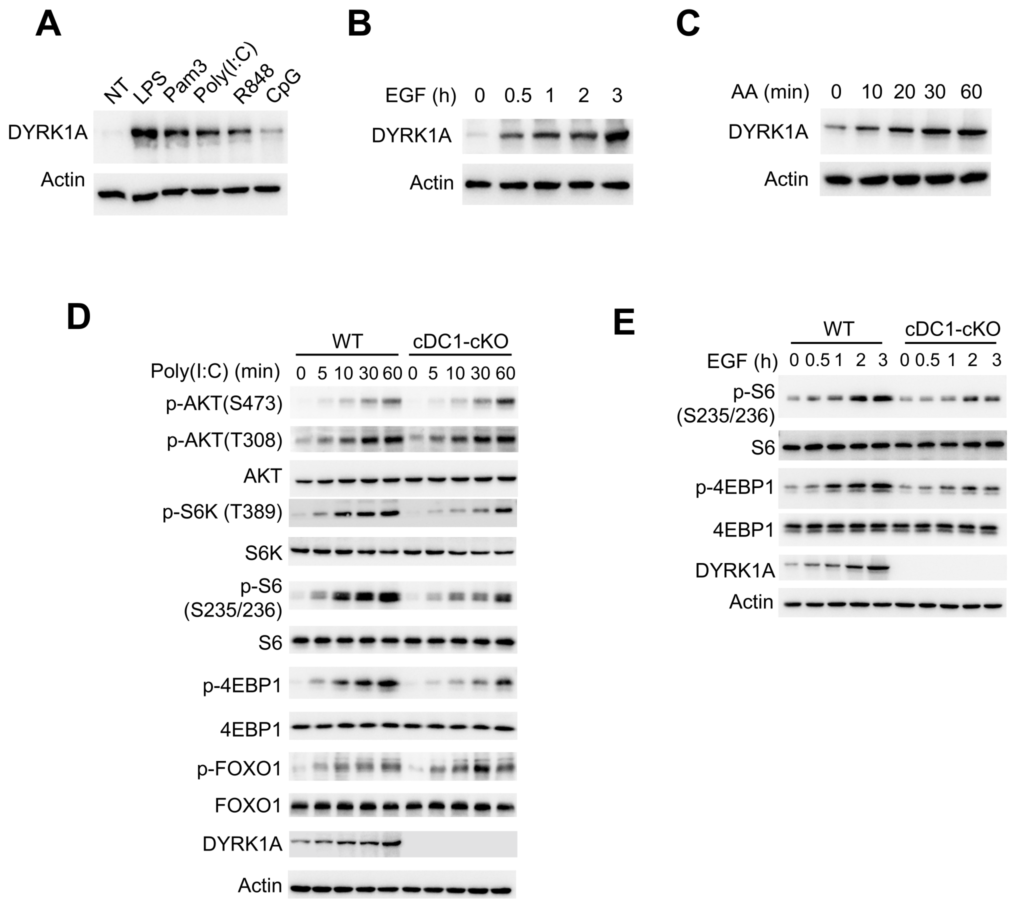

Supplemental Figure 15. DYRK1A deficiency impairs mTORC1 signaling in cDC1s.
(**A**-**C**) Immunoblot analysis of DYRK1A expression in BM-cDC1s stimulated with different TLR agonists (**A**), EGF (**B**), and amino acid (**C**). (**D**-**E**) Immunoblot analysis of the indicated proteins and phosphorylated (p-) proteins in whole-cell lysates of BM-cDC1s stimulated with Poly(I:C) (**D**) and EGF (**E**). Data are representative of three independent experiments.

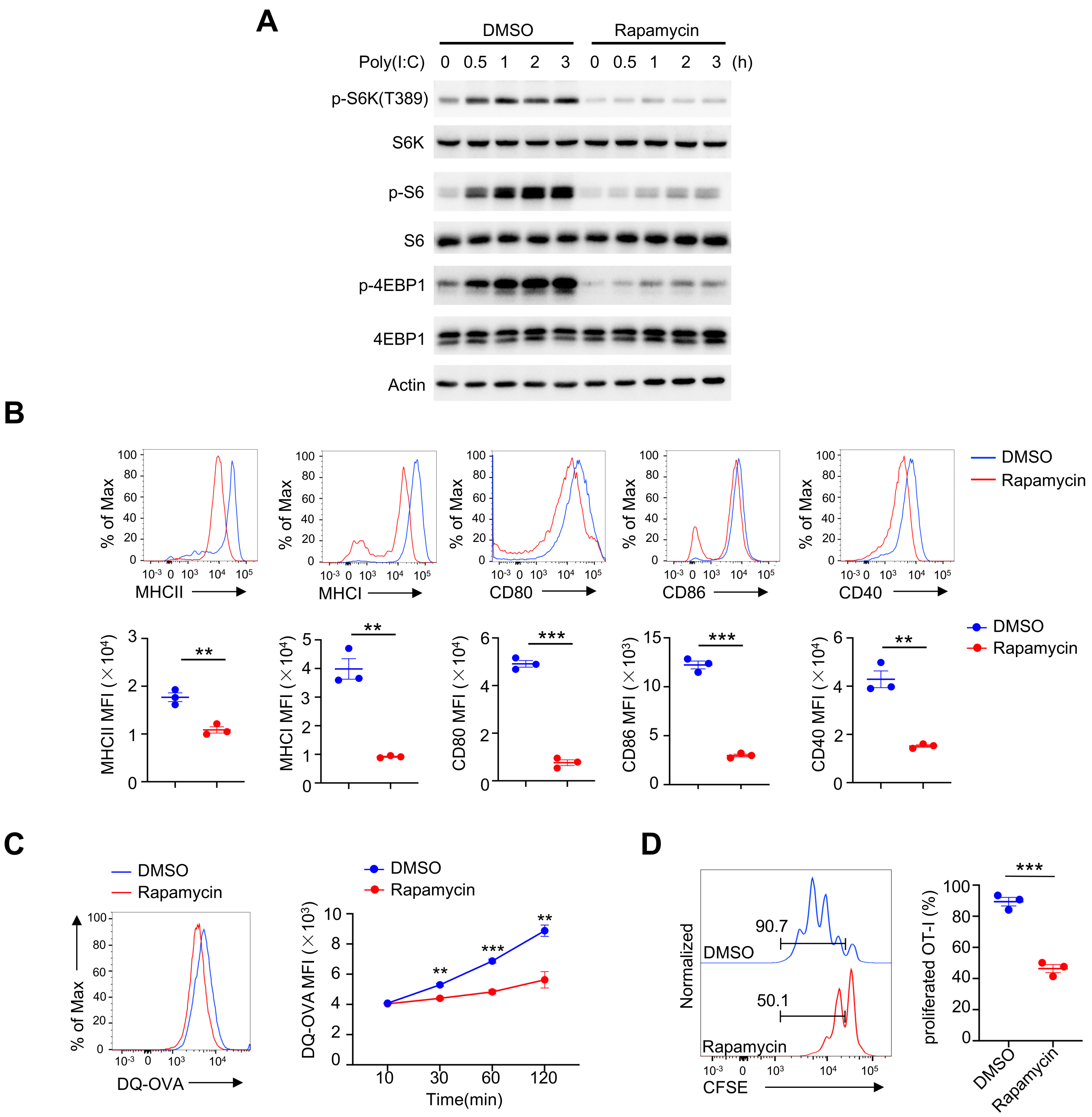

Supplemental Figure 16. Inhibition of the mTORC1 pathway by rapamycin impairs the immune function of cDC1s.
To investigate the role of mTORC1 in regulating cDC1 function, we treated BM-cDC1s with rapamycin (30 μM) for 12 hours. (**A**) Immunoblotting analysis of indicated proteins of whole-cell lysates from BM-cDC1s treated with or without rapamycin, followed by Poly(I:C) stimulation for the indicated time points. (**B**) Flow cytometric analysis of MHCI, MHCII, CD80, CD86, and CD40 levels of BM-cDC1s stimulated with Poly(I:C) for 12 hours in the presence or absence of rapamycin. N=3 per group. (**C**) cDC1s treated with or without rapamycin were incubated with DQ-OVA for the indicated time points, and the mean fluorescence intensity of FITC DQ-OVA was monitored by flow cytometry. N=3 per group. (**D**) Flow cytometric analysis to measure the proliferation of CFSE-labeled OT-I T cells incubated with OVA-pulsed indicated cDC1s. N=3 per group. Data are representative of three independent experiments. Summary data are shown as the mean ± SEM. P values were determined using a two-tailed unpaired Student's *t*-test. **P* < 0.05; ***P* < 0.01; ****P* < 0.001.

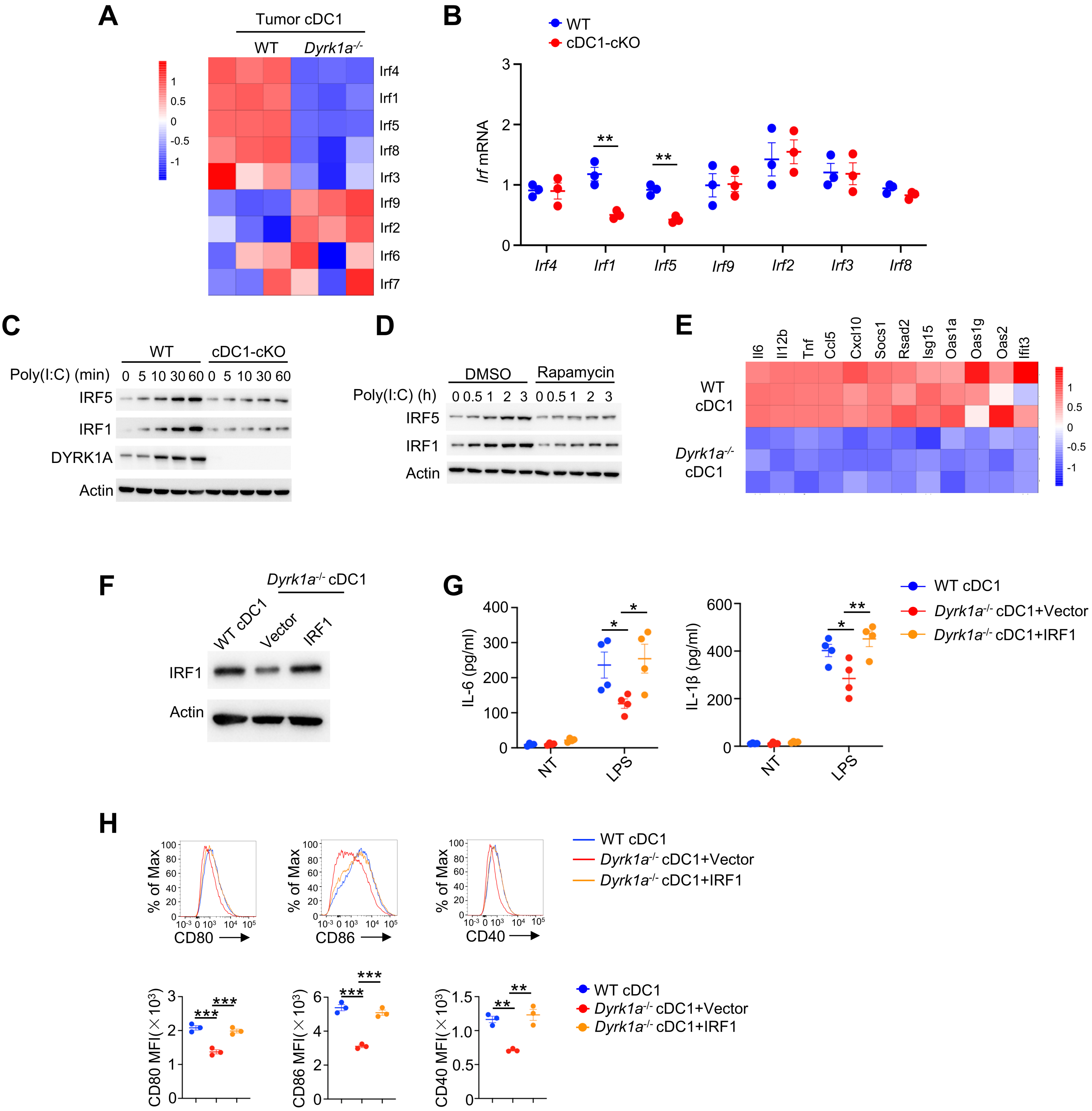

Supplemental Figure 17. DYRK1A promotes cDC1 function through IRF1. (**A**) Heatmap showing *Irf* gene expression levels of wild-type and *Dyrk1a*-deficient tumor-infiltrating cDC1s. (**B**) qPCR analysis of *Irf* genes of wild-type and *Dyrk1a*-deficient tumor-infiltrating cDC1s. (**C**) Western blotting analysis of IRF1 and IRF5 in wild-type and *Dyrk1a*-deficient cDC1s stimulated with Poly (I:C). (**D**) Western blotting analysis of IRF1 and IRF5 in control and Rapamycin-treated cDC1s stimulated with Poly (I:C). (**E**) Heatmap showing gene expression levels of downstream molecules of IRF1 and IRF5 in wild-type and *Dyrk1a*-deficient tumor-infiltrating cDC1s. (**F**) To examine whether DYRK1A modulates cDC1 function via IRF, we performed a rescue experiment by overexpressing IRF1 or vector in *Dyrk1a*-deficient cDC1s. Immunoblotting analysis of IRF1 in the indicated cDC1s. (**G**) ELISA analysis of IL-6 and IL-1β levels of indicated cDC1s, either non-treated (NT) or stimulated with LPS for 24 h. N=4 mice per group. (**H**) Flow cytometric analysis of CD80, CD86, and CD40 levels of indicated cDC1s stimulated with Poly(I:C) for 12 hours. N=3 per group. Data are shown as a representative flow cytometry plot (upper panel) and a summary graph (lower panel). Summary data are shown as the mean ± SEM. P values were determined using one-way ANOVA. **P* < 0.05; ***P* < 0.01; ****P* < 0.001.

A

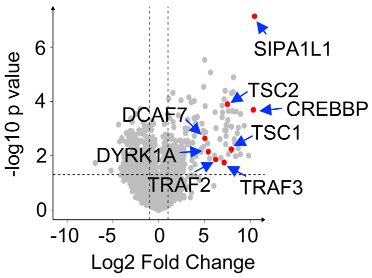

B

gene_name	log2FoldChange	pvalue
CCNA2	7.92257309	0.00085
CREBBP	10.29709339	0.00022
DCAF7	5.308849335	0.00354
EP300	8.894035657	0.00029
LZTS3	8.0935208	4.5E-05
RNF169	8.640407562	0.00535
SIPA1L1	10.48711745	7.3E-08
SIPA1L3	7.212890943	4E-05
TRAF1	7.114150683	8.3E-05
TRAF2	6.656466802	0.00244
TRIP12	6.939690908	0.00025
TSC1	7.860978444	0.00613
TSC2	7.470479965	0.00013
TTC28	8.130933444	1.3E-05
TRAF3	7.113465309	0.01779

C

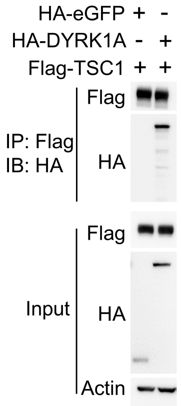

D

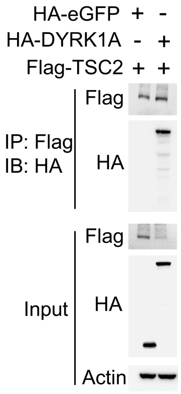

Supplemental Figure 18. Mass spectrometry and Co-IP analyses revealed that DYRK1A interacts with TSC1 and TSC2.
(**A**) Volcano plot showing the mass spectrometric analysis of DYRK1A-interacted proteins, including TRAF3, TSC1, and TSC2. (**B**) List of top fifteen DYRK1A-interacted proteins by mass spectrometric analysis. (**C**) Co-IP analysis of TSC1 interaction with DYRK1A using whole-cell lysates of HEK293 cells transfected with the indicated expression vectors. (**D**) Co-IP analysis of TSC2 interaction with DYRK1A using whole-cell lysates of HEK293 cells transfected with the indicated expression vectors.

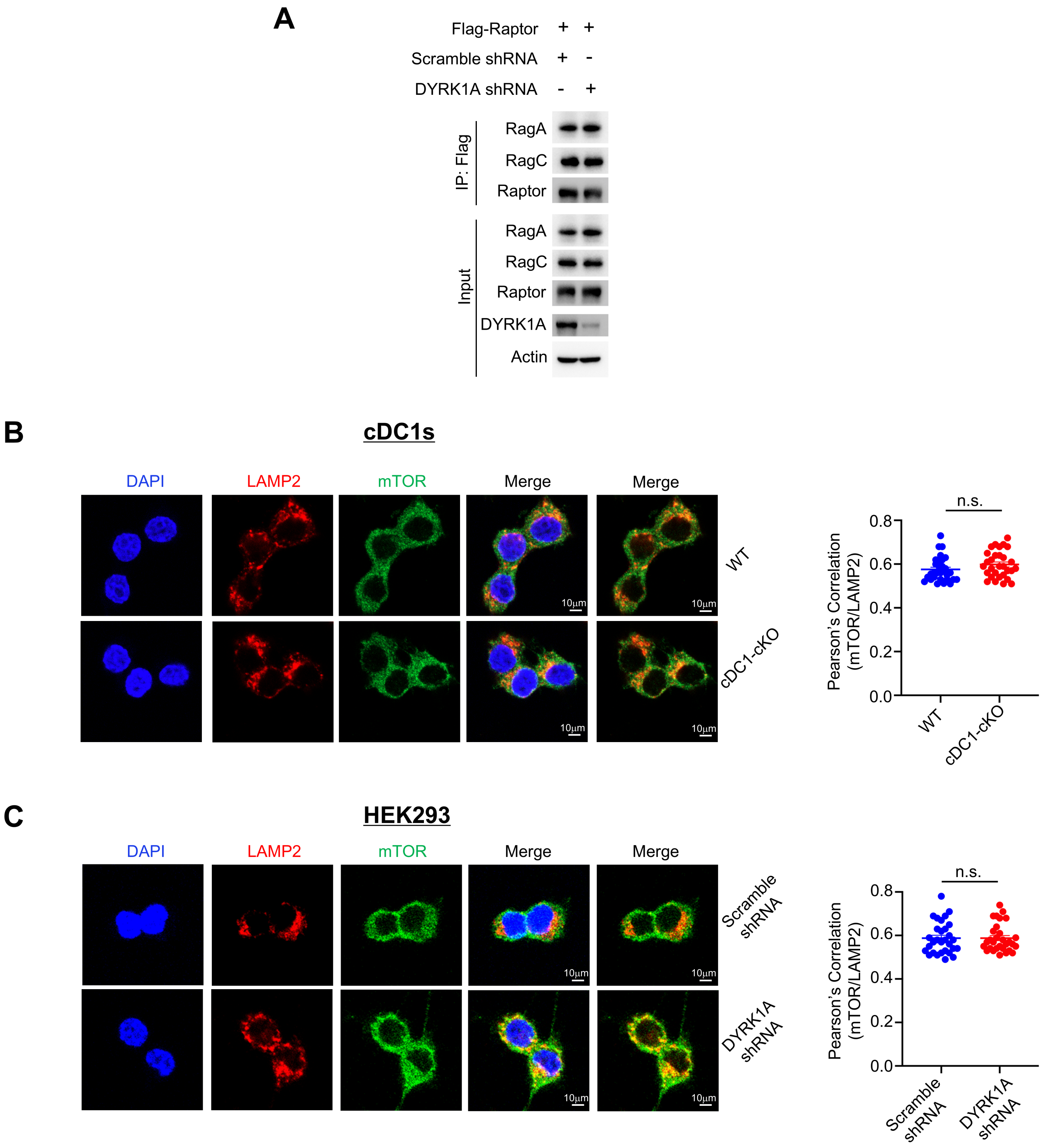

Supplemental Figure 19. DYRK1A regulates mTORC1 activity without influencing Rag GTPase pathway. (**A**) Wild-type and *Dyrk1a*-knockdown HEK293 cells were transfected with Flag-Raptor plasmid. Co-immunoprecipitation of Flag-Raptor and IB analysis of RagA and RagC. (**B, C**) Co-localization of mTOR with the lysosomal marker LAMP2 was analyzed by confocal microscopy in cDC1s of the indicated genotypes (**B**) and in HEK293 cells (**C**). Representative images (left) and quantitative summary graphs (right) of mTOR/LAMP2 co-localization from n=30 individual cells are shown. Scale bars, 10 µm.

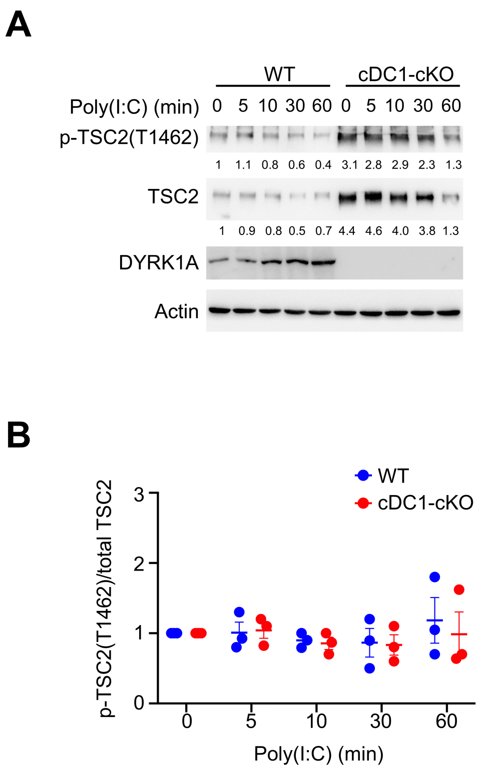

Supplemental Figure 20. DYRK1A deficiency did not affect TSC2 phosphorylation at T1462 in cDC1s.
(**A**) Immunoblot analysis of p-TSC2(T1462) and TSC2 in WT and *Dyrk1a*-deficient BM-cDC1s stimulated with Poly(I:C) at different time points. (**B**) A summary graph of the ratio between p-TSC2(T1462) versus total TSC2. N=3 per group.

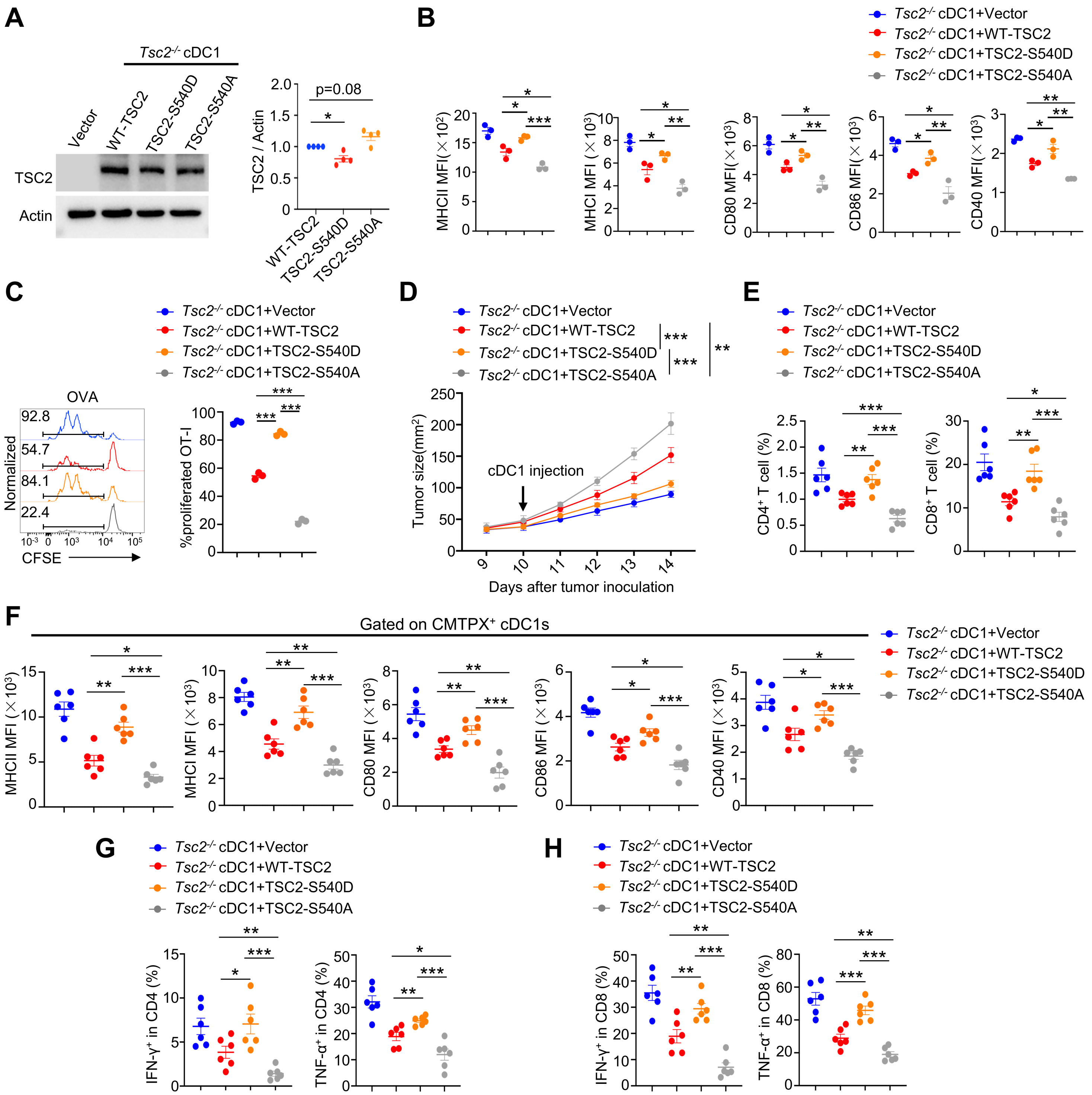

Supplemental Figure 21. S540 phosphorylation of TSC2 is essential for cDC1-mediated antitumor immunity. We reconstituted *Tsc2*-/- cDC1s with wild-type, phosphomimic (S540D), or phosphorylation-deficient (S540A) TSC2 plasmids. (**A**) Immunoblotting analysis of *Tsc2*-/- cDC1s transfected with the indicated plasmids at steady state (Left panel). Quantification of TSC2 protein levels of indicated cDC1s (Right panel). (**B**) Flow cytometric analysis of MHCI, MHCII, CD80, CD86, and CD40 levels of indicated cDC1s stimulated with Poly(I:C) for 12 hours. N=3 per group. (**C**) Flow cytometric analysis to measure the proliferation of CFSE-labeled OT-I T cells incubated with OVA-pulsed cDC1s. N=3 per group. (**D**) Tumor growth curves of wild-type mice *s.c.* injected with B16-F10 melanoma cells and then treated *i.t.* with indicated BM-cDC1s. The *Tsc2*-/- cDC1s were reconstituted with the indicated plasmids, pulsed with tumor cell lysates, and matured with Poly(I:C). N=6 mice per group. (**E**) Flow cytometric analysis of the frequencies of tumor-infiltrating CD4+ and CD8+ T cells. (**F**) Flow cytometric analysis of the surface expression levels of MHCI, MHCII, CD80, CD86, and CD40 on tumor-infiltrating CMTPX-labeled cDC1s. (**G-H**) Flow cytometric analysis of the frequencies of IFN-γ-, TNF-α-producing CD4+ T cells (**H**) and CD8+ T cells (**I**) in the tumors of tumor-bearing mice *i.t.* injected with indicated cDC1s. Data are representative of three independent experiments. Summary data are shown as the mean ± SEM. P values were determined using one-way ANOVA (**A-C, E-H**), or two-way ANOVA with Bonferroni correction (**D**). *$P < 0.05$; **$P < 0.01$; ***$P < 0.001$.

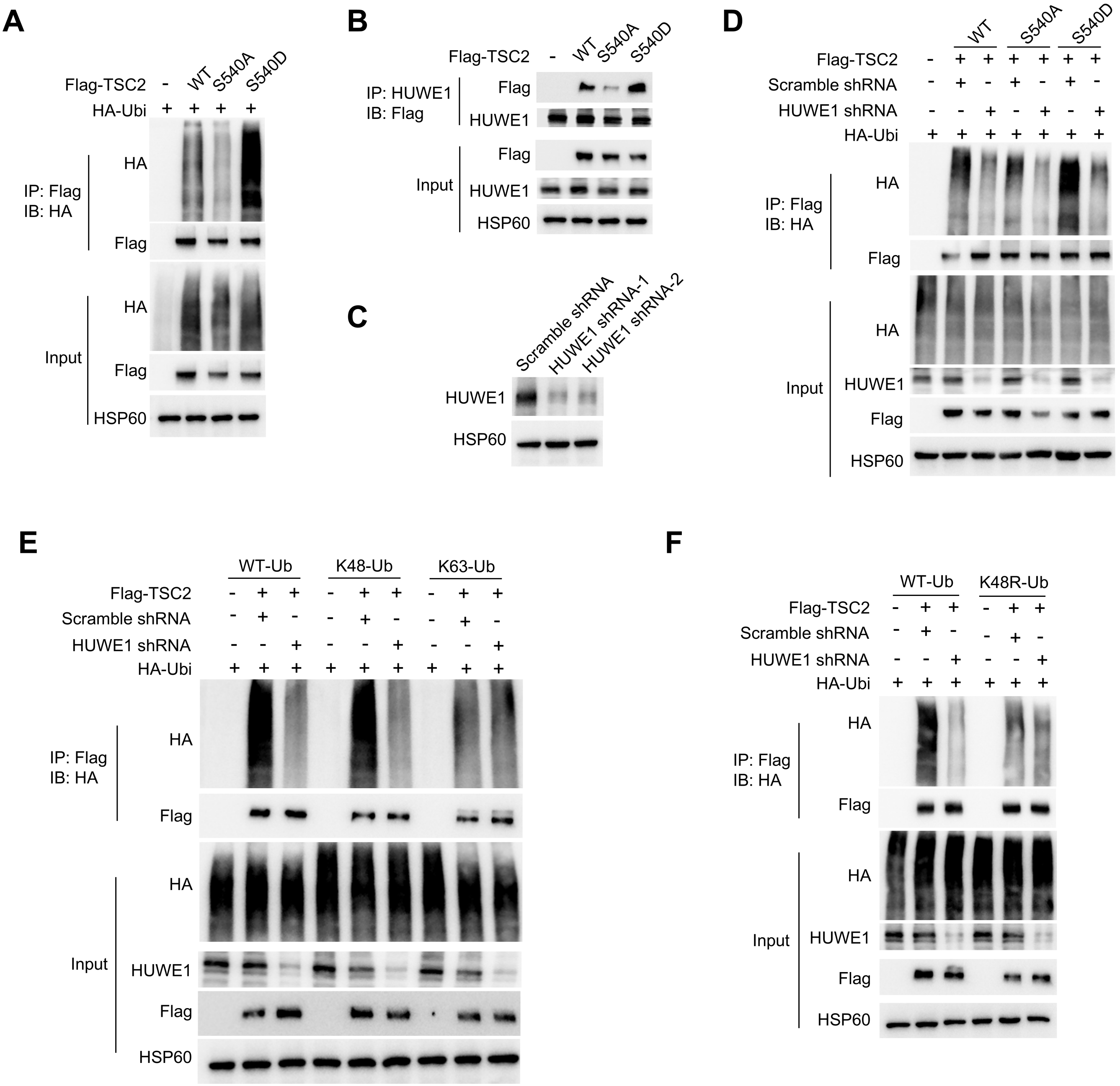

Supplemental Figure 22. The E3 ubiquitin ligase HUWE1 mediates TSC2 ubiquitination in a phosphorylation-dependent manner.
(**A**) Ubiquitination of WT TSC2 and mutant TSC2 (S540A, S540D) in HEK293 cells transfected with the indicated expression vectors, assessed by immunoblot analysis with anti-HA (HA-ubiquitin) after immunoprecipitation with anti-Flag or by immunoblot analysis with input proteins in lysates without immunoprecipitation. (**B**) Co-IP analysis of HUWE1 interaction with TSC2 using whole-cell lysates of HEK293 cells transfected with the indicated expression vectors. (**C**) Immunoblot analysis of HUWE1 in HEK293 cells transfected with the indicated shRNAs. (**D**) Ubiquitination of WT TSC2 and mutant TSC2 (S540A, S540D) in HEK293 cells transfected with the indicated shRNAs or expression vectors, assessed by immunoblot analysis with anti-HA after immunoprecipitation with anti-Flag or by immunoblot analysis with input proteins in lysates without immunoprecipitation. (**E**-**F**) Ubiquitination of WT TSC2 in HEK293 cells transfected with the indicated shRNAs or expression vectors, assessed by immunoblot analysis with anti-HA (HA-ubiquitin) after immunoprecipitation with anti-Flag or by immunoblot analysis with input proteins in lysates without immunoprecipitation.

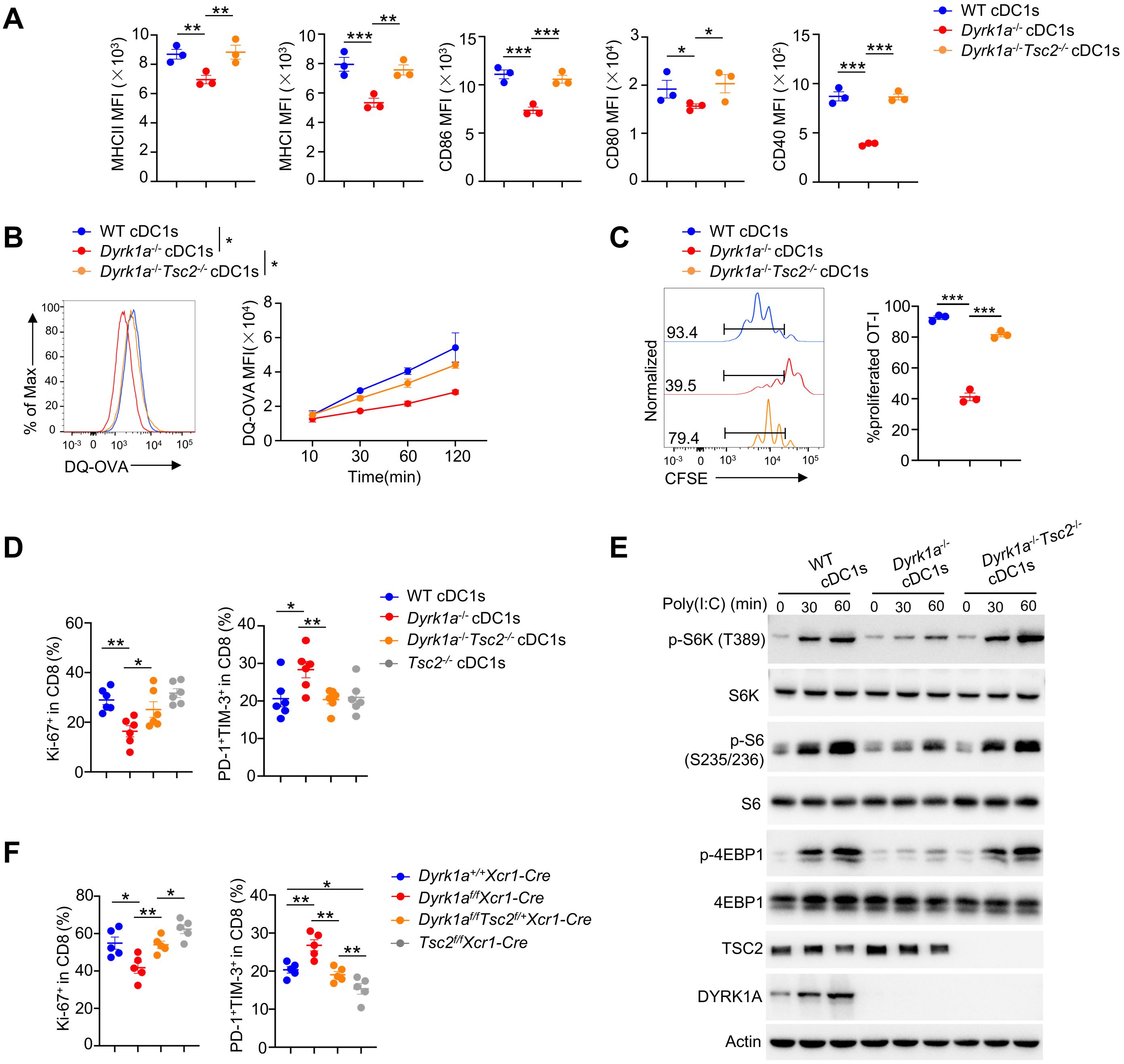

Supplemental Figure 23. Deletion of TSC2 in *Dyrk1a*-deficient cDC1s restores its antitumor function.
(**A**) Flow cytometric analysis of MHCI, MHCII, CD80, CD86, and CD40 levels of indicated cDC1s stimulated with Poly(I:C) for 12 hours. N=3 per group. (**B**) To assess antigen-processing ability, cDC1s were incubated with DQ-OVA for the indicated times, and the mean fluorescence intensity of FITC-DQ-OVA in the indicated cDC1s was monitored by flow cytometry. N=3 per group. (**C**) Flow cytometric analysis to measure the proliferation of CFSE-labeled OT-I T cells incubated with OVA-pulsed cDC1s. N=3 per group. (**D**) Flow cytometric analysis of Ki-67 (left panel) and TIM-3, PD-1 (right panel) levels of tumor-infiltrating $CD8^+$ T cells from mice *i.t.* injected with indicated cDC1s. N=6 mice per group. (**E**) Immunoblot analysis of indicated proteins in whole-cell lysates of WT, *Dyrk1a*$^{-/-}$, *Dyrk1a*$^{-/-}$*Tsc2*$^{-/-}$ BM-cDC1s stimulated with Poly(I:C) at indicated time points. (**F**) Flow cytometric analysis of Ki-67 (left panel) and TIM-3, PD-1 (right panel) levels of tumor-infiltrating $CD8^+$ T cells from *Dyrk1a*$^{+/+}$*Xcr1*-Cre, *Dyrk1a*$^{fl/fl}$*Xcr1*-Cre, *Dyrk1a*$^{fl/fl}$*Tsc2*$^{fl/+}$*Xcr1*-Cre, and *Tsc2*$^{fl/fl}$*Xcr1*-Cre mice. N=5 mice per group. Data are representative of three independent experiments. Summary data are shown as the mean ± SEM. P values were determined using one-way ANOVA. *$P < 0.05$; **$P < 0.01$; ***$P < 0.001$.

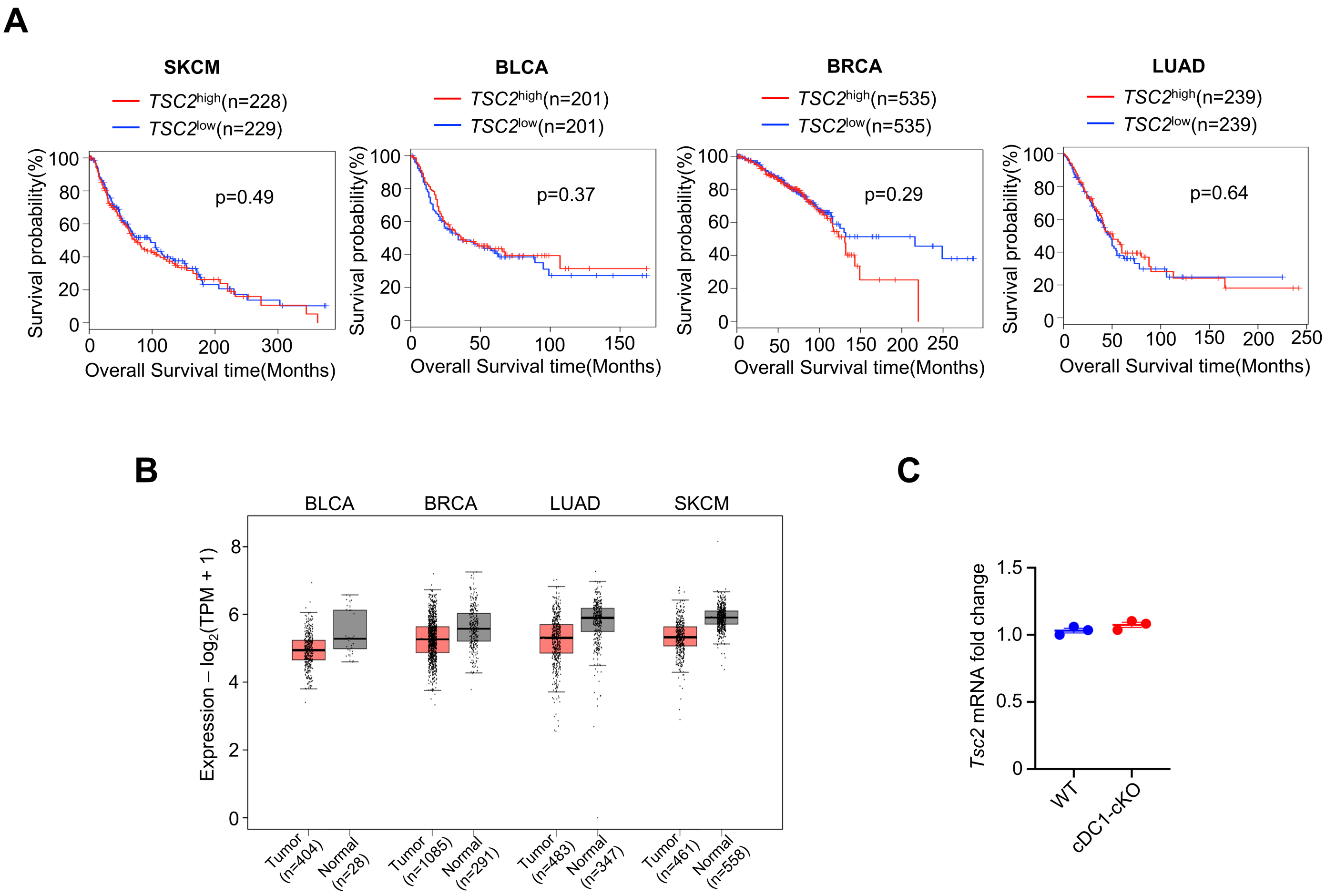

Supplemental Figure 24. *TSC2* gene expression is dispensable for overall survival across multiple human cancer types.
(**A**) Overall survival of TCGA SKCM, BLCA, BRCA, and LUAD cohorts based on the expression levels of the *Tsc2* gene. P values (Wald Chi-square test) were determined based on a univariate Cox proportional-hazards model (*TSC2*^{high} versus *TSC2*^{low}). (**B**) *TSC2* gene expression in tumor and normal tissue from SKCM, BLCA, BRCA, and LUAD cohorts. (**C**) Real-time quantitative reverse-transcription PCR analysis of *Tsc2* mRNA level in the tumor-infiltrating cDC1 isolated from wild-type and *Dyrk1a*-cDC1-cKO mice. N=3 per group.

SUPPLEMENTAL TABLES

1. Supplemental Table 1. List of primers used for PCR of mouse genes.
2. Supplemental Table 2. List of primers used for qRT-PCR of mouse genes.
3. Supplemental Table 3. List of primers used for shRNA.
4. Supplemental Table 4. List of genes used for the correlation analysis.

Supplemental Table 1. List of primers used for PCR of mouse genes.

Primer	Application	Sequence
Dyrk1a-flox-F	PCR	5’- TACCTGGAGAAGAGGGCAAG-3’
Dyrk1a-flox-R	PCR	5’- GGCATAACTTGCATACAGTGG-3’
Tsc2-flox-F	PCR	5’- GGCTGGCTTATAGGTTCAGCA-3’
Tsc2-flox-R	PCR	5’- CCCTGTGGCAGTTGTAATATGC -3’
Cd11c cre-F	PCR	5’- ACTTGGCAGCTGTCTCCAAG-3’
Cd11c cre-R	PCR	5’- GCGAACATCTTCAGGTTCTG-3’
OT-I-Transgenic-F	PCR	5’- CAGCAGCAGGTGAGACAAAGT-3’
OT-I- Transgenic-R	PCR	5’- GGCTTTATAATTAGCTTGGTCC-3’
OT-I-Internal Control-F	PCR	5’- CAAATGTTGCTTGTCTGGTG-3’
OT-I- Internal Control-R	PCR	5’- GTCAGTCGAGTGCACAGTTT-3’
OT-II-Transgenic-F	PCR	5’- GCTGCTGCACAGACCTACT-3’
OT-II-Transgenic-R	PCR	5’- CAGCTCACCTAACACGAGGA-3’
OT-II-Internal Control-F	PCR	5’-CTAGGCCACAGAATTGAAAGATCT-3’
OT-II-Internal Control-R	PCR	5’-GTAGGTGGAAATTCTAGCATCATCC-3’
Xcr1 cre-Common	PCR	5’- TCAAGTTCCGCAGACACCTA -3’
Xcr1 cre-Wild type-R	PCR	5’- GTGCACGAAGTGTTGCTTTG -3’
Xcr1 cre-Mutant-R	PCR	5’- ACCGTCGACCCCATAGTCAT -3’

Supplemental Table 2. List of primers used for qRT-PCR of mouse genes.

Primer	Application	Sequence
mDyrk1a-F	qRT-PCR	5’-GGGGACGATTCCAGTCATAAGA-3
mDyrk1a-R	qRT-PCR	5’-GGAGTCGATTTCATACCGATCC-3’
mActin-F	qRT-PCR	5’-GGAGTTCGAGGAACCCTAGTG-3’
mActin-R	qRT-PCR	5’-CCAGTTGGTAACAATGCCATGT-3’

Supplemental Table 3. List of primers used for shRNA.

Primer	Sequence
hHUWE1 sh1-F	5’-CCGGTTTGATGTCAAGCGCAAATATCTCGAG ATATTTGCGCTTGACATCAAATTTTTG-3’
hHUWE1 sh1-R	5’-AATTCAAAAATTTGATGTCAAGCGCAAATATCTCGAG ATATTTGCGCTTGACATCAAA-3’
hHUWE1 sh2-F	5’-CCGGCCTAGGCTGCAGGACTAATATCTCGAG ATATTAGTCCTGCAGCCTAGGTTTTTG-3’
hHUWE1 sh2-R	5’-AATTCAAAAACCTAGGCTGCAGGACTAATATCTCGAG ATATTAGTCCTGCAGCCTAGG-3’

Supplemental Table 4. List of genes used for the correlation analysis.

Activated_CD8	cytotoxic_CD4	DYRK1A_cDC1	mTOR
CD69	CD4	CD80	AKT2
CCR7	FGFBP2	CD86	BRAF
CD27	CX3CR1	CCR7	EIF4B
BTLA	GNLY	HLA-DRA	IGF1
CD40LG	GZMB	HLA-DRB1	MAP2K1
IL2RA	GZMA	HLA-DQB1	MAPK1
CD3E	CCL5	HLA-DQA1	MAPK3
CD47	NKG7	CD40	MTOR
EOMES	GZMH	IL6	PIK3CB
GNLY	S1PR5	IL12A	PIK3R1
GZMA	FGR	IL18	PIK3R2
GZMB	ADGRG1	ICAM1	PIK3R3
PRF1	CST7	ICAM2	RPS6KA
IFNG	C1orf21	ICAM3	NPRL2
CD8A	PRSS23	CCR7	TNF
CD8B	PLEK	ICOSL	TSC1
CD95L	ZEB2	TNF	TSC2
LAMP1	TARP	DYRK1A	TBC1D7
LAG3	KLRG1	ITGAX	ATP6V1F
CTLA4	FCRL6	XCR1	ATP6V1B2
HLA-DRA	SLAMF7	CLEC9A	ATP6V1E1
TNFRSF4	ADRB2		ATP6V1D
ICOS	TGFBR3		ATP6V1C1
TNFRSF9	PRF1		
TNFRSF18	CCL4		
