# Supplementary material for: DYRK1A enhances antitumor immunity in type 1 conventional dendritic cells via mTORC1 activation
Source: J Clin Invest. 2026 Apr 23;136(12):e199108. doi: 10.1172/JCI199108 (PMC13262734; doi:10.1172/JCI199108)
Supplement: Unedited blot and gel images [file jci-136-199108-s262.pdf]

Figure 1B

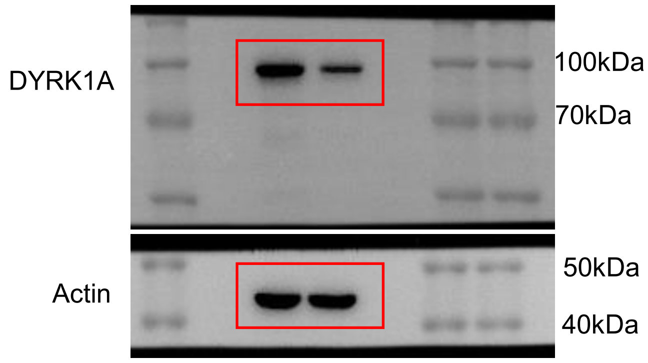

Figure 1C

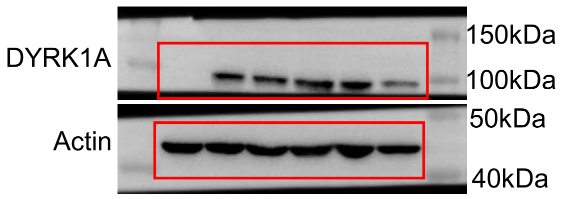

Figure 1D

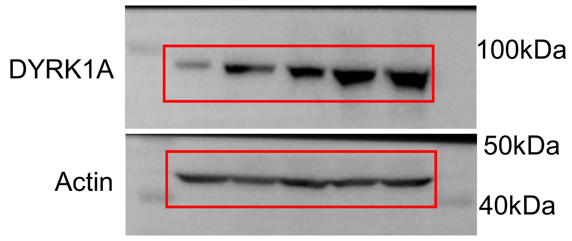

Figure 1E

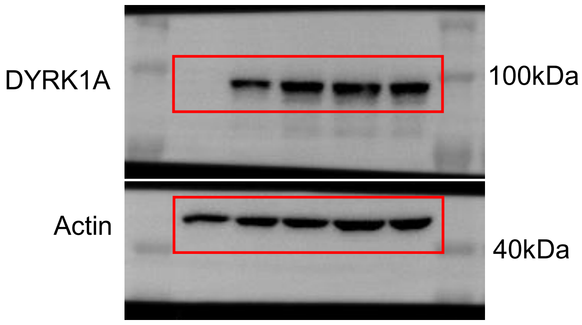

Figure 1F

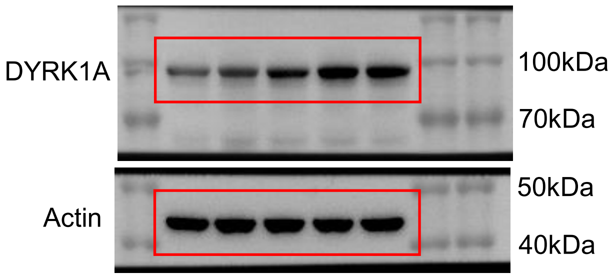

Figure 1G

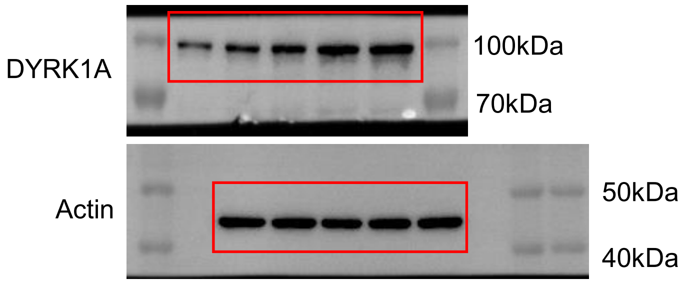

Figure 1H

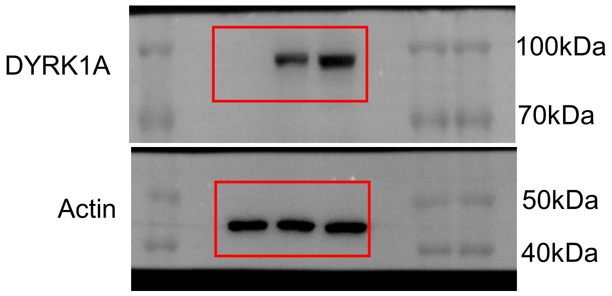

Full scans of blots for figures 1B-1H

Figure 3B

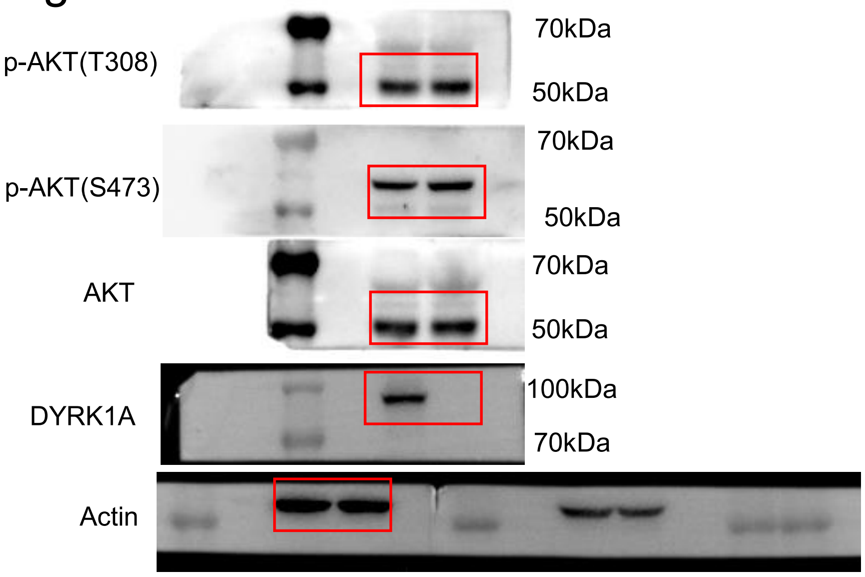

Figure 3F

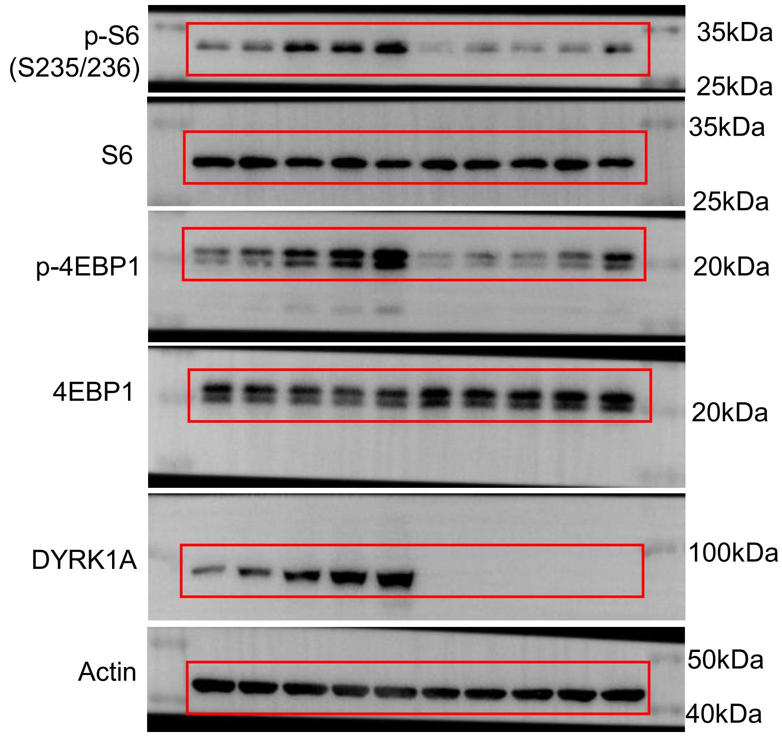

Figure 3G

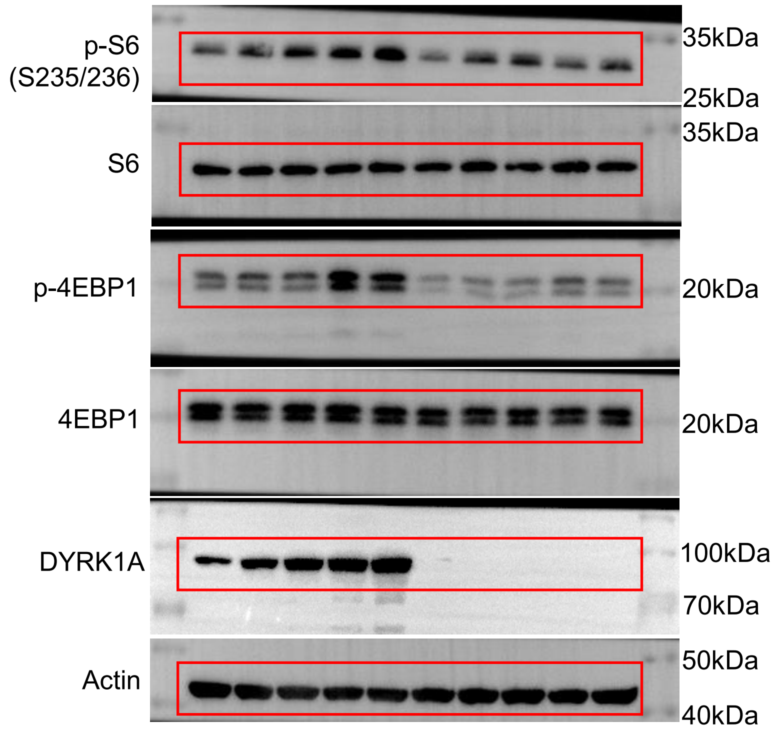

Figure 3E

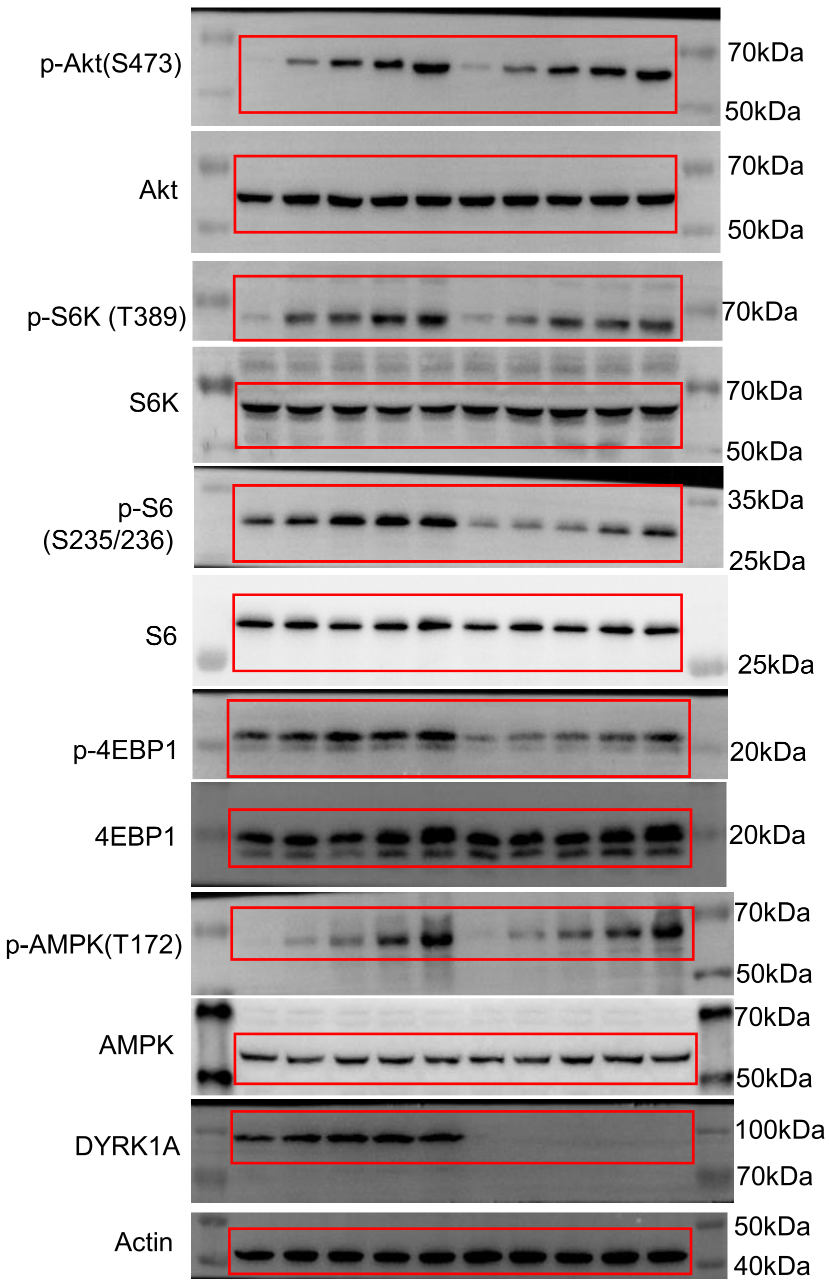

Full scans of blots for figures 3B and 3E-3G

Figure 6A

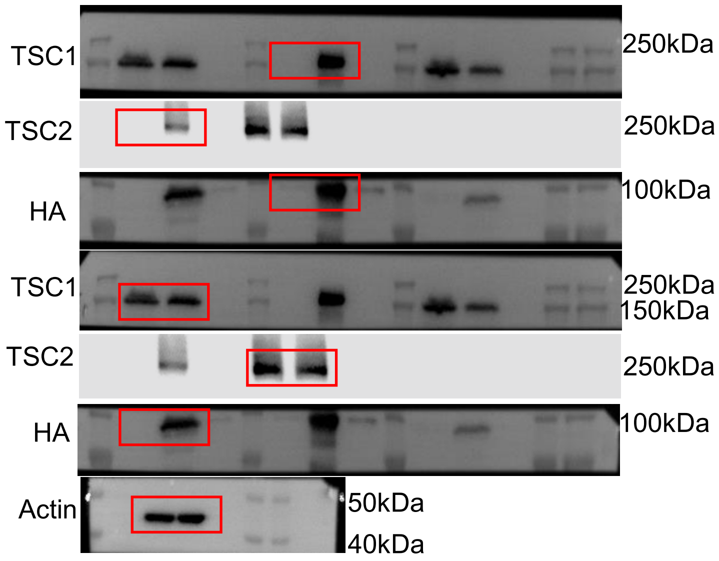

Figure 6B

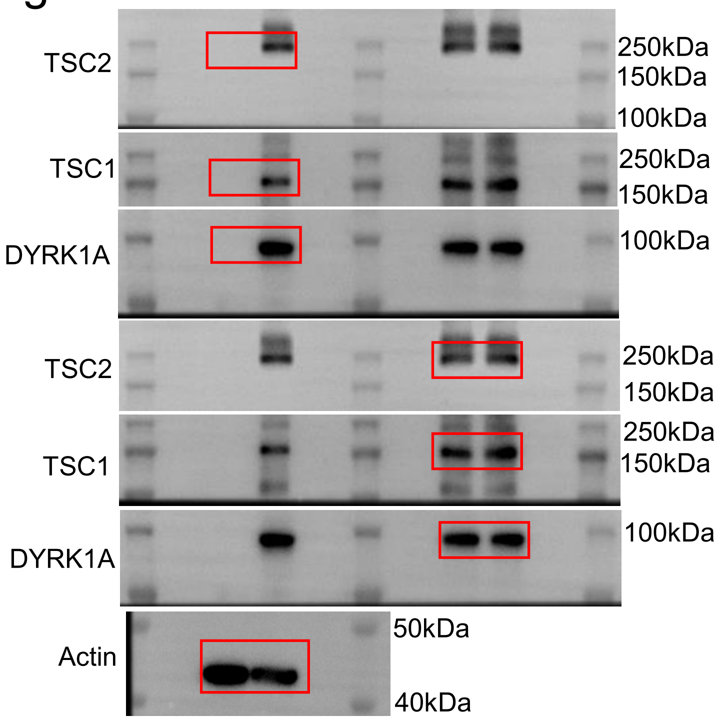

Figure 6C

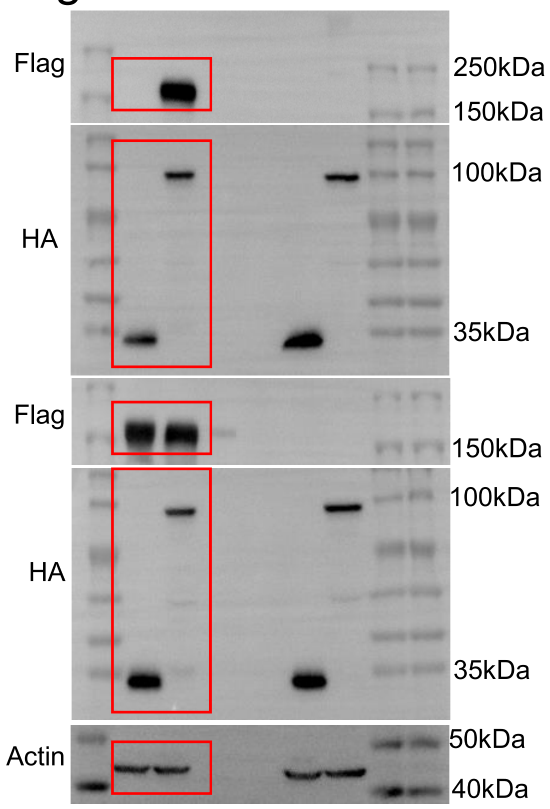

Figure 6D

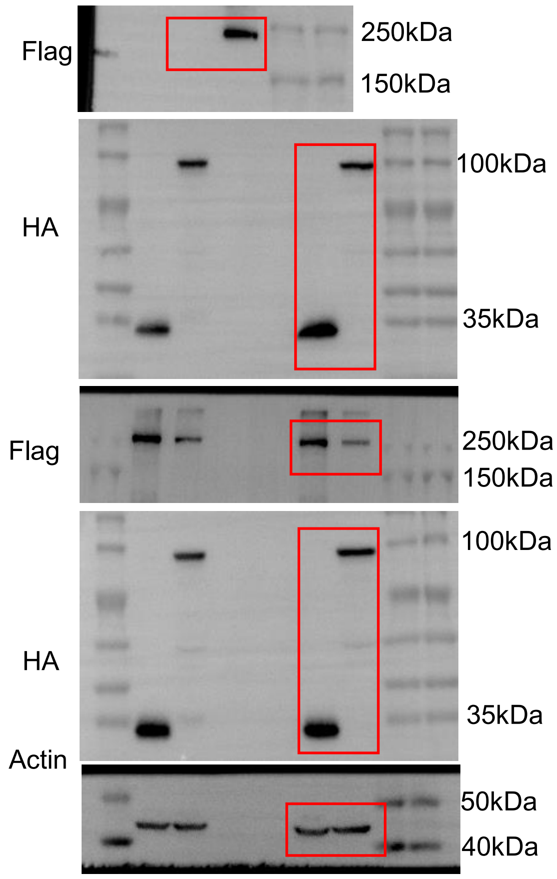

Figure 6E

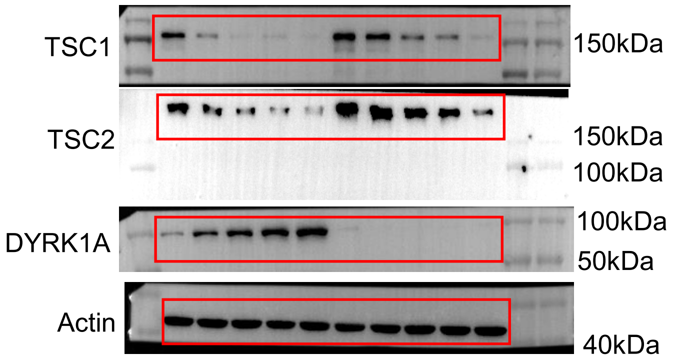

Full scans of blots for figures 6A-6E

Figure 6F

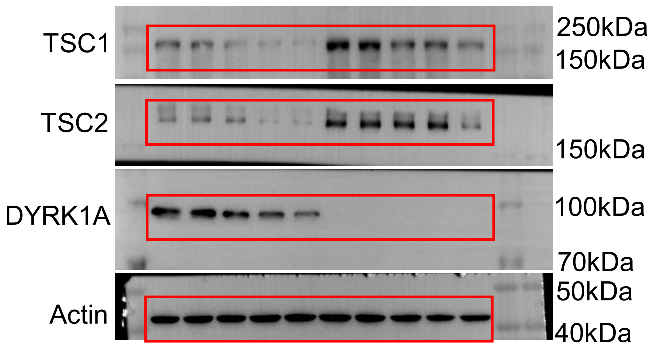

Figure 6G

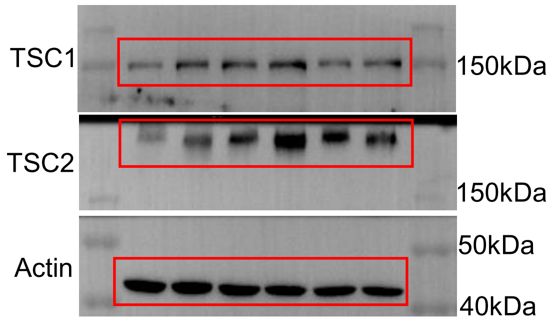

Figure 6H

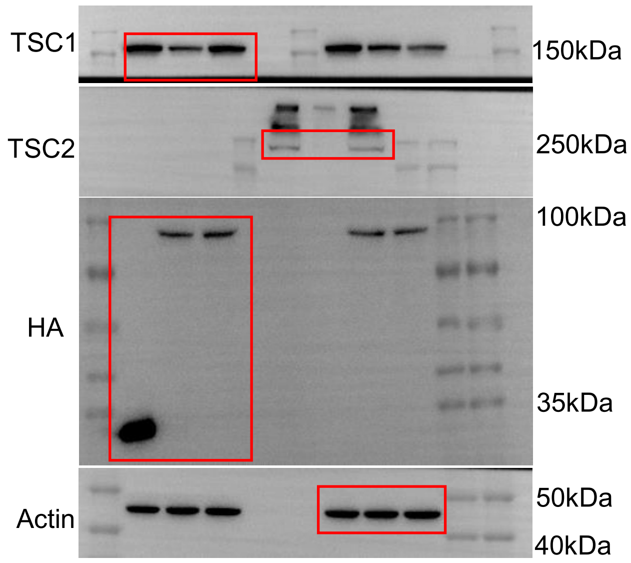

Figure 6I

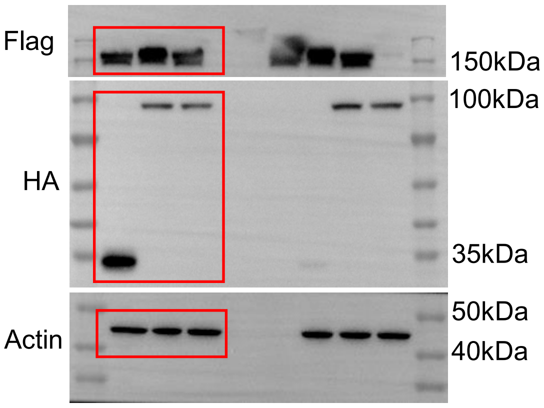

Figure 6J

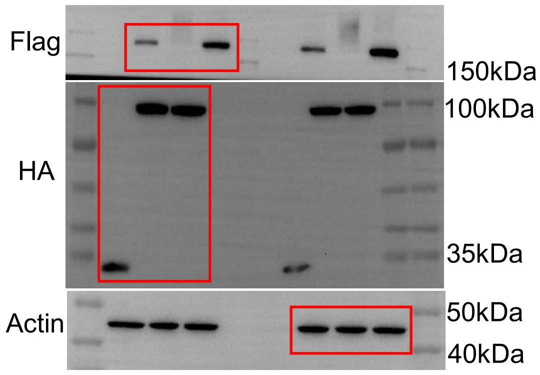

Figure 6L

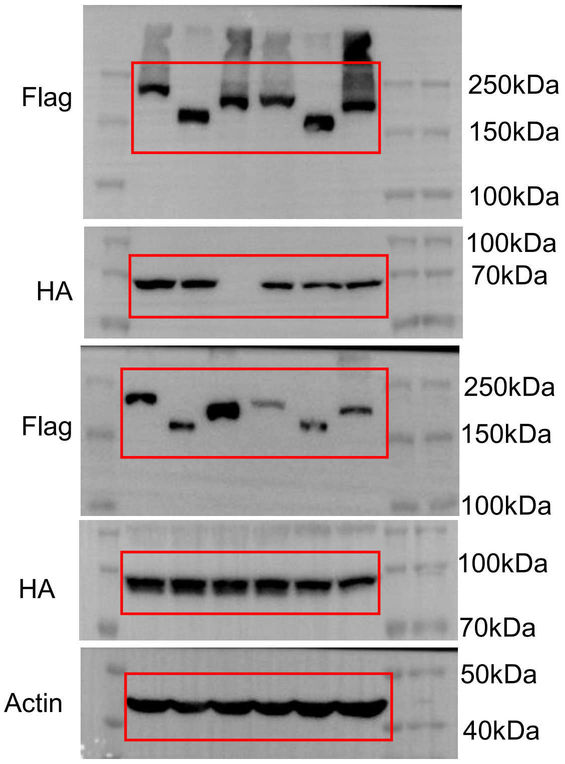

Figure 6N

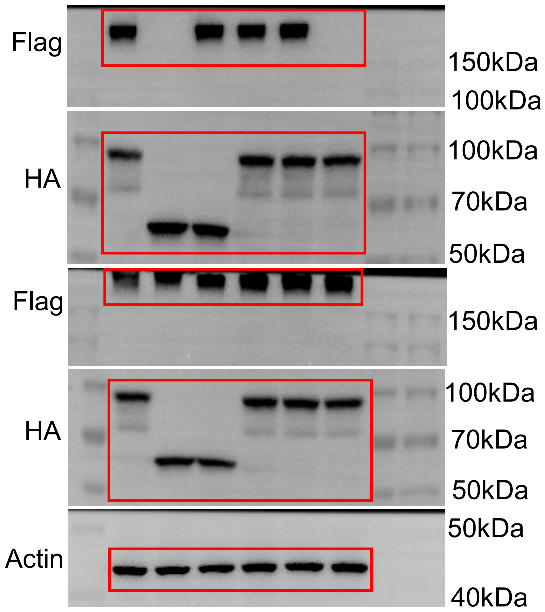

Full scans of blots for figures 6F-6J、6L and 6N

Figure 7A

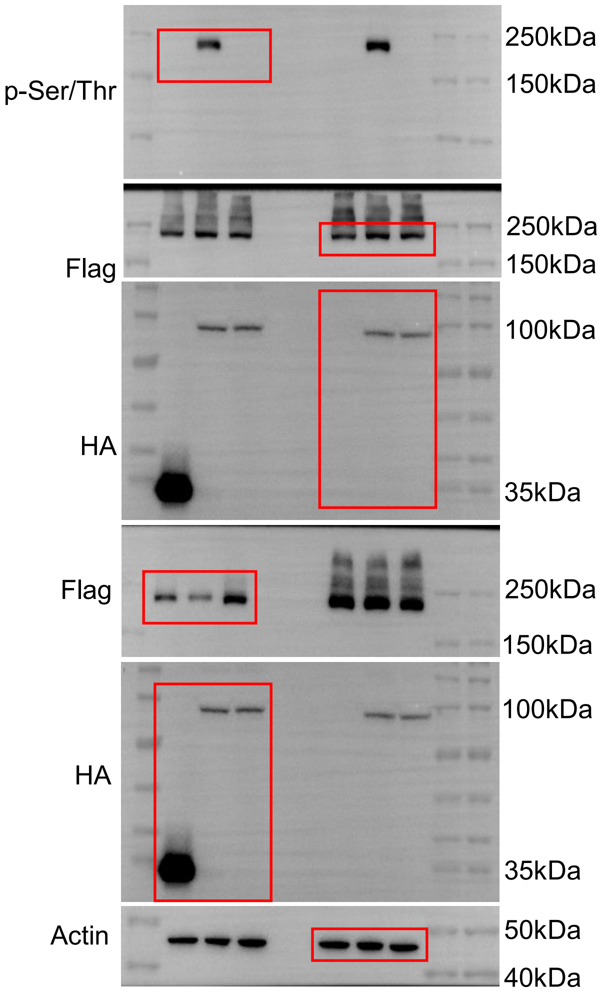

Figure 7F

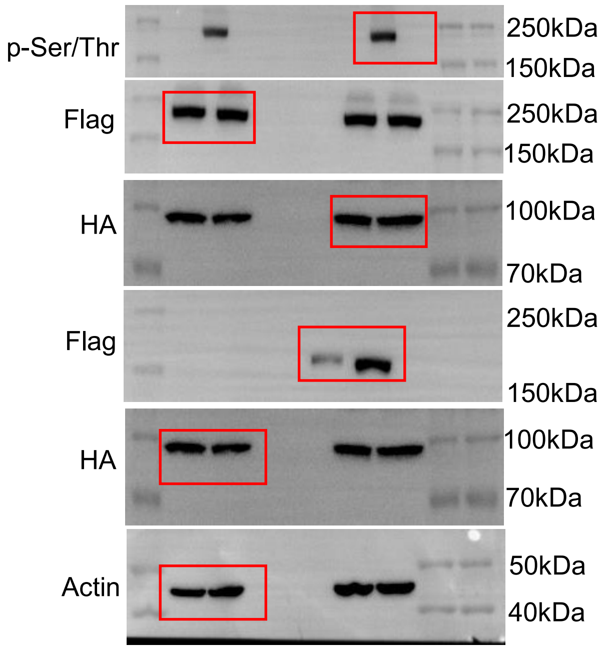

Figure 7G

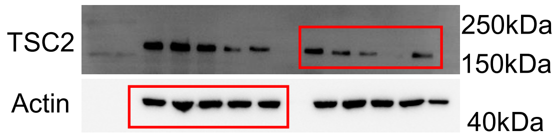

Figure 7H

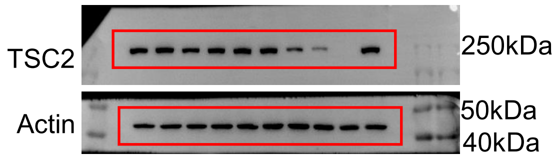

Figure 7J

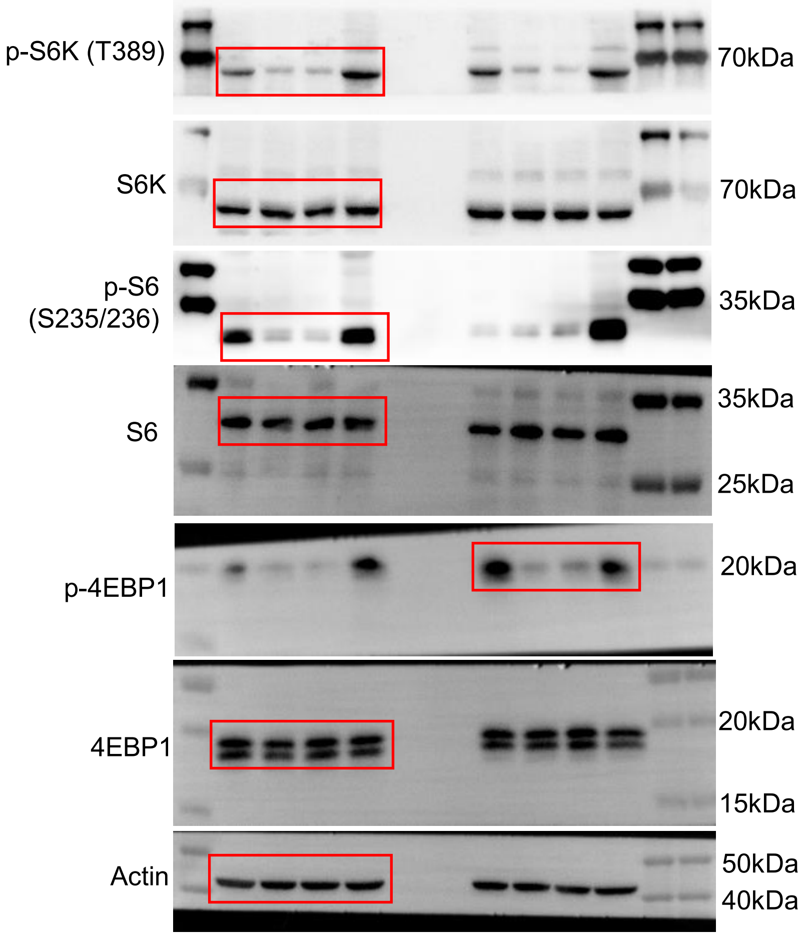

Full scans of blots for figures 7A、7F-7H and 7J

sFig 1F

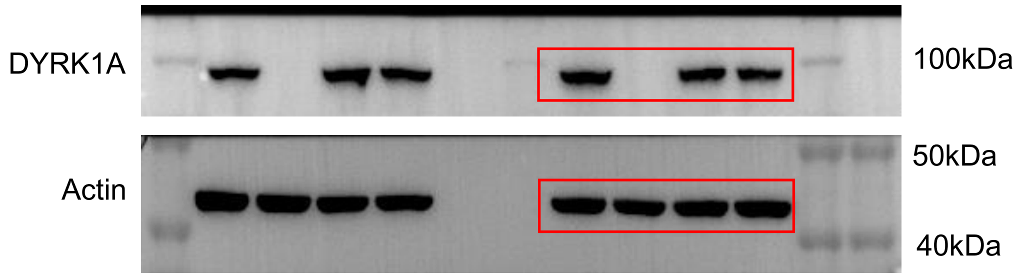

sFig 1I

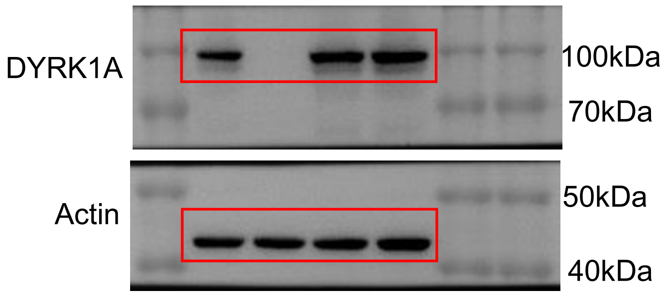

Full scans of blots for supplemental figures 1F and 1I

sFig 7F

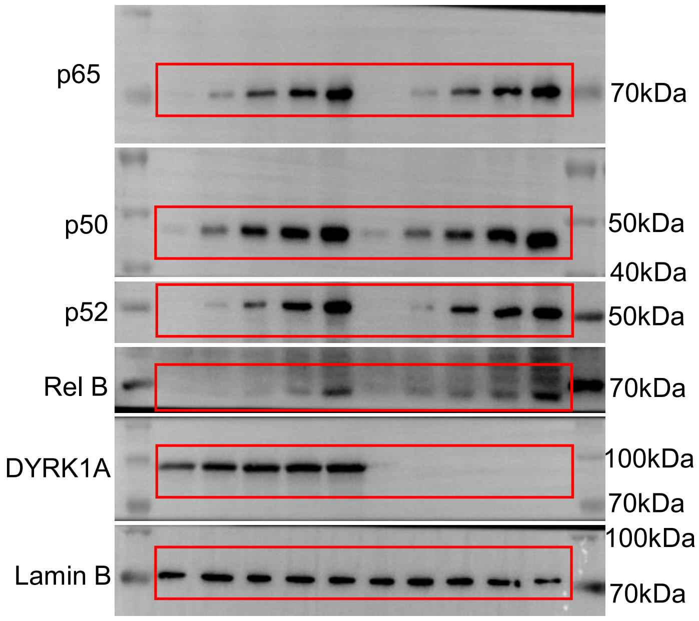

Full scans of blots for supplemental figure 7F

sFig 15A

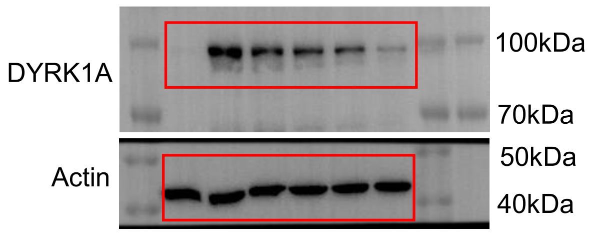

sFig 15B

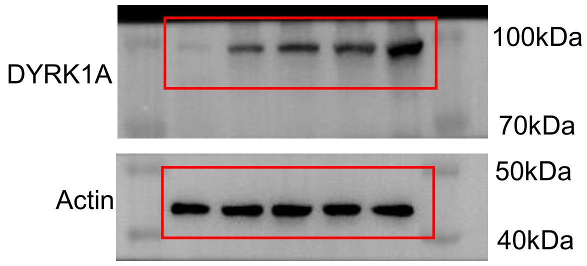

sFig 15C

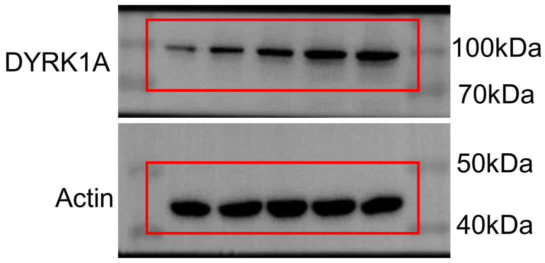

sFig 15D

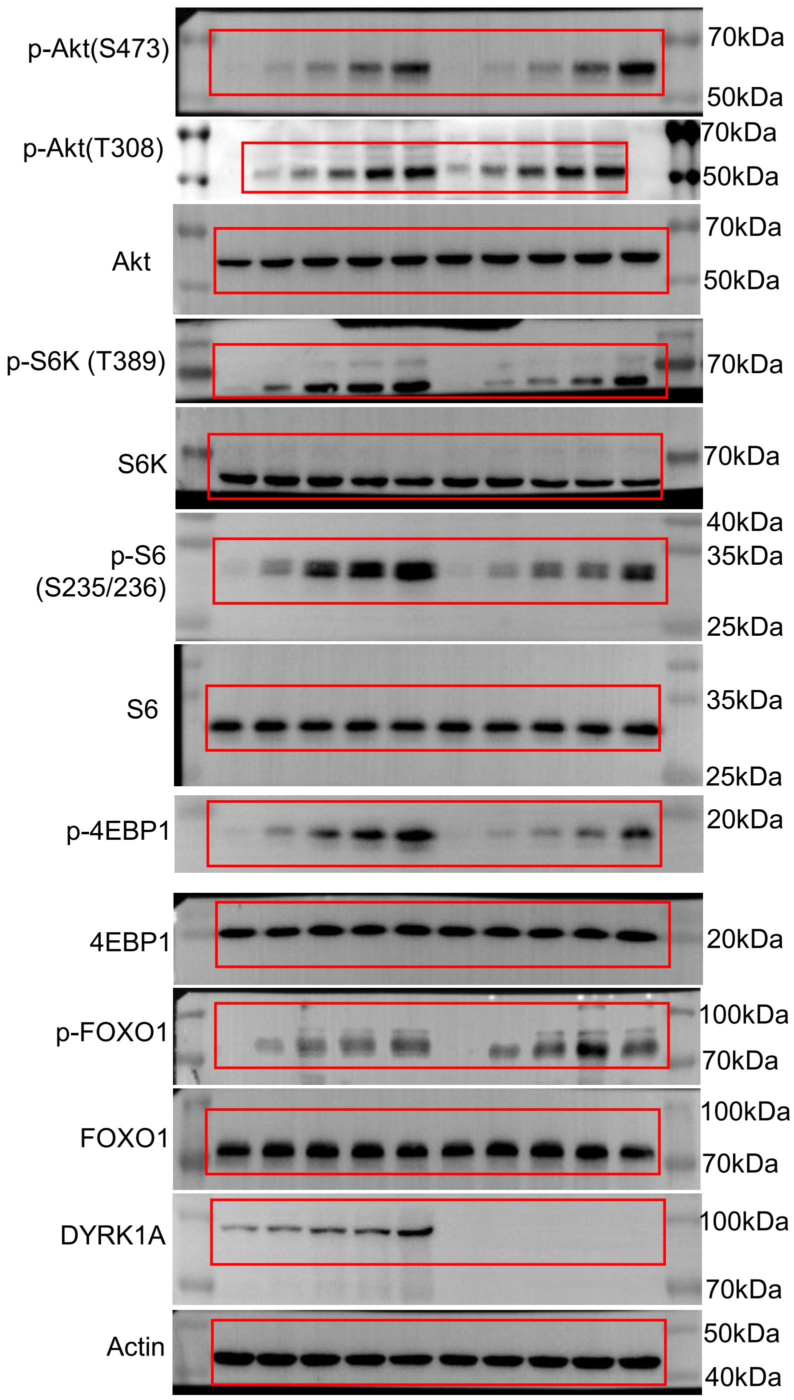

sFig 15E

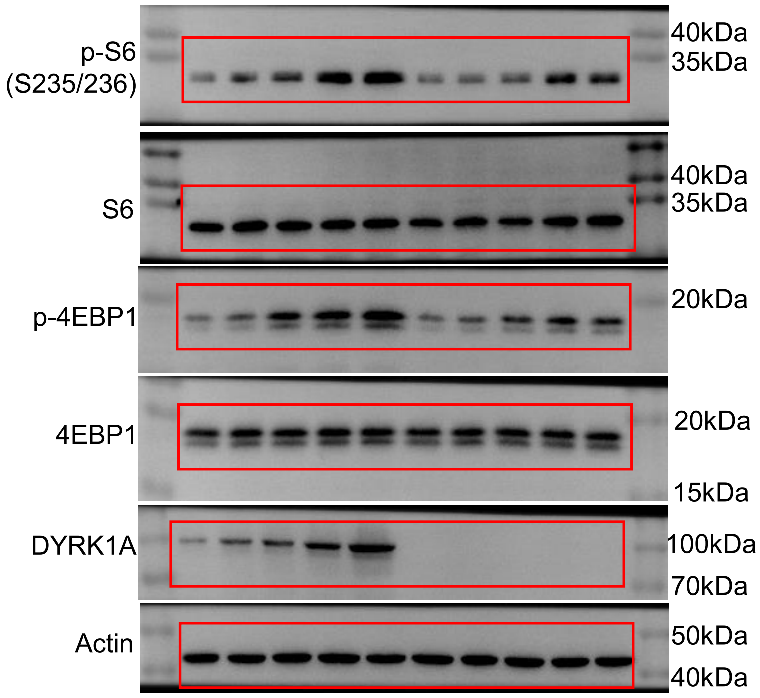

Full scans of blots for supplemental figures 15A-15E

sFig 16A

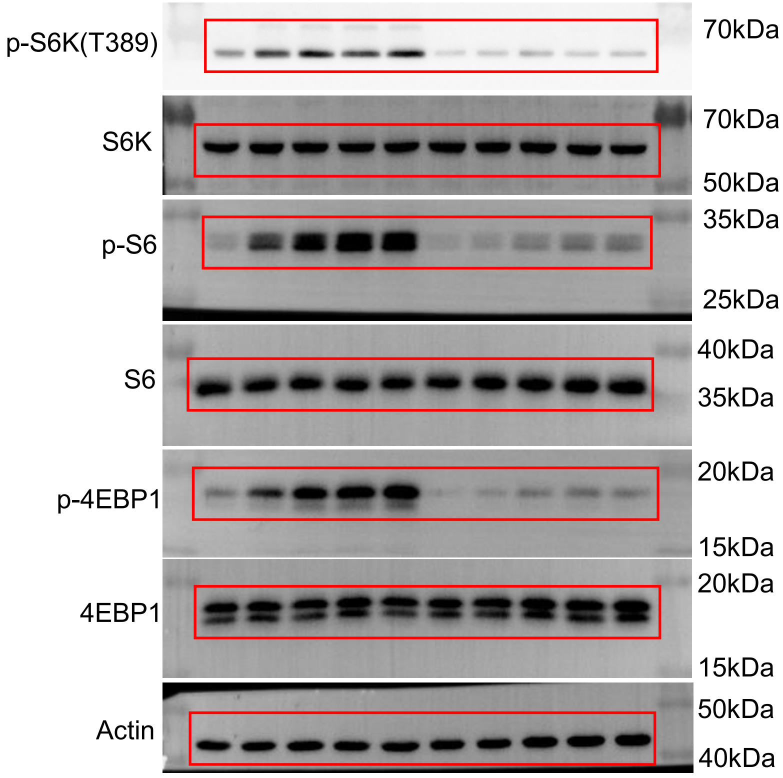

Full scans of blots for supplemental figure 16A

sFig 17C

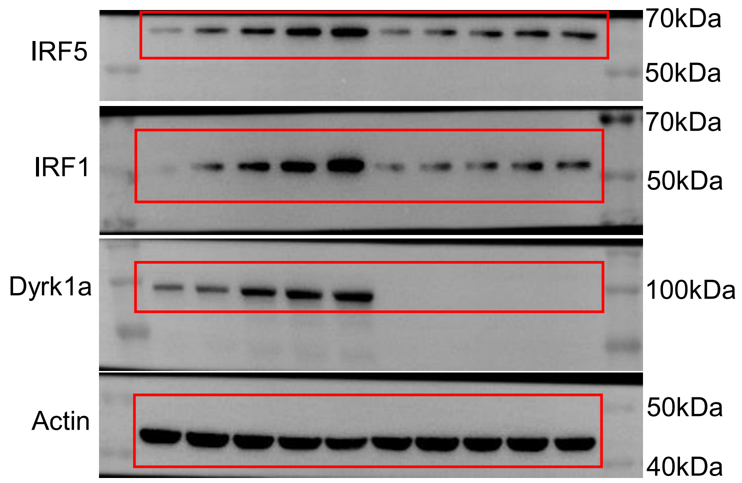

sFig 17D

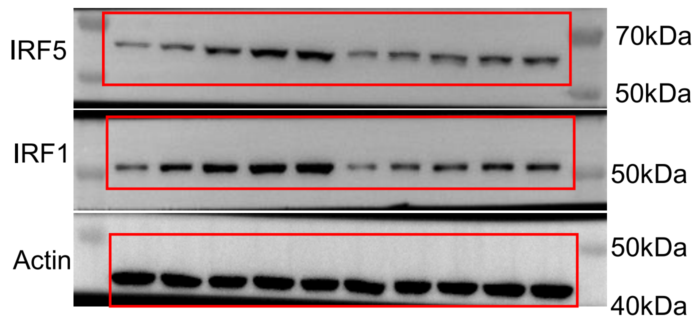

sFig 17F

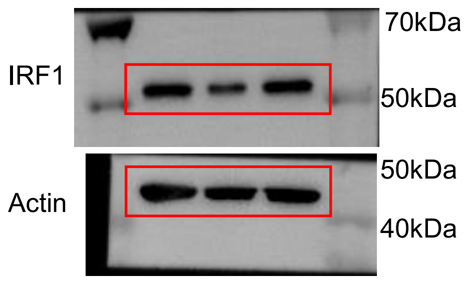

Full scans of blots for supplemental figure 17C-17D and 17F

sFig 18C

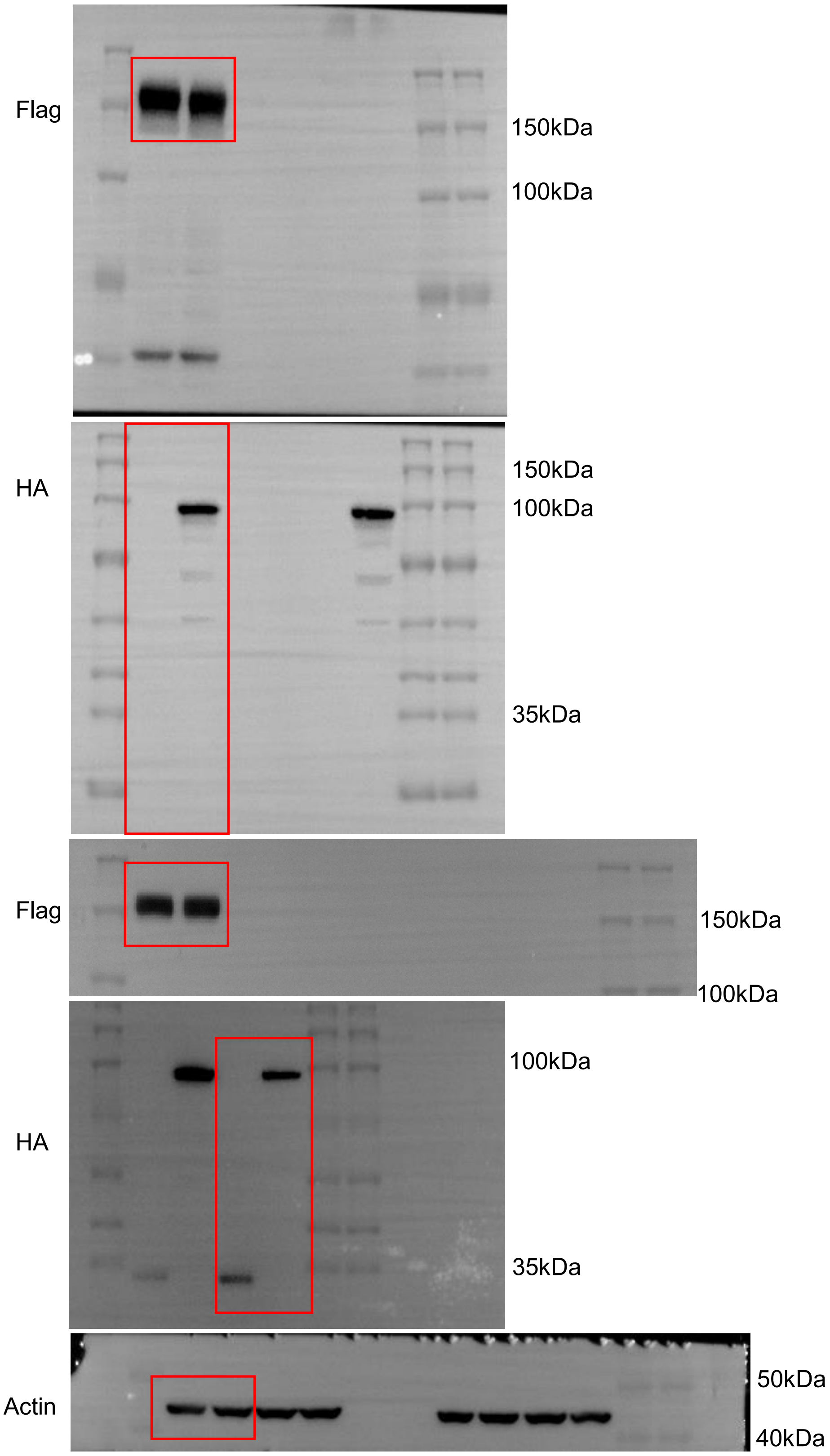

sFig 18D

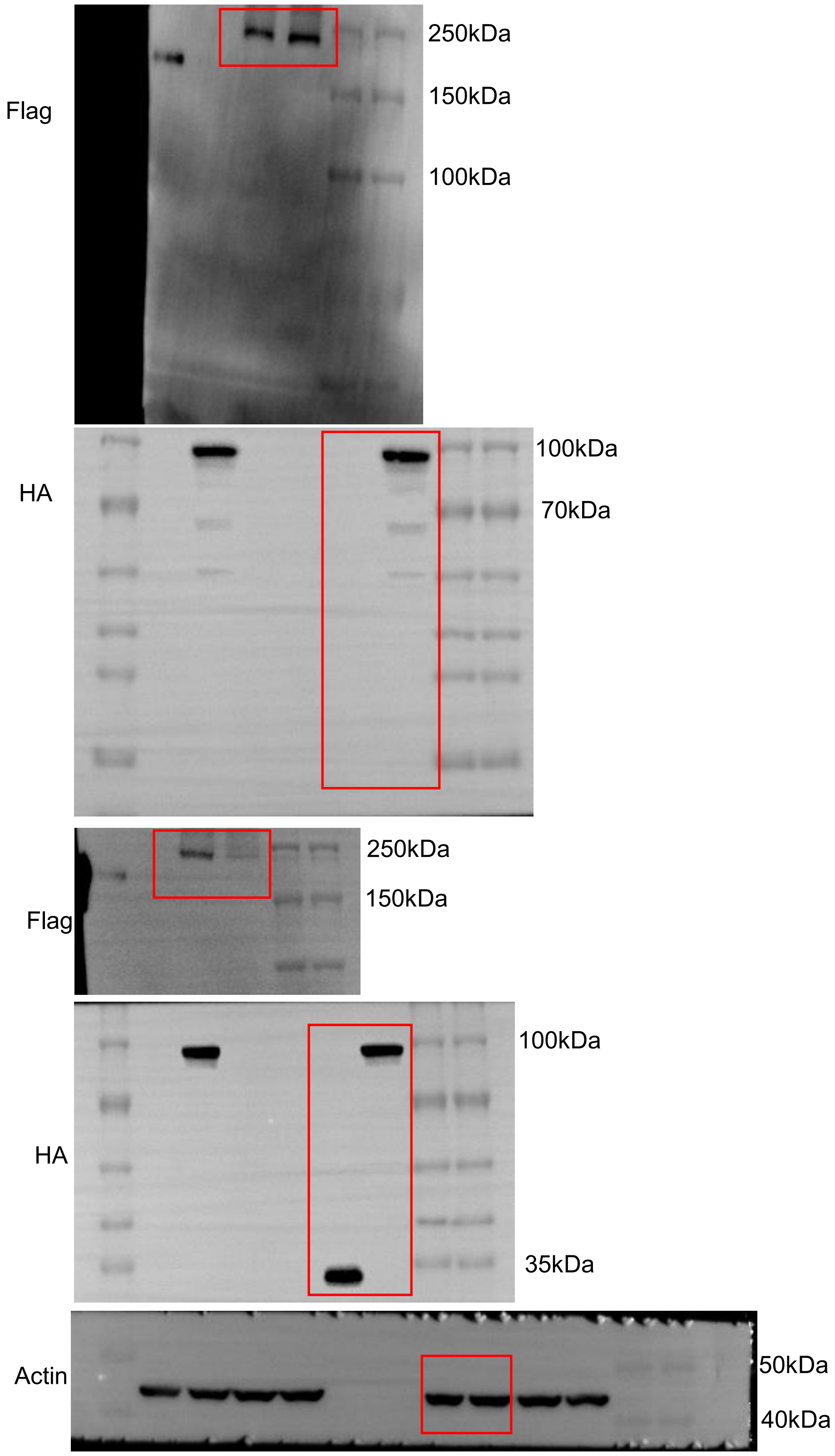

Full scans of blots for supplemental figures 18C-18D

sFig 19A

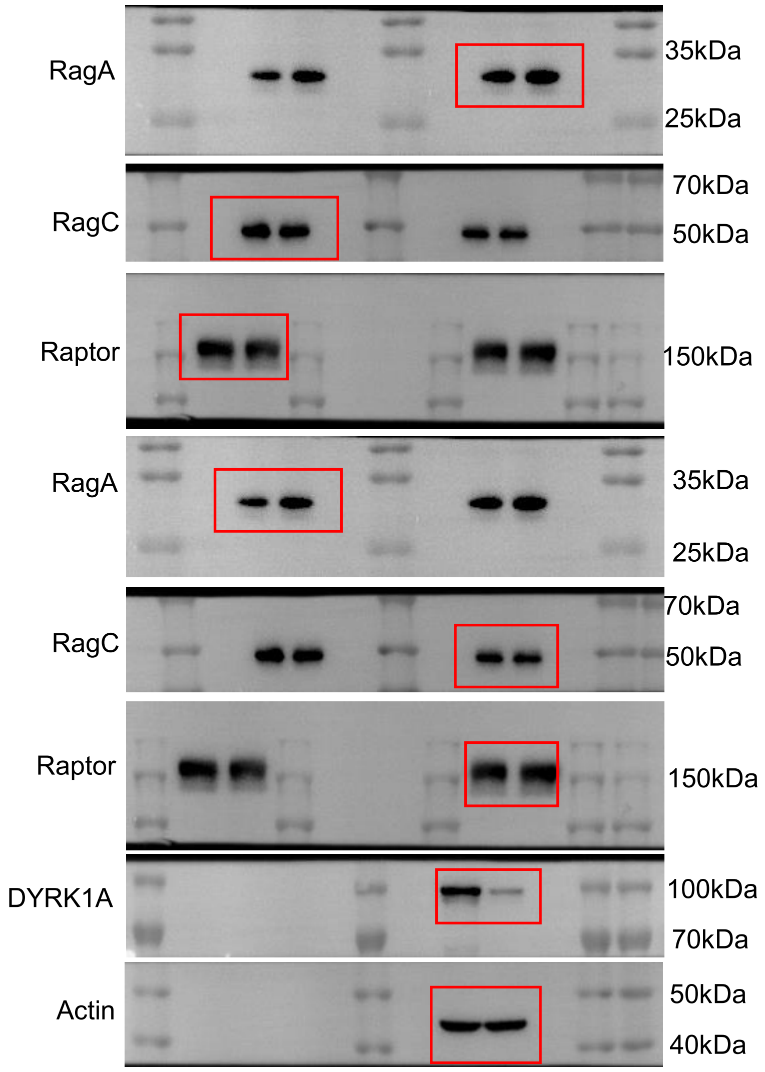

Full scans of blots for supplemental figure 19A

sFig 20A

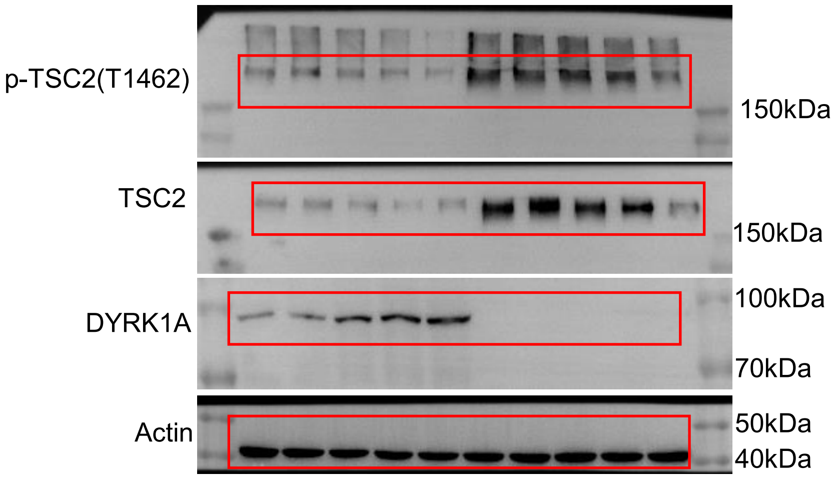

Full scans of blots for supplemental figure 20A

sFig 21A

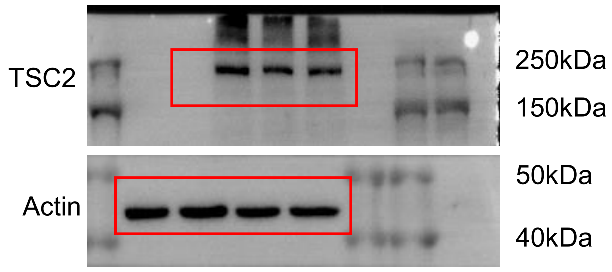

Full scans of blots for supplemental figure 21A

sFig 22A

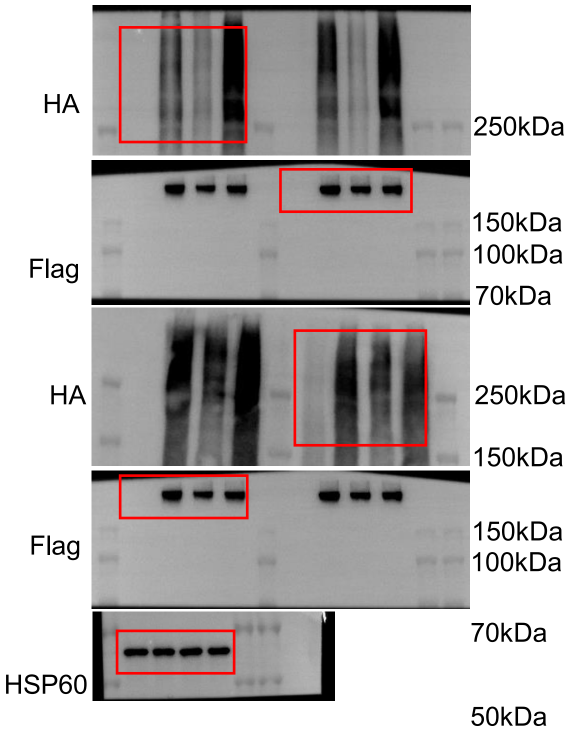

sFig 22B

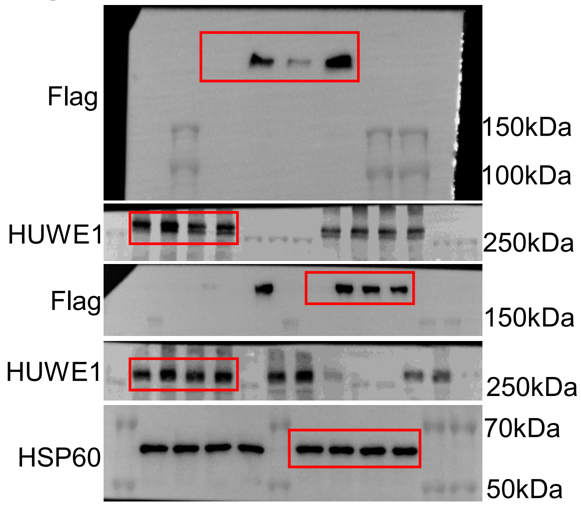

sFig 22C

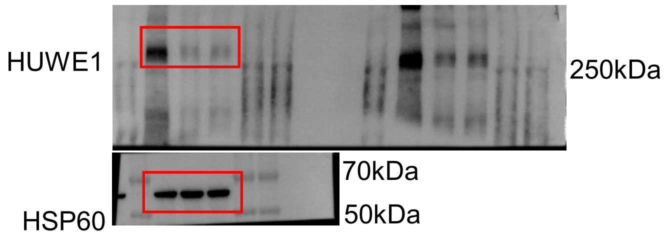

sFig 22D

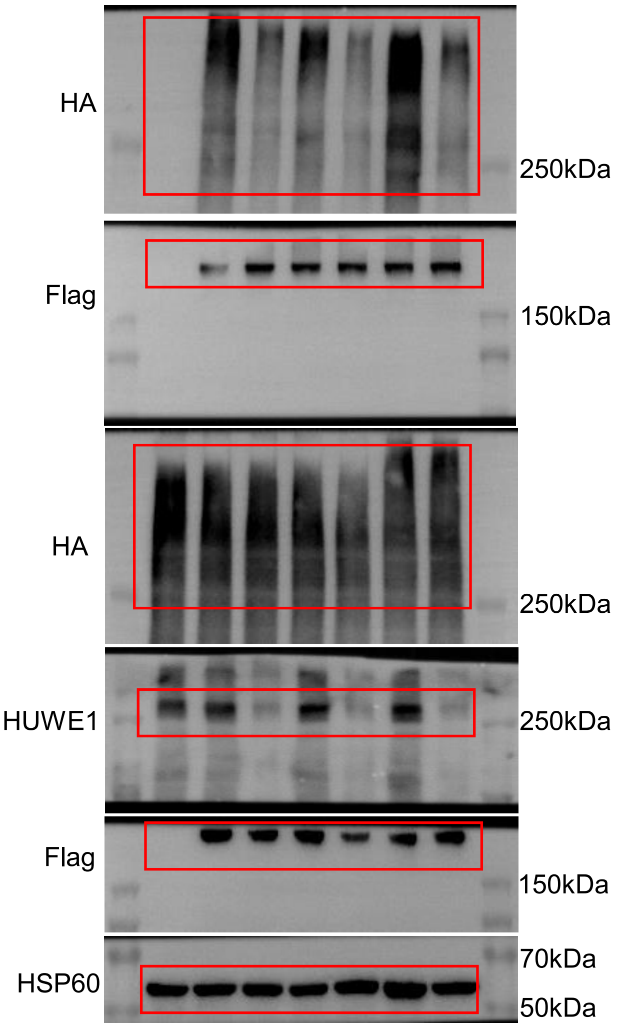

sFig 22E

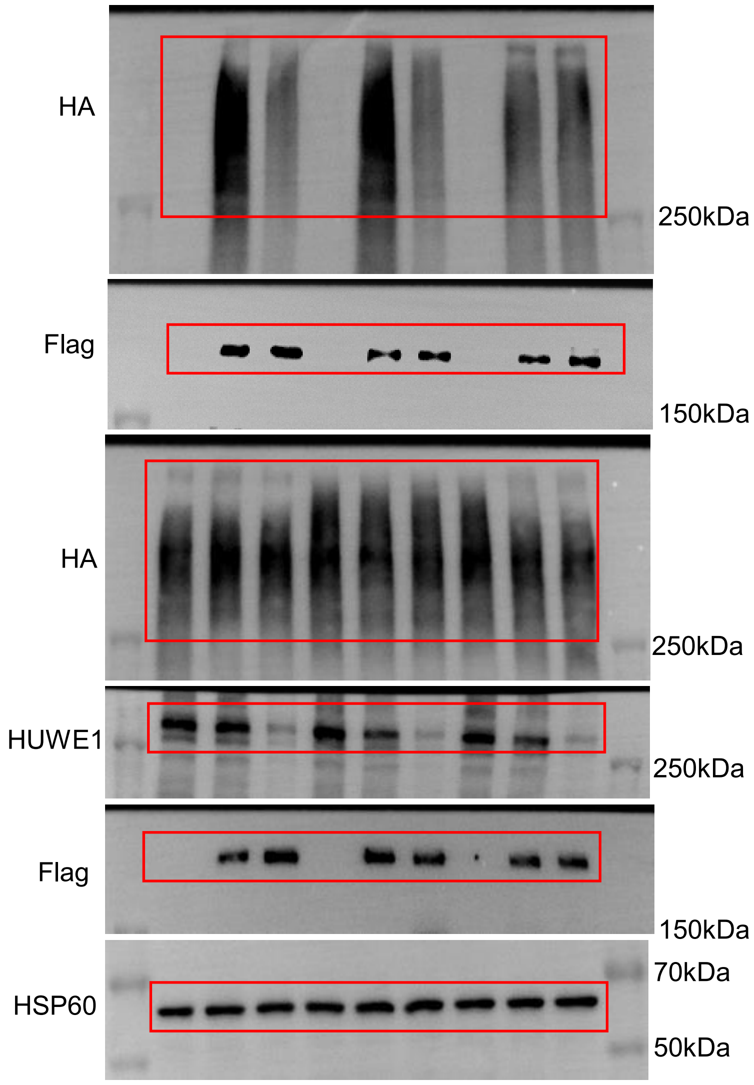

sFig 22F

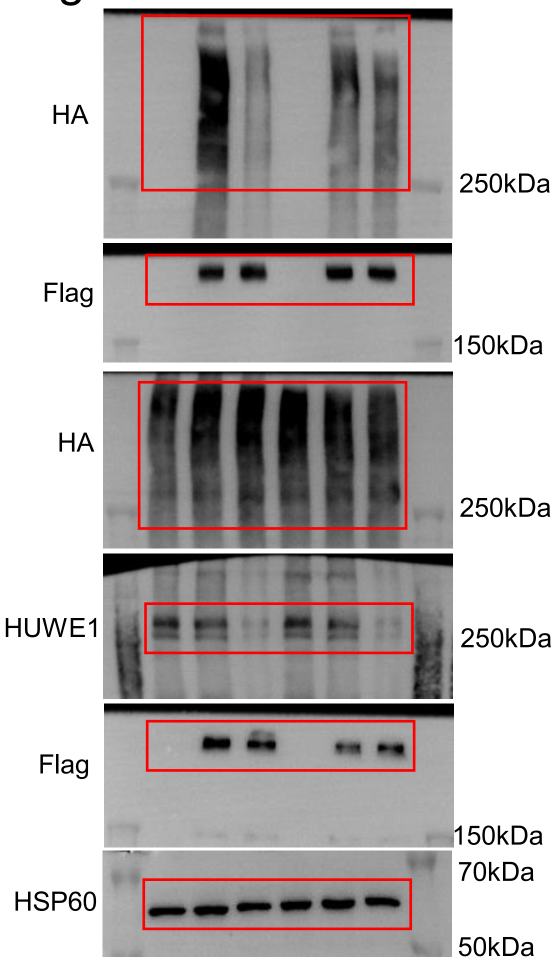

Full scans of blots for supplemental figures 22A-22F

sFig 23E

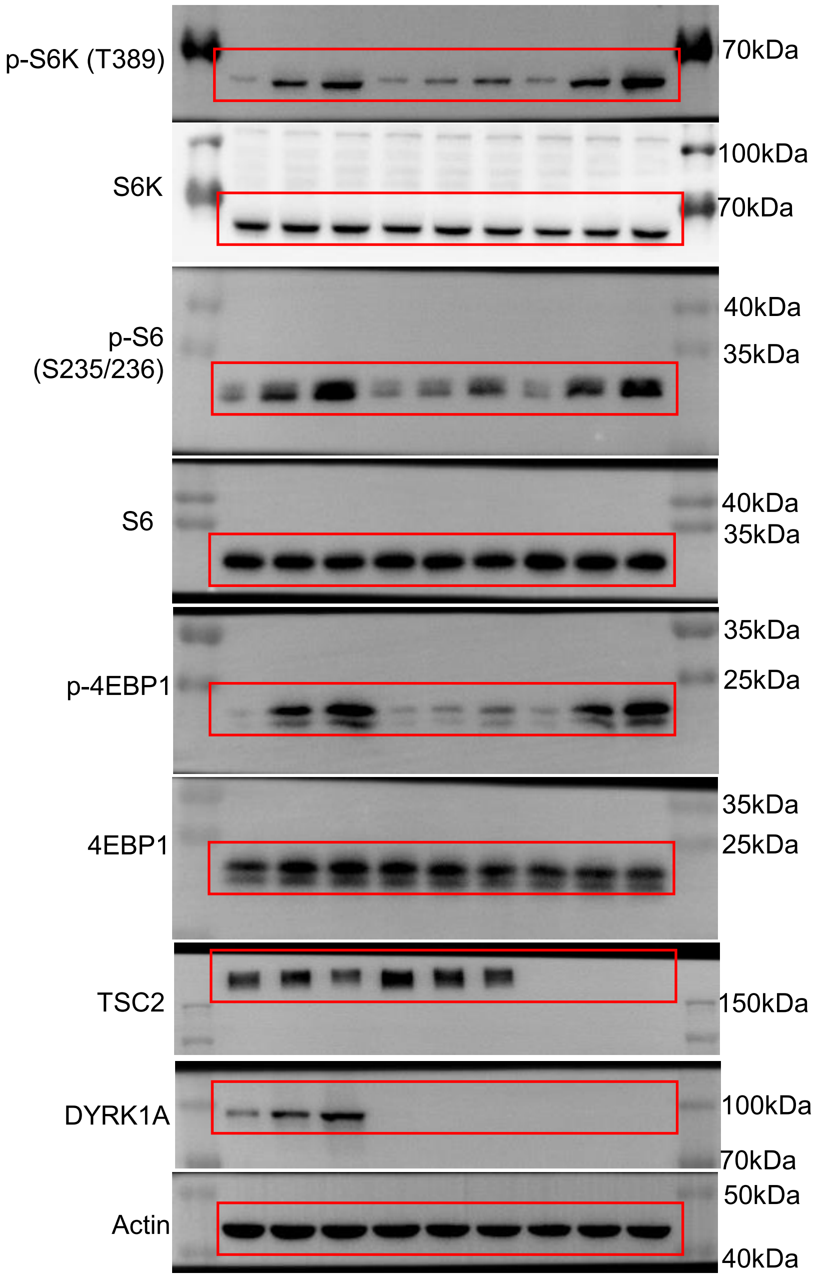

Full scans of blots for supplemental figure 23E
